# Wavelength-Selective Reactivity of Iron(III) Halide Salts in Photocatalytic C–H Functionalization

**DOI:** 10.1021/acs.joc.4c03107

**Published:** 2025-02-24

**Authors:** Cory T. Ludwig, Isiaka A. Owolabi, Logan W. Evans, Gabriel J. Smith, Alexander Ramos, James J. Shepherd, David B.C. Martin

**Affiliations:** † Department of Chemistry, University of Iowa, Iowa City, Iowa 52242, United States; ‡ Department of Chemistry, Michigan State University, East Lansing, Michigan 48824, United States

## Abstract

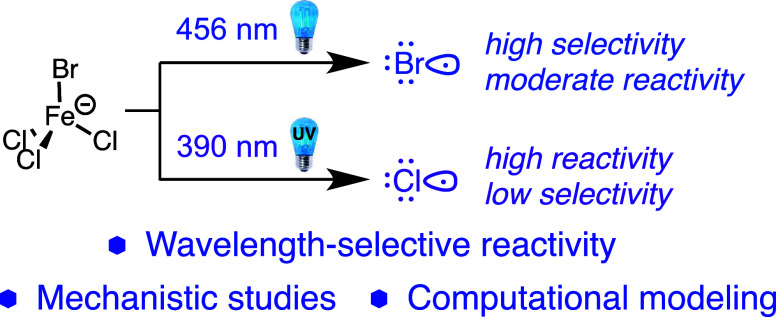

The utility of halogen radicals in hydrocarbon functionalization extends from early examples of photochemical halogenation to recent reports using photoredox catalysis with iridium complexes and simple transition metal salts such as FeCl_3_. The majority of these methods (uncatalyzed and iron-catalyzed) require UV light (λ ≤ 390 nm), and systematic efforts to enable the use of visible light remain valuable. We report the use of a simple Fe(III) salt that enables a C–H to C–C and C–N functionalization under visible light. The reactivity and selectivity profile using different light sources demonstrates wavelength-selective behavior, which was further investigated with deuterium kinetic isotope effect experiments and DFT calculations. These results show that control over the reactive intermediates in this iron-catalyzed reaction can be achieved through proper choice of the wavelength of irradiation.

## Introduction

The direct conversion of C–H bonds to C–C bonds continues to be an important goal in organic synthesis and a challenging testing ground for the field of catalysis.^1,2^ These methods have the capacity to streamline synthetic schemes and enable new strategies to molecules of interest across all areas of synthesis.^3^ While numerous approaches for the activation of C–H bonds are known, radical H atom transfer (HAT) remains one of the most powerful for C(sp^3^) centers due to favorable thermodynamics with heteroatom abstractors and the versatility of the resulting carbon-centered radicals.^4,5^ Light-driven HAT methods are particularly suited for this process.^6,7^ Photoredox activation has proven to be especially effective, even allowing the functionalization of strong C–H bonds, as highlighted by the selective functionalization of adamantanes and diamondoids (C–H bond dissociation energies BDEs >95 kcal/mol) reported by our group, Albini, Schreiner and Fokin, Nicewicz and Alexanian, MacMillan, Barriault, Knowles and Alexanian, and others.^8−16^ The widespread use of iridium- and ruthenium-based photocatalysts in indirect HAT methods has its drawbacks, such as their low abundance and high cost. While organic alternatives are increasingly available, they can suffer from lower stability than their transition metal counterparts in the presence of nucleophilic alkyl radicals.^17,18^

**Figure 1:**
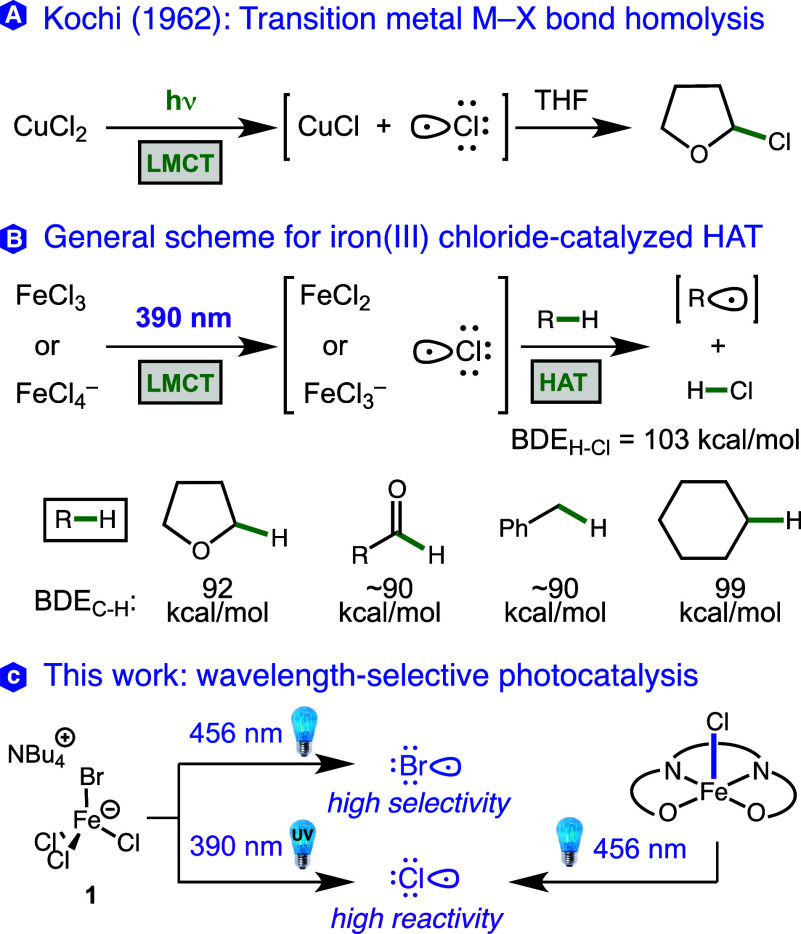
Examples of photochemical chlorine production and strategies for wavelength-selective photocatalytic C–H reactivity.

**Figure 2:**
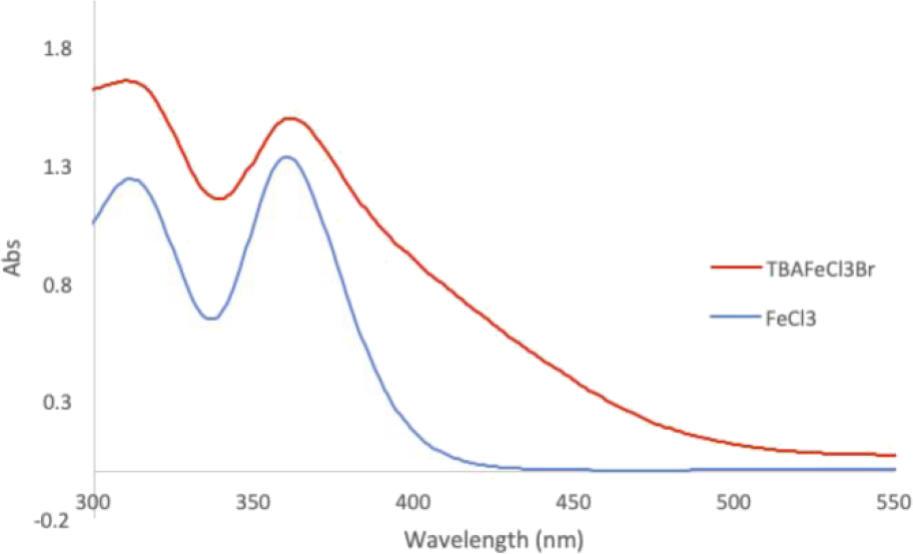
UV–vis absorption data for FeCl_3_ and TBA[FeCl_3_Br] (**1**), 0.3 mM in CH_3_CN.

As far back as the 1960s, the work of Kochi demonstrated that first row transition metal salts such as copper chloride could produce reactive radicals under photochemical conditions (Figure 1A).^19^ Later studies showed that iron trichloride could similarly activate C–H bonds under catalytic conditions.^20,21^ Recent years have seen a resurgence in interest in using iron trichloride as a source of chlorine radical via ligand-to-metal charge transfer (LMCT), including applications in C–C bond formations, C–X bond formation, selective oxidations, and polymer degradation (Figure 1B).^22−38^ The vast majority of these reactions require the use of ultraviolet (UV) light with wavelengths ≤390 nm, and this is easily understood by looking at the UV–vis absorption spectrum of FeCl_3_ (Figure 2, blue trace). The absorption peaks at 360 nm and tails off significantly above 400 nm, leading to very little reactivity using visible light sources, consistent with our observations below. In 2018, however, Jiang and co-workers reported the synthesis of a crystalline salt TBA[FeCl_3_Br] (**1**, TBA = tetra-*n*-butylammonium) that facilitated a benzylic oxidation reaction under visible light (blue LED), demonstrating the possibility of shifting the reactivity of iron trichloride into the visible spectrum (Figure 2, red trace).^39^ The proposed mechanism put forth by Jiang and others^40^ for this catalyst does not involve chlorine radicals, but rather single electron oxidation of the substrate and reduction of dioxygen. The effect of this new ligand sphere on the mechanism of LMCT processes has not been investigated, for example, whether chlorine radicals are still generated as a reactive species (as proposed for FeCl_3_)^20,21,41^ or if this behavior is dependent on the presence or absence of O_2_. Herein, we report our findings on the effect of different ligands (including multidentate ligands) on the light absorption properties of iron chloride salts and their utility in a direct C–H to C–C alkylation reaction mediated by halogen radicals.^42^ We describe the observation of wavelength-dependent reactivity and selectivity outcomes (Figure 1C) that may be general in other contexts and provide experimental and computational evidence to support our mechanistic proposal.

## Results and Discussion

We began our investigations by exploring conditions with iron trichloride under 390 and 456 nm irradiation (Table 1). The excellent reactivity of FeCl_3_ with 390 nm light (complete conversion in <2 h) was greatly diminished by switching to blue light (entry 1 vs entries 2 and 3). The reactivity was partially restored by incorporation of a catalytic amount of LiBr (entry 4) or the preformed Li[FeCl_3_Br] salt (entry 5), providing to up to 92% yield over 16 h.^39^ A significant disadvantage of these reactions is the need to weigh out FeCl_3_ or lithium salts, both of which are somewhat hygroscopic, and Li[FeCl_3_Br] is isolated as a highly viscous liquid. These properties lead to difficulties in accurate measurements and variability in the yields. In contrast, the preformed complex first reported by Jiang, TBA[FeCl_3_Br] (**1**), is a nonhygroscopic solid and consistently provided the best yields under blue light irradiation (entry 6, 94% yield).^39^ Catalyst **1** was less efficient in CH_2_Cl_2_ compared to CH_3_CN (entry 7, 71%). The reactions in Table 1 were all carried out under air with no effort to exclude atmospheric oxygen; however, comparable yields were achieved under a N_2_ atmosphere, demonstrating that the presence of O_2_ is not required for visible light reactivity and the alkylation reaction tolerates O_2_. Control reactions show that both the iron catalyst and light are required for this transformation (entries 9–10).

**Table 1:**
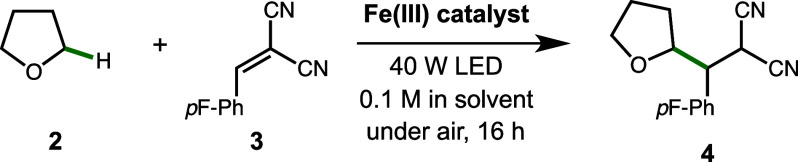
Optimization Studies of C–H Alkylation Using Simple Iron(III) Halide Salts^a^

entry	Fe(Ill) catalyst	LED (nm)	solvent	yield (4, %)
1	10 mol % FeCl_3_	390	CH_2_Cl_2_	80–90
2	10 mol % FeCl_3_	456	CH_2_Cl_2_	48
3	10 mol % FeCl_3_	456	CH_3_CN	6
4	10 mol % FeCl_3_ + LiBr	456	CH_3_CN	71–82
5	5 mol % Li[FeCl_3_Br]	456	CH_3_CN	82–92
6	5 mol % NBu_4_[FeCl_3_Br]	456	CH_3_CN	94 (85)
7	5 mol % NBu_4_[FeCl_3_Br]	456	CH_2_Cl_2_	71
8	1 mol % NBu_4_[FeCl_3_Br]	456	CH_3_CN	67
9	5 mol % NBu_4_[FeCl_3_Br]		CH_3_CN	0
10		456	CH_3_CN	0

a Reactions performed on a 0.2 mmol scale using one 40W Kessil LED lamp over 16 h with fan cooling using a Hepatochem PhotoRedOx box. Yields determined by GC using benzodioxole as an internal standard. Isolated yield in parentheses.

We also investigated other ligands to shift the absorbance of iron chloride species into the visible spectrum. Based on the hypothesis that conjugated ligands such as salen and salophen could redshift the LMCT absorption, as well as our previous experience with the photochemistry of cobalt salens and salophens, we investigated a small library of these Fe(III) monohalide complexes (Table 2).^43,44^ We observed low to moderate reactivity with iron salen complexes (**5**–**7**, up to 64% yield), while the salophen complex **8** showed excellent reactivity (entry 4, 83% yield). The corresponding Fe(III) bromide complexes (e.g., **9**) did not show significant reactivity under the conditions tested. While salen and salophen complexes provide a potentially useful and novel approach to enable iron catalysis using visible light, they were not as efficient or robust as the readily available ferrate salt **1**, which was chosen for further investigation.

**Table 2:**
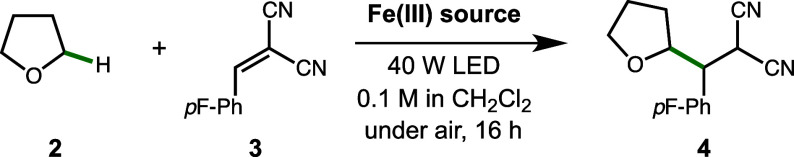
Optimization Studies with Polydendate Ligands^a^

entry	Fe(Ill) source	LEDs (nm)	yield (4, %)
1	10 mol % FeCl(salen) **5**	456	7
2	10 mol % FeCl(salen) **6**	456	29
3	10 mol % FeCl(salen) **7**	456	64
4	10 mol % FeCl(salph) **8**	456	83
5	10 mol % FeBr(salph) **9**	456	<5

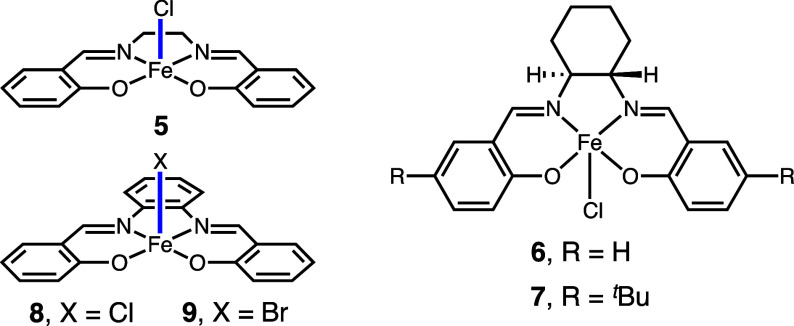

a Reactions performed on a 0.2 mmol scale using one 40 W Kessil 456 nm lamp over 16 h with fan cooling. Yields determined by GC using benzodioxole as an internal standard.

**Scheme 1:**
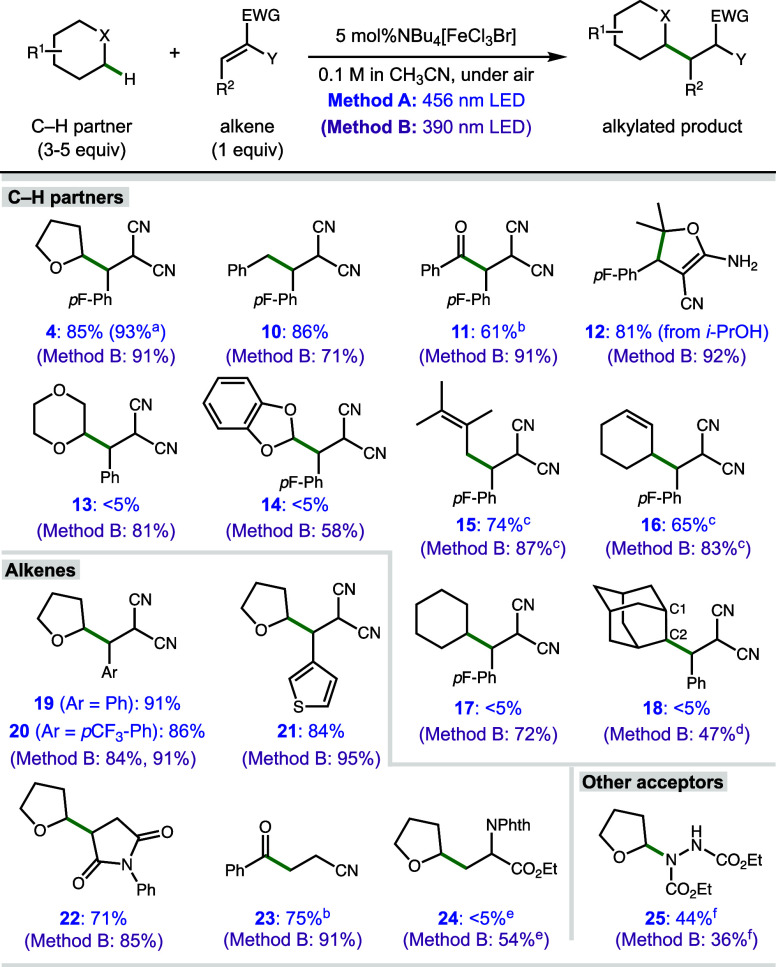
Substrate Scope of Iron-Catalyzed Functionalization 1.5 mmol scale reaction, 144 h. Reaction time for aldehydes is 36 h. Reaction performed under a N_2_ atmosphere with 15 mol % **1**. Reaction time is 72 h. Reaction time is 60 h. Product formed as a 1.4:1 mixture of regioisomers (C2:C1); determined by ^1^H NMR. With air balloon (see SI for details). Yield determined by ^1^H NMR. Standard reaction time is 16 h.

With optimized conditions established, we explored the scope of the C–H alkylation procedure with TBA[FeCl_3_Br] **1** under visible light irradiation and using 390 nm LEDs as a point of comparison, beginning with the C–H partner (Scheme 1). The alkylation product of THF **4** was produced in 85% isolated yield using 456 nm LEDs (Method A), which improved slightly at a larger scale (93%), while other activated C–H partners showed good to excellent efficiency (**10**-**12**, 61–86% yield). Notably, certain ethers, benzylic substrates, a secondary alcohol, and aldehydes were particularly efficient, with aldehydes requiring longer reaction times (36 h). Product **12** results from cyclization of the alcohol onto the nitrile after the radical addition, as observed in a related reaction.^45^ Activated substrates such as 1,4-dioxane and benzodioxole were not alkylated under visible light irradiation, which we initially attributed to strong binding to iron. Activated allylic substrates were successful (**15**-**16**, 65–74% yield); however, they only gave good results under a N_2_ atmosphere. Previous attempts with FeCl_3_ and UV light reported no desired product for such alkenes and a competing [2 + 2]-cycloaddition, demonstrating a significant advantage of catalyst **1** using visible light.^26^ Unactivated C–H partners such as cyclohexane and adamantane were unreactive using Method A, leading to the recovery of starting materials. Using TBA[FeCl_3_Br] under 390 nm LEDs (Method B, in parentheses), similar to slightly diminished yields were observed with THF and toluene as partners (**4** and **10**, 91 and 71% yields). Benzaldehyde showed a notable improvement (91% yield of **11** in 16 h), and problematic ether and acetal substrates became competent substrates using Method B (**13** and **14**, 81 and 58% vs <5% yield). Allylic substrates showed moderate improvement (**15**-**16**, 83–87% yield). Unactivated hydrocarbon partners also showed a dramatic improvement, providing alkylation products in moderate to good yields (**17**–**18**, 47–72% yield). Adamantane, with its particularly strong C–H bonds (BDEs = 96 and 99 kcal/mol),^46^ was less efficient and gave a mixture of C2 and C1 substitution products with 1.4:1 r.r. after extended reaction time. The significant expansion in scope based on wavelength is an important indicator of the mechanistic change that emerged upon further investigation (vide infra).

A variety of radical acceptors were effective in this reaction under visible light (Scheme 1, bottom). Alkylidene malononitriles with different aryl substituents were excellent partners (**19**-**21**, 84–91% yield), including the electron-rich heterocycle thiophene. *N*-Phenylmaleimide and acrylonitrile were also suitable alkene acceptors (**22**-**23**, 71–75% yield). Yields for different acceptors using Method B were generally slightly better (**19**-**23**, 84–95% yield); a dehydroalanine derivative was productive only with Method B, leading to product **24** in 54% yield. Alkylidene malonates were also ineffective using Method A (<5% yield, see SI). We attribute these failures to coordination of the carbonyls to iron, affecting the MLCT process at visible wavelengths; however, it could be related to the turnover step required to regenerate the ferrate ion **1**.^28^ C–N bond formation could also be accomplished with visible light using diethyl azodicarboxylate as a substrate (**25**, 44% yield by ^1^H NMR), a slight improvement over irradiation with 390 nm light (36%).^28,29^

The results in Scheme 1 gave us some initial insight into the mechanistic features of the mixed iron halide catalyst. Many of the reactions in Scheme 1 can be performed under UV light (390 nm) with TBA[FeCl_3_Br] as well as with FeCl_3_ as expected from previous reports (see the SI for selected results). Reactions are generally a little slower with blue light; however, some notable differences in scope suggested a more significant change in reactivity. For example, while cyclohexane is an excellent substrate under UV light, almost no alkylation occurs at 456 nm over extended irradiation (Table 3, entries 1 and 2 vs entry 3). The catalyst is not converted to an inactive form, because irradiation with 390 nm light after 16 h of blue light leads to complete conversion to give the expected product (entries 4 and 5). We questioned whether the loss of reactivity was related to a change in the outcome of LMCT at different wavelengths, namely, whether the longer wavelength could lead to the preferential formation of bromine radicals rather than chlorine radicals. The initial report by Jiang and co-workers primarily used benzylic substrates for the oxidation reaction, and the weaker C–H bonds of these substrates could presumably be activated by either halide.^39^ The literature values for the BDE of the H–Cl bond (103 kcal/mol) and H–Br bond (88 kcal/mol) support this possibility.^47,48^

**Table 3:**
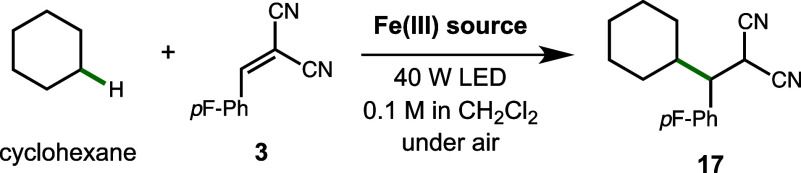
Catalyst and Light Source Studies with Cyclohexane^a^

entry	Fe(Ill) source	LEDs (nm)	yield (17, %)
1	10 mol % FeCl_3_	390	52
2	5 mol % NBu_4_[FeCl_3_Br]	390	71
3	5 mol % NBu_4_ [FeCl_3_Br]	456	<3
4	10 mol % FeCl_4_	456 then 390^b^	68
5	5 mol % NBu_4_ [FeCl_3_Br]	456 then 390^b^	61

a Reactions performed on a 0.2 mmol scale using one 40 W lamp over 16 h with fan cooling. Yields determined by GC using benzodioxole as an internal standard.

b Reactions run for 16 h at 456 nm (<3% product) and then run for 16 h at 390 nm (GC yield shown).

Given the well-known differences in selectivity for traditional alkane radical halogenation with chlorine and bromine radical, we employed cyclopentylmethyl ether (CPME) as a test substrate. CPME was previously used by Barriault and co-workers to test the influence of solvent on the reactivity of chlorine and bromine radicals in a related transformation using an iridium photocatalyst.^14^ As shown in Table 4, using FeCl_3_ and 390 nm light gave a 1:1.29 ratio of products favoring the reaction at the methyl site (1°) over the 3° site. Even after adjustment for the difference in available C–H bonds (CH vs CH_3_), this outcome is indicative of the low discrimination of the highly reactive chlorine radical, as this is the only halogen present. Switching to TBA[FeCl_3_Br] and 456 nm light resulted in a 2.34:1 ratio of products, corresponding to a 7:1 ratio on a per-C–H bond basis. This is consistent with the higher discrimination expected for the bromine radical.^47^ The solvent identity had a relatively minor influence on the selectivity (2.0 to 2.9:1 ratio; see SI for details). Interestingly, using TBA[FeCl_3_Br] with 390 nm irradiation led to a 1:1.01 ratio of products, a result that is intermediate between the others. These results suggest that excitation of the [FeCl_3_Br]^−^ anion with 456 nm light primarily generates a bromine radical via LMCT while 390 nm light primarily, but not exclusively, generates chlorine radicals via LMCT.

**Table 4:**
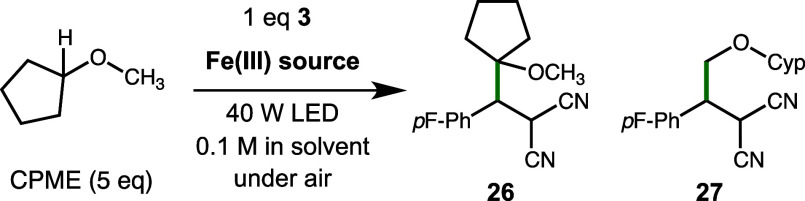
Selectivity Studies with Cyclopentylmethyl Ether^a^

entry	Fe(Ill) source	LEDs (nm)	ratio 26:27
1	10 mol % FeCl_3_	390	1:1.29
2	5 mol %NBu_4_[FeCl_3_Br]	456	2.34:1
3	5 mol %NBu_4_[FeCl_3_Br]	390	1:1.01

a Reactions performed on a 0.2 mmol scale using one 40 W lamp over 16 h with fan cooling. Solvent is CH_2_Cl_2_ for FeCl_3_ and CH_3_CN for catalyst **1**. Yields determined by GC using benzodioxole as an internal standard.

We performed deuterium kinetic isotope effect (KIE) experiments to probe the turnover-limiting step of the reaction. Parallel reactions using THF and *d*_8_-THF showed no significant difference in reaction rate using FeCl_3_ as the catalyst with 390 nm LEDs (KIE = 1.01 ± 0.03 at 30 min). This shows that C–H bond cleavage (HAT) is not the turnover-limiting step with the chlorine radical. A competition experiment with 5 equiv of THF and 5 equiv of *d*_8_-THF using FeCl_3_ as the catalyst showed a moderate KIE of 1.50 ± 0.03 at 30 min. Combined with the results above, this indicates that C–H bond cleavage is fast and irreversible, but not the turnover-limiting step when chlorine radical is the abstractor.^23,28,32^

We performed similar experiments using the mixed halide salt at 456 nm to find evidence of a bromine radical. Parallel reactions using THF and *d*_8_-THF showed a significant difference in reaction rate, giving a KIE = 2.69 ± 0.16 at 3 h. This shows that C–H bond cleavage (HAT) is the turnover-limiting step under these conditions. A competition experiment with 5 equiv of THF and 5 equiv of *d*_8_-THF using TBA[FeCl_3_Br] and 456 nm light showed a significant KIE of 6.5 ± 0.5 at 3 h, again consistent with a turnover-limiting HAT step.

Finally, we performed similar KIE experiments using a mixed halide salt at 390 nm. Parallel reactions using THF and *d*_8_-THF showed a small but significant difference in reaction rate, giving a KIE of 1.37 ± 0.12, a result that is intermediate between the two data sets given above. Similarly, a competition experiment with 5 equiv of THF and 5 equiv of *d*_8_-THF using TBA[FeCl_3_Br] and 390 nm light showed a significant KIE of 2.06 ± 0.15 at 30 min. Again, this result is intermediate between the two results given above, suggesting that the mixed catalyst TBA[FeCl_3_Br] produces both bromine and chlorine radical under 390 nm light, favoring the chlorine radical. This is consistent with the selectivity studies above and reinforces the proposal of wavelength-dependent reactivity of catalyst **1**.

**Figure 3:**
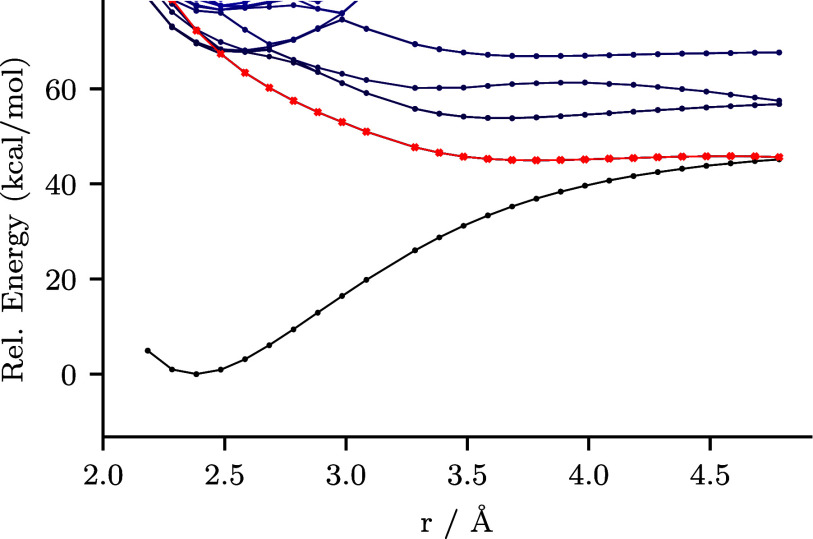
Computed potential energy surface along the Fe–Br bond distance of FeCl_3_Br^–^, where the ground state is plotted in black, and the state with a prominent contribution to Fe–Br σ* is indicated in red.

**Figure 4:**
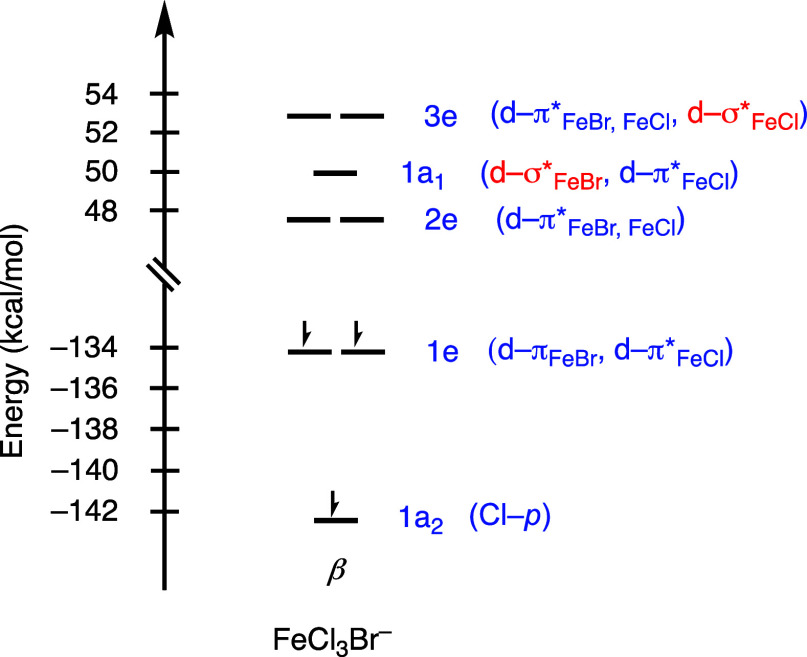
Quantitative energy level diagram for the β-electrons of FeCl_3_Br^–^ in C_3v_ symmetry. Symmetry labels and bonding interactions between the ligands and the metal center are listed.

Density functional theory (DFT)^49^ calculations were used to provide a mechanistic explanation for the difference between irradiating FeCl_3_Br^–^ at 456 nm versus 390 nm (technical details are presented in the SI). We followed a similar method to Dai et al.^26^ who showed that, in FeCl_4_^–^, irradiation by a 390 nm source caused photodissociation via a LMCT mechanism. At a calculated excitation corresponding to a wavelength of 378 nm, a hole–electron analysis showed that the excitation came from the Cl ligands to the Fe center. In our calculations for FeCl_3_Br^–^ (Table 5), we find that there is a peak similar to that of FeCl_4_^–^ at 374 nm with the same hole–electron analysis. However, FeCl_3_Br^–^ has additional features at 395.5, 394.7, and 411 nm, consistent with the appearance of the UV–vis absorbance spectrum (Figure 2), where the hole–electron analysis shows electron transfer during the excitation from the Br ligand to the Fe center. These follow a broader trend in the hole–electron analysis of the remaining excitations (Table S6) showing excitation from Br to Fe at longer wavelengths (411–362 nm) and from Cl to Fe at shorter wavelengths (374 to 301 nm). These results are consistent with the generation of mostly Br radicals at longer wavelengths and a mixture of radicals (including Cl radical) at shorter wavelengths. The blueshift in wavelength between the light source (456 nm) and the longest wavelength absorbance peak (411 nm) is about twice as large as Dai et al. (390 nm source/378 nm peak) and is attributed to the light source being nonmonochromatic and DFT performance (e.g., functional/basis set). DFT performance can cause variation by ±30 nm. To further analyze the dissociation, we generated a potential energy surface (PES) using a similar approach to those found in the literature.^50,51^ We performed stepwise TDDFT calculations along the dissociation coordinate formed by stretching the Fe–Br bond while maintaining C_3v_ symmetry (details in the SI). The first excited state at stretched bond distances contains a significant Fe–Br σ* molecular orbital character (Figure 3). This corresponds to an excitation at 395.5 nm at the equilibrium geometry, which has the lowest unoccupied natural transition orbital with an Fe–Br σ* appearance. Natural transition orbitals are orbitals that best represent the excitation when excitations include contributions from multiple molecular orbitals (see Table S7 for more details). This is consistent with the hole–electron analysis (Table 5). By contrast, we found that the first excitation to Fe–Cl σ* occurs at a lower wavelength of 362 nm. The hole–electron analysis shows that both Br and Cl contribute to the source of the excitation (Table S6). These observations are also consistent with the equilibrium-geometry molecular orbital diagram where the molecular orbital with a Fe–Br σ* character is lower in energy than the molecular orbital with the Fe–Cl σ* character (Figure 4).

**Table 5:** Tabulated Data for the Hole–Electron Analysis of FeCl_3_Br^–^ for the First Four Distinct Transitions with Strengths Greater Than **1***×***10**^*–***3**^^a^

λ	location	β e^–^	β h^+^
411 nm	Fe	83%	3%
	Br	7%	69%
	Cl ligands	10%	27%
395.5 nm	Fe	73%	4%
	Br	14%	73%
	Cl ligands	12%	24%
394.7 nm	Fe	83%	4%
	Br	6%	60%
	Cl ligands	11%	36%
374 nm	Fe	84%	1%
	Br	5%	3%
	Cl ligands	12%	95%

a The column **β e^*–*^** gives the location of the electron after the excitation and the **β h^+^** gives the location of the hole. The label **β** refers to the electron spin that is antiparallel to the unpaired electrons, normally taken to be spin down by convention. These locations are based on the atomic orbitals of each atom. Percentages may not add to 100% due to rounding.

**Figure 5:**
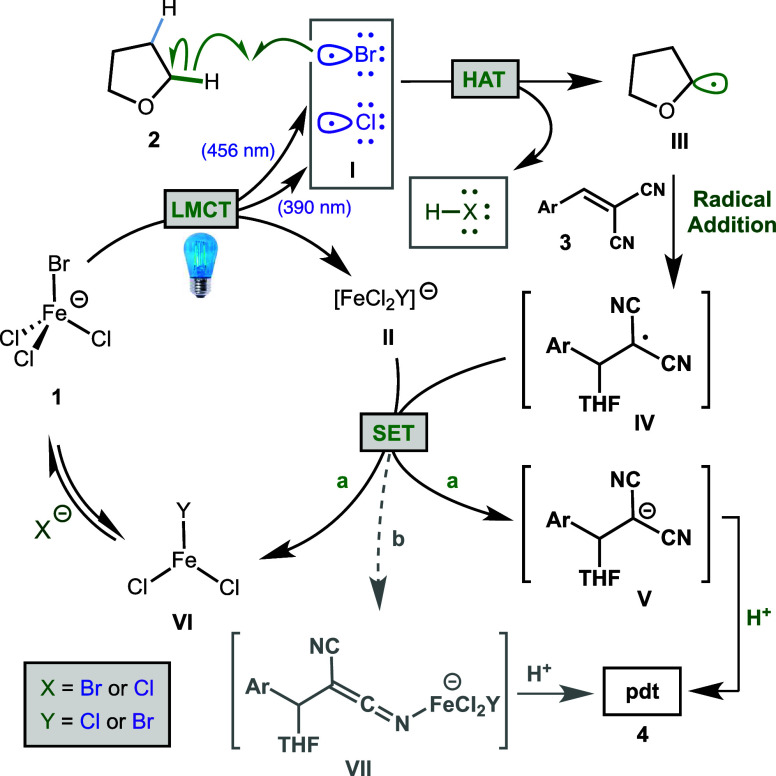
Proposed mechanism of photocatalytic activation.

The proposed mechanism for iron-catalyzed C–H functionalization is presented in Figure 5, beginning with the excitation of the tetrahedral Fe(III) catalyst through LMCT to produce halogen radical **I** and anionic Fe(II) byproduct **II**. The halogen radical undergoes HAT with hydrocarbon substrate **2**, generating the corresponding radical **III** and HX (hydrochloric or hydrobromic acid). Radical **III** reacts with the alkene partner to generate an electron-deficient radical **IV**, which can accept an electron from ferrate species **II** through a single electron transfer (SET, path a) to provide resonance-stabilized anion **V** and FeCl_3_ or FeCl_2_Br (**VI**). Protonation of the anion **V** by HX gives the observed product and coordination of the released halide ion regenerates the ferrate catalyst **1**. An alternative path from radical **IV** (path b) has been proposed by Rovis and co-workers, namely, recombination with the Fe(II) species to form an iron ketenimine **VII** (or analogous iron enolate).^23^ Protonolysis of this species would also deliver the product and FeCl_3_ or FeCl_2_Br (**VI**).

The key features of this proposed mechanism include the light-induced LMCT, which can accomplish the release of either chlorine or bromine radical **I**, depending on the excited state that is accessed, as described above. DFT calculations support that longer wavelength favor LMCT excitations from Br to Fe, while shorter wavelengths favored LMCT excitations from Cl to Fe. These calculations also show that an excitation to Fe–Br σ* occurs at a longer wavelength (395.5 nm) than Fe–Cl σ* (362 nm). The HAT step is also critical to the success of this reaction based on evidence that this is the turnover-limiting step with X = Br. The HAT step must be sufficiently favorable for the overall process to occur, and this is dependent on the H–X bond produced being stronger than the C–H bond cleaved. This is consistent with the success of cyclohexane (BDE_C–H_ = 99 kcal/mol) when using 390 nm light (BDE_H–Cl_ = 103 kcal/mol), compared to the very low efficiency when using 456 nm light that is proposed to produce a bromine radical (BDE_H–Br_ = 88 kcal/mol).^47^ The substrates that perform well with 456 nm light typically have a BDE_C–H_ of ≤92 kcal/mol and are electronically activated to provide a more hydridic C–H bond.^48^ The failure of some substrates such as 1,4-dioxane using blue light is less obvious based on BDE arguments, and we cannot exclude the possibility of some coordination to iron that disrupts LMCT at 456 nm. Additional experimental evidence is necessary to probe this possibility, but switching to higher energy light enables efficient access to this and other products using the same readily accessible, bench-stable Fe(III) catalyst.

## Conclusions

We have uncovered the wavelength-dependent reactivity of a simple, bench-stable iron catalyst that enables C–H functionalization of hydrocarbons with tunable selectivity. Investigation of the mixed halide catalyst TBA[FeCl_3_Br] has shown that irradiation at 390 nm leads primarily to a chlorine radical, similar to commonly used FeCl_3_, whereas irradiation with blue light at 456 nm leads essentially exclusively to a bromine radical, based on evidence from regioselectivity and KIE experiments and computational studies of the LMCT process. This wavelength-dependent behavior allows for control over the reactive intermediates in this iron-catalyzed reaction through the proper choice of the light source, which may have applications beyond simple C–C and C–N bond forming reactions described here. Future research efforts will be focused on fully elucidating the factors governing the efficiency and selectivity of light-induced halogen radical formation and new applications of C–H activation methods.

## Experimental Section

### General Information

All reactions were carried out using oven-dried or flame-dried glassware charged with a magnetic stir bar and conducted under an inert nitrogen atmosphere using typical Schlenk techniques, unless otherwise noted. All solvents were dried by passage through columns of activated alumina, distilled, and stored under nitrogen over freshly activated 4 Å sieves or otherwise freshly distilled. All starting materials were prepared according to known literature procedures or used as obtained from commercial sources unless otherwise indicated. Reactions were monitored by thin-layer chromatography (TLC) and carried out on 0.25 mm coated commercial silica gel plates (F254 precoated glass plates) using UV light as a visualizing agent. Unless otherwise indicated, silica gel chromatography was performed by using flash chromatography on P60 silica. See the Supporting Information for additional details.

### Safety Statement

**Caution!** This procedure uses a 40 W LED for irradiation. Care should be taken to protect the system from exposure to heat and intense light. Tinted glasses are recommended for working with light sources.

### General Procedure for Photochemical Reactions with TBA[FeCl_3_Br]

A typical procedure for a photoreaction with the tetrabutylammonium iron trichloride bromide salt can be conducted as follows: C–H donor (3.0 mmol, 5.0 equiv), TBA[FeCl_3_Br] (10 mol %), radical acceptor (0.60 mmol, 1.0 equiv), and dry acetonitrile (6.0 mL, 0.1 M) were added to an 8 mL vial equipped with a supermicro stir bar. The vial was capped with the manufacturer provided cap and oxygen was not excluded. The vials were then stirred at room temperature for 16–48 h under irradiation of either 390 or 456 nm LEDs. The reaction mixture was then concentrated in vacuo, and the resulting residue was purified using a column chromatograph. All isolated products were further characterized by 1H and 13C{1H} nuclear magnetic resonance (NMR) spectroscopy, infrared spectroscopy, and mass spectrometry.

## Data Availability

The data underlying this study are available in the published article and its Supporting Information.

## Supporting Information Available

Experimental procedures, characterization data, and ^1^H and ^13^C NMR spectra of all new compounds (PDF)
