# Supplementary material for: Wavelength-Selective Reactivity of Iron(III) Halide Salts in Photocatalytic C–H Functionalization
Source: J Org Chem. 2025 Feb 24;90(9):3404–11. doi: 10.1021/acs.joc.4c03107 (PMC11894668; doi:10.1021/acs.joc.4c03107)

Supporting Information

Wavelength-Selective Reactivity of Iron(III) Halide Salts in Photocatalytic C-H Functionalization

Cory T. Ludwig[a], Isiaka A. Owolabi[a], Logan W. Evans[a], Gabriel J. Smith[b], Alexander Ramos[a], James J. Shepherd[b], and David B.C. Martin[a]*

*Corresponding Author : david-martin@uiowa.edu

[a]Department of Chemistry, University of Iowa, Iowa City, Iowa 52242, United States.

[b]Department of Chemistry, Michigan State University, East Lansing, Michigan 44824, United States

Contents

General Methods: S2

General Photochemical Methods: S4

Safety Statement S6

A. General Procedures S6

I. General Procedure: photochemical reactions with TBA[$FeCl_3Br$]: S6

II. Synthesis of radical acceptor benzylidene malononitrile derivatives: S6

III. Synthesis of TBA[$FeCl_3Br$]: S7

IV. Synthesis of Iron salen/salophen Catalysts: S7

V. Iron salen/salophen results S7

VI. UV-Vis Studies S8

B. Optimization Details S9

I. General Procedure for the optimization of photochemical reactions with TBA[$FeCl_3Br$]:S9

II. Optimization Results S10

C. Mechanistic Studies S11

I. Kinetic Isotope Effect Results S11

II. Catalyst Selectivity Results S15

D. Characterization Data of Products S16

E. Computational Methods S25

F. References S29

G. NMR Spectra S31

General Methods:

All reactions were carried using oven dried or flame dried glassware charged with a magnetic stir bar and conducted under an inert nitrogen atmosphere using typical Schlenk

techniques, unless otherwise noted. All solvents were dried by passage through columns of activated alumina or distilled and stored under nitrogen over freshly activated 4 Å sieves or otherwise freshly distilled. All starting materials were prepared according to known literature procedures or used as obtained from commercial sources, unless otherwise indicated. Reactions were monitored by thin-layer chromatography (TLC) and carried out on 0.25 mm coated commercial silica gel plates (Analtech TLC Uniplates, F254 precoated glass plates) using UV light as visualizing agent. Unless otherwise indicated, silica gel chromatography was performed using flash chromatography on P60 silica.

^{1}H and ^{13}C NMR spectra were recorded on a Bruker Avance NEO 400, Bruker Avance III 500, or Bruker Avance III 700 MHz spectrometer and were internally referenced to residual protio solvent signal (note: $CDCl_3$ referenced at δ 7.26 ppm for ^{1}H NMR and δ 77.16 ppm for ^{13}C NMR, respectively). Data for ^{1}H NMR are reported as follows: chemical shift (δ ppm), multiplicity (s = singlet, d = doublet, t = triplet, q = quartet, m = multiplet, app=apparent), coupling constant (Hz), and integration. Data for ^{13}C{^{1}H} NMR are reported in terms of chemical shift and no special nomenclature is used for equivalent carbons. High-resolution mass spectrometry (HRMS) data were recorded on a Thermo Q-Exactive instrument (hybrid quadrupole Orbitrap) using direct injection of samples in dichloromethane diluted into acetonitrile/water (1:1) into the electrospray source (ESI) with positive ionization or negative ionization (as noted), listed as LC-HRMS, or on an Agilent 7250 GCMS Q-TOF instrument with electron ionization (EI) using direct injection of samples in dichloromethane, listed as GC-HRMS. Gas Chromatography was carried out using a Shimadzu GC-2010 Plus instrument equipped with a Shimadzu SH-Rxi-5ms column. Column Specifications: 15 m (L), 0.25 mm (ID), 0. 25 µm (d_f), (diphenyl/dimethyl polysiloxane) stationary phase. UV-Vis experiments were carried out with a Agilent Cary 5000, with a total wavelength range of 175-3300 nm, but collecting data from 200 nm to 800 nm.

General Photochemical Methods:

A typical setup for a photoreaction in the Martin Lab utilizes an EvoluChem PhotoRedOx Box from Hepatochem (part number HCK1006-01-016, https://hepatochem.com/photoreactors-leds-accessories/photoredox-box/?gad_source=1&gclid=Cj0KCQiAqL28BhCrARIsACYJvkeh6AQIqAcx_jsK5Zp6ga-0vP38EMEX8GraU8zTFJsSkIXnEF7ZYD0aAn2DEALw_wcB

) (pictured). PR160L lamps from Kessil (https://kessil.com/products/science_PR160L.php) are used in experiments. Emission spectra provided by Kessil are included below. The PhotoRedOx box is placed on a stirplate to provide stirring.

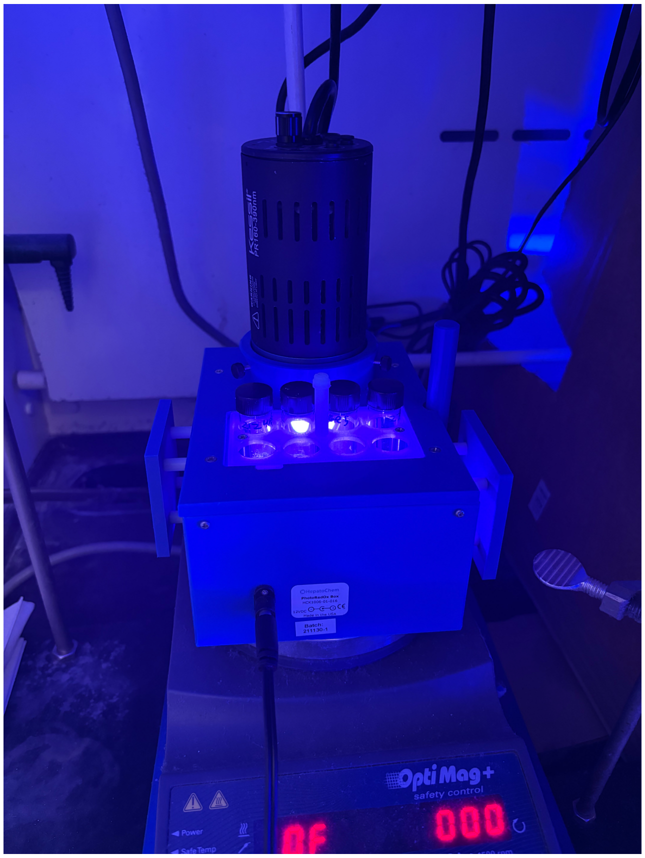

Pictured above: typical setup for photochemical reactions.

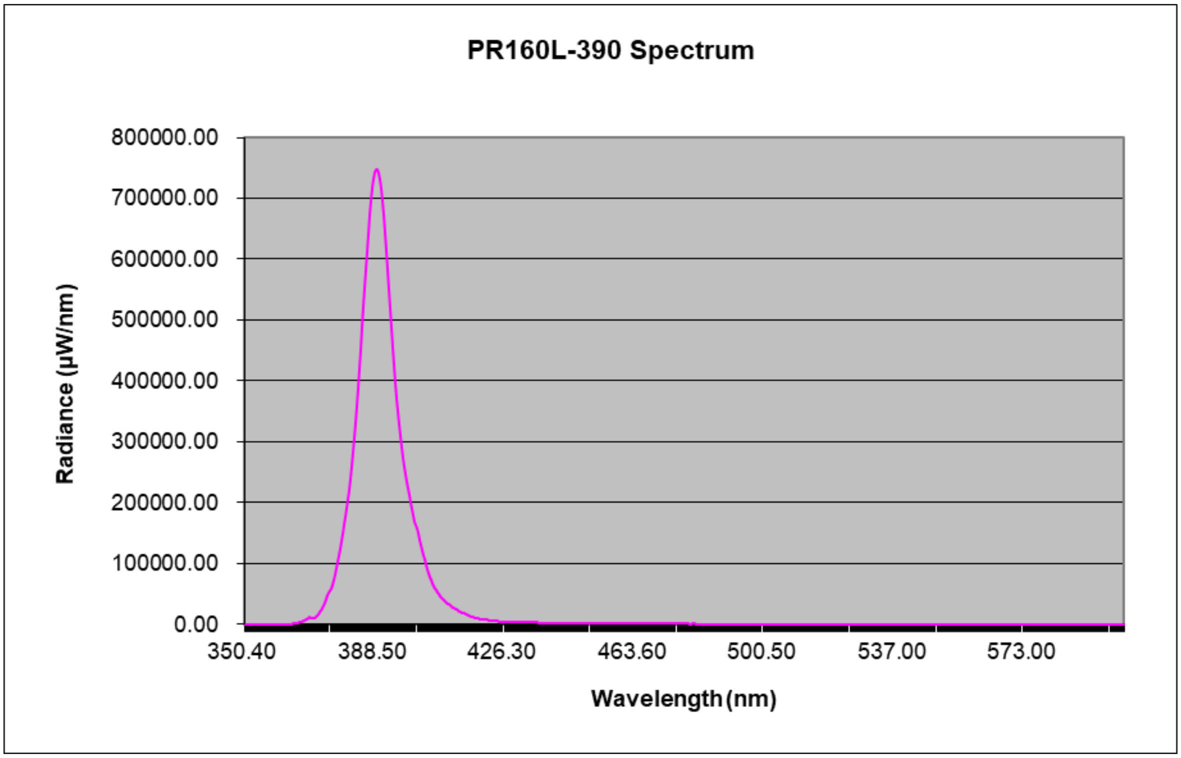

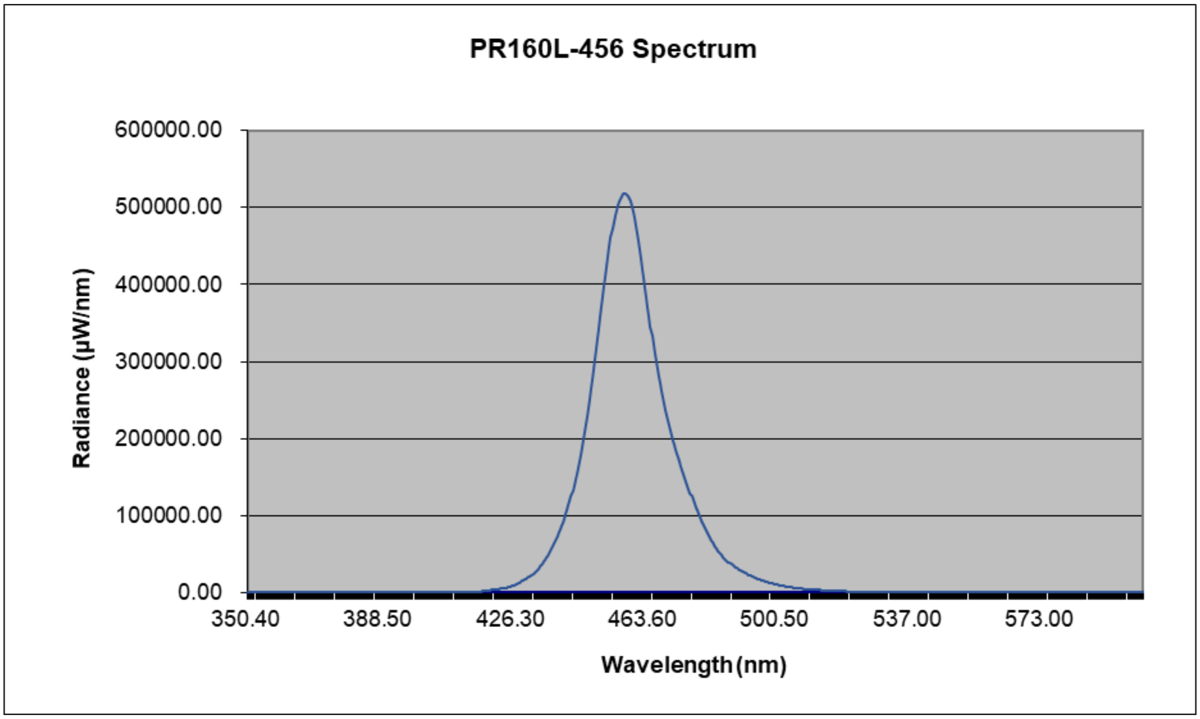

Data and graphs provided by Kessil. (https://kessil.com/products/science_PR160L.php)

Safety Statement

Caution! This procedure uses a 40 W LED for irradiation. Care should be taken to protect from exposure to heat and intense light. Tinted glasses are recommended when working with light sources.

A. General Procedures

I. General Procedure: photochemical reactions with TBA[$FeCl_3Br$]:

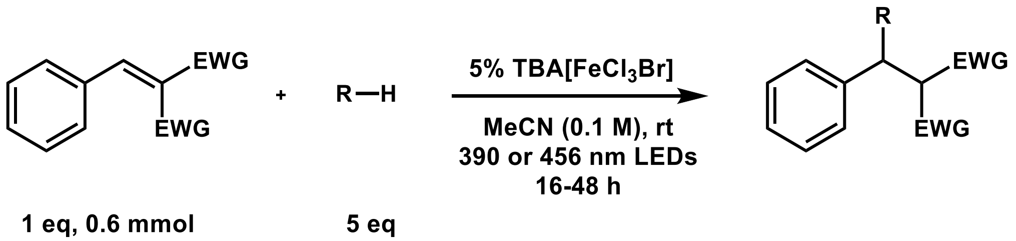

A typical procedure for a photoreaction with the tetrabutylammonium iron trichloride bromide salt can be conducted as follows: C-H Donor (3.0 mmol, 5.0 equiv.), TBA[$FeCl_3Br$] (10 mol%), radical acceptor (0.60 mmol, 1.0 equiv.), and dry acetonitrile (6.0 mL, 0.1M) were added to an 8-mL vial equipped with a super-micro stir bar. The vial was capped with the manufacturer provided cap and oxygen was not excluded. The vials were then stirred at room temperature for 16-48h under irradiation of either 390 nm or 456 nm LEDs. The reaction mixture was then concentrated *in vacuo* and the resulting residue was purified using column chromatography. All isolated products were further characterized by ^{1}H and $^{13}C\{^{1}H\}$ nuclear magnetic resonance (NMR) spectroscopy, infrared spectroscopy, and mass spectrometry.

II. Synthesis of radical acceptor benzylidene malononitrile derivatives:

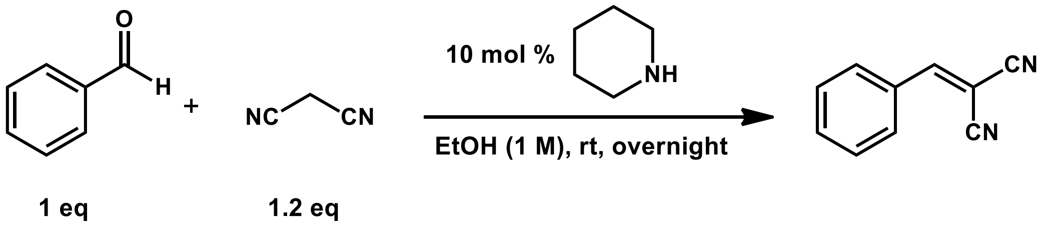

The synthesis of benzylidene malononitriles was adapted from known Knoevenagel condensation reactions. Malononitrile (24 mmol, 1.2 eq) and ethanol (20 mL, 1 M) were added to a round bottom flask charged with a magnetic stir bar. The aryl aldehyde (20 mmol, 1 eq) was then

added, followed by dropwise addition of piperidine (2 mmol, 0.1 eq). The mixture was stirred at room temperature overnight, or until precipitate caked the reaction. The mixture was then subjected to vacuum filtration to collect the precipitate, which was recrystallized with ethanol to afford a white solid.

<u>III. Synthesis of TBA[FeCl$_3$Br]:</u>

TBABr + $FeCl_3$ —EtOH (1 M)→ TBA[FeCl$_3$Br]

Anhydrous iron trichloride (3.0 mmol, 1 equiv.) and tetrabutyl ammonium bromide were added to a round bottom flask charged with a magnetic stir bar. Absolute ethanol (3 mL, 1 M) was then added and the reaction was stirred at room temperature for 3 hours. The reaction mixture was then concentrated in vacuo and used without further purification.

TBA[FeBr-$_4$] was synthesized according to the same procedure, but substituting iron tribromide (3.0 mmol, 1 equiv).

IV. Synthesis of Iron salen/salophen Catalysts:

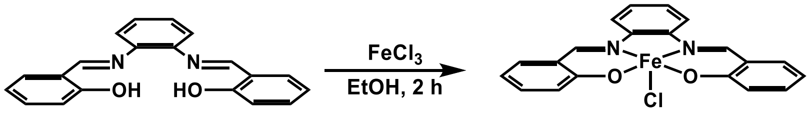

5

The procedures for the synthesis of iron chloride salen/salophen catalysts was adapted from previously reported procedures.[1] Salophen (5.0 mmol, 1 equiv.) and iron (III) trichloride (5.0 mmol, 1 equiv.) was added to a round bottom flask and dissolved in ethanol (0.02 M, 250 mL). The solution was refluxed under air for 2 hours and concentrated in vacuo. The final solid was filtered and washed with cold ethanol to afford the iron catalyst.

V. Iron salen/salophen results

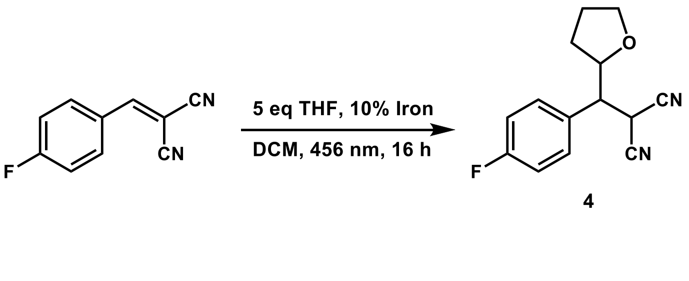

4

Table S1: Screening of iron salen/salophen catalysts

Entry	Catalyst	Prd 4[a]
**1**	Fe 1	**83%**
**2**	Fe 2	29%
**3**	Fe 3	7%
**4**	Fe 4	64%

[a]Yields determined by ^{1}H NMR

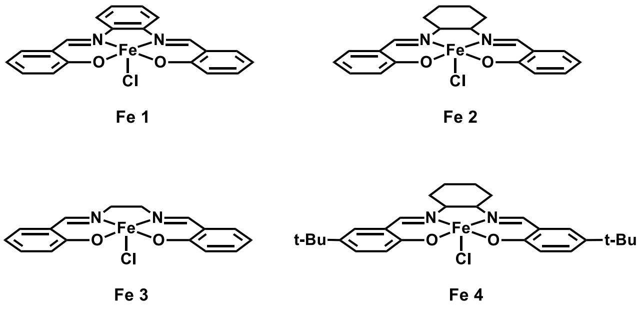

VI. UV-Vis Studies

General Procedures:

Samples were prepared using volumetric flasks. 3 mL of the sample was transferred to a quartz cuvette for analysis.

Sample Preparation: (0.26 mM $FeCl_3$ in MeCN)

$FeCl_3$ (26.2 mg, 0.162 mmol) was dissolved in 25 mL MeCN to create a 6.5 mM solution of $FeCl_3$. A serial dilution with volumetric flasks was performed to dilute to a 0.26 mM solution.

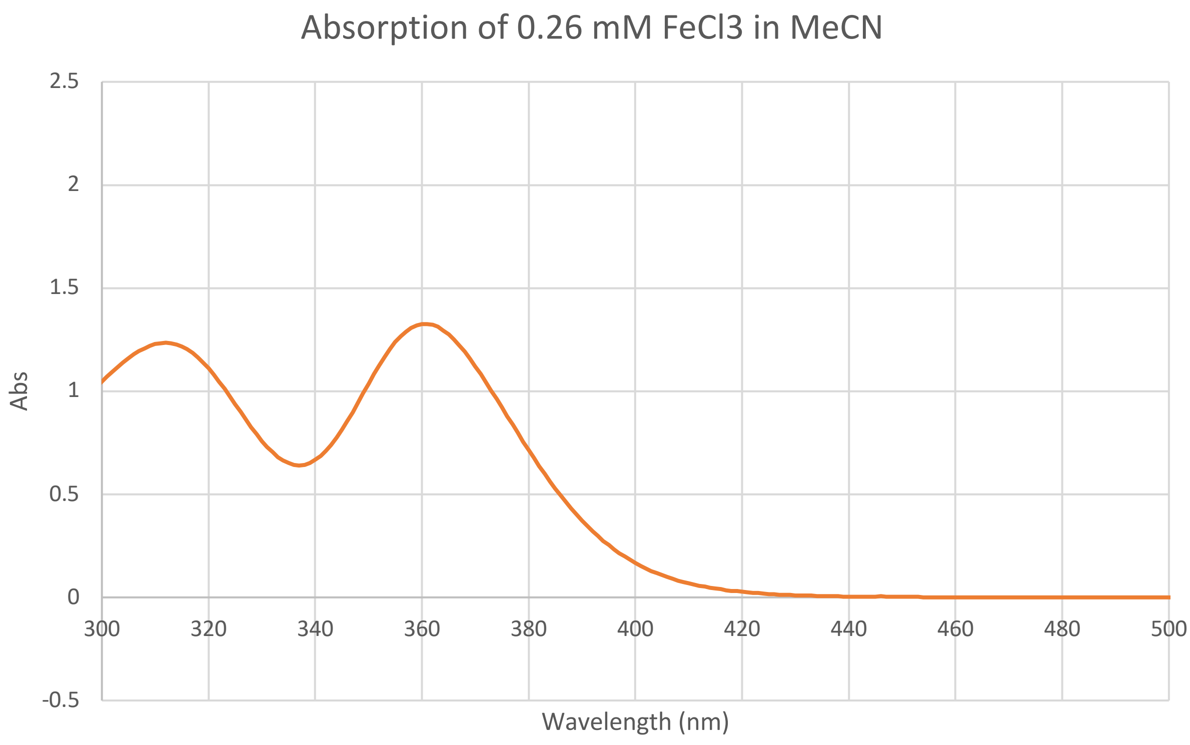

Figure S1: UV vis spectra of $FeCl_3$ in MeCN

Sample Preparation: (0.26 mM TBA[$FeCl_3Br$] in MeCN)

TBA[$FeCl_3Br$] (100 mg, 0.162 mmol) was dissolved in 25 mL MeCN to create a 6.5 mM solution of TBA[$FeCl_3Br$]. A serial dilution with volumetric flasks was performed to dilute to a 0.26 mM solution.

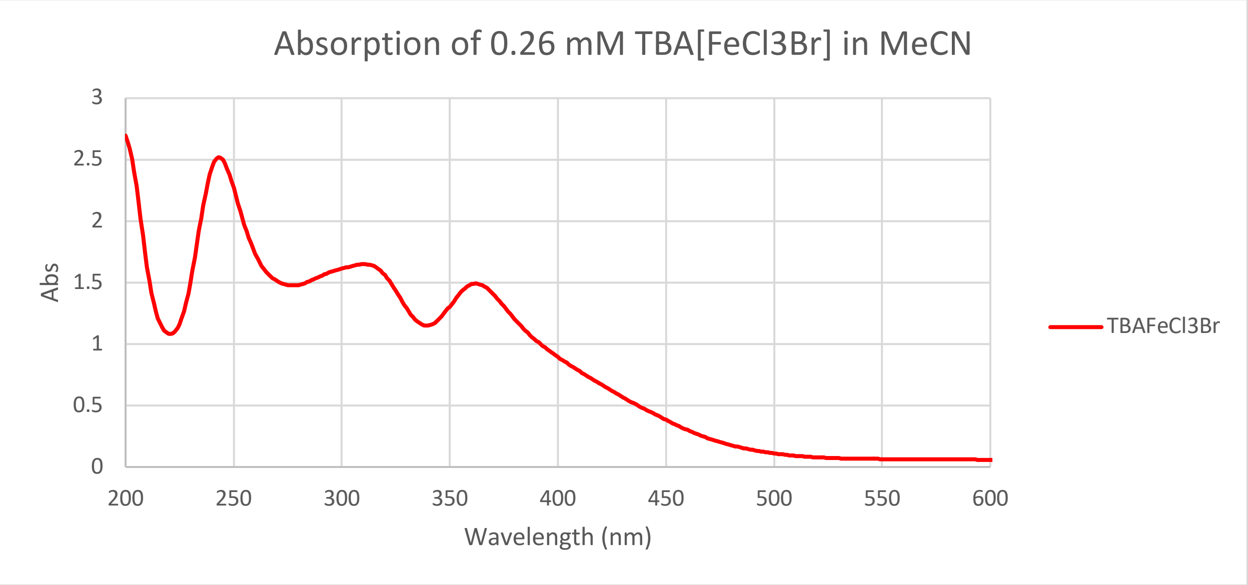

Figure S2: UV-Vis spectra of TBA[$FeCl_3Br$] in MeCN

B. Optimization Details

I. General Procedure for the optimization of photochemical reactions with TBA[$FeCl_3Br$]:

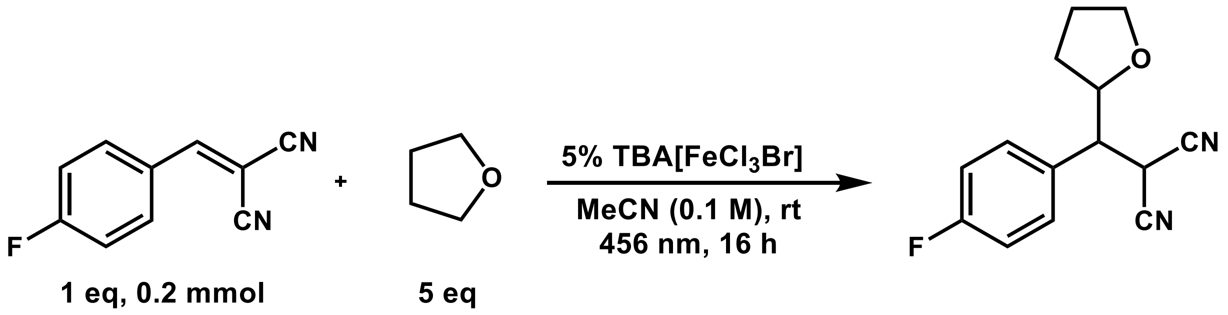

3 **2** **4**

Tetrahydrofuran (1.0 mmol, 5.0 equiv.), TBA[$FeCl_3Br$] (5 mol%), radical acceptor (0.2 mmol, 1.0 equiv.), and dry acetonitrile (2.0 mL, 0.1M) were added to an 8-mL vial equipped with a super-

micro stir bar. The vial was capped with the manufacturer provided cap and then stirred at room temperature for 16-48h under irradiation of either 390 nm or 456 nm LEDs.

B1. Addition of internal standard for GC assay:

Benzodioxle (0.20 mmol) was added to the crude reaction mixture, followed by mixing with a pipette. Approximately five drops of the resulting mixture was added to a GC vial and diluted with dichloromethane. A GC yield and a percentage of remaining starting material were calculated using peak areas of the internal standard, product, and starting material.

II. Optimization Results

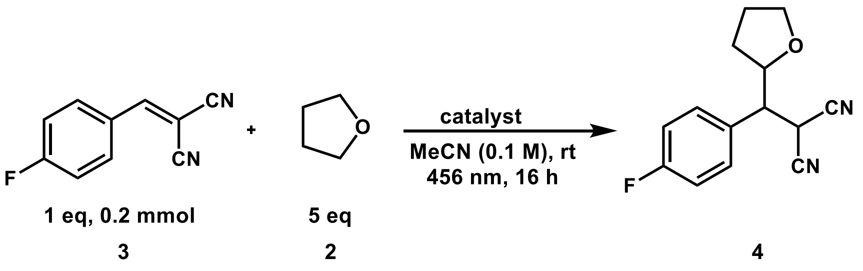

Table S2: Screening of iron catalyst optimization conditions.

Entry	Catalyst	Additive	Wavelength	Prd 4[a]
**1**	**5% TBA[FeCl$_3$Br]**	**None**	**456 nm**	**94%**
**2**	5% Li[FeCl$_3$Br]	None	456 nm	83%
**3**	5% TBA[FeCl$_3$Br]	5% LiBr	456 nm	69%
**4**	10% FeCl$_3$	None	456 nm	41%
**5**	10% FeCl$_3$	5% LiBr	456 nm	86%
**6**	10% FeCl$_3$	10% LiBr	456 nm	82%
**7**	10% FeCl$_3$	20% LiBr	456 nm	57%
**8**	10% FeCl$_3$	50% LiBr	456 nm	27%
**9**	10% FeCl$_3$	100% LiBr	456 nm	4%
**10**	10% FeCl$_3$	None	390 nm	80%
**11**	No Iron	None	456 nm	0%
**12**	5% TBA[FeCl$_3$Br]	None	No Light	0%

[a]Yields determined by ^{1}H NMR

C. Mechanistic Studies

I. Kinetic Isotope Effect Results

General Procedure for Kinetic Isotope Effect Experiments:

Tetrahydrofuran (1.0 mmol, 5.0 equiv.) or d_8-tetrohydrofuran (1.0 mmol, 5.0 equiv.) TBA[$FeCl_3$Br] (5 mol%), radical acceptor (0.2 mmol, 1.0 equiv.), and dry acetonitrile (2.0 mL, 0.1M) were added to an 8-mL vial equipped with a super-micro stir bar. The vial was capped with the manufacturer provided cap and then stirred at room temperature for 30 min - 3 hours under irradiation of either 390 nm or 456 nm LEDs. The solution was filtered through a silica plug followed by addition of benzodioxole (0.2 mmol, 1.0 eq) as internal standard. Resulting solution was added to a GC-FID for $FeCl_3$ parallel KIE experiments or analyzed by proton NMR for all other KIE experiments.

Parallel KIE for $FeCl_3$

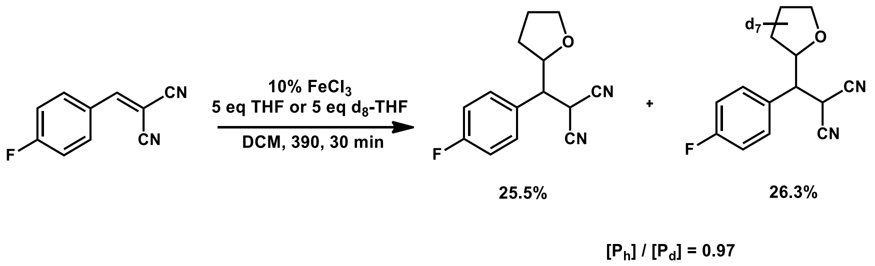

Parallel KIE's for TBA[$FeCl_3$Br]

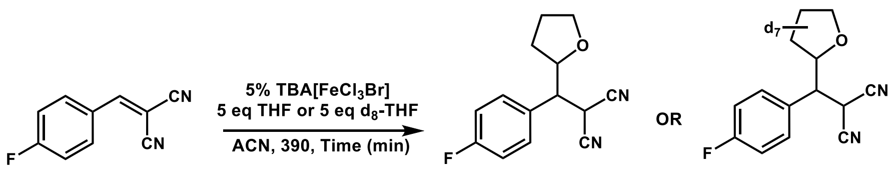

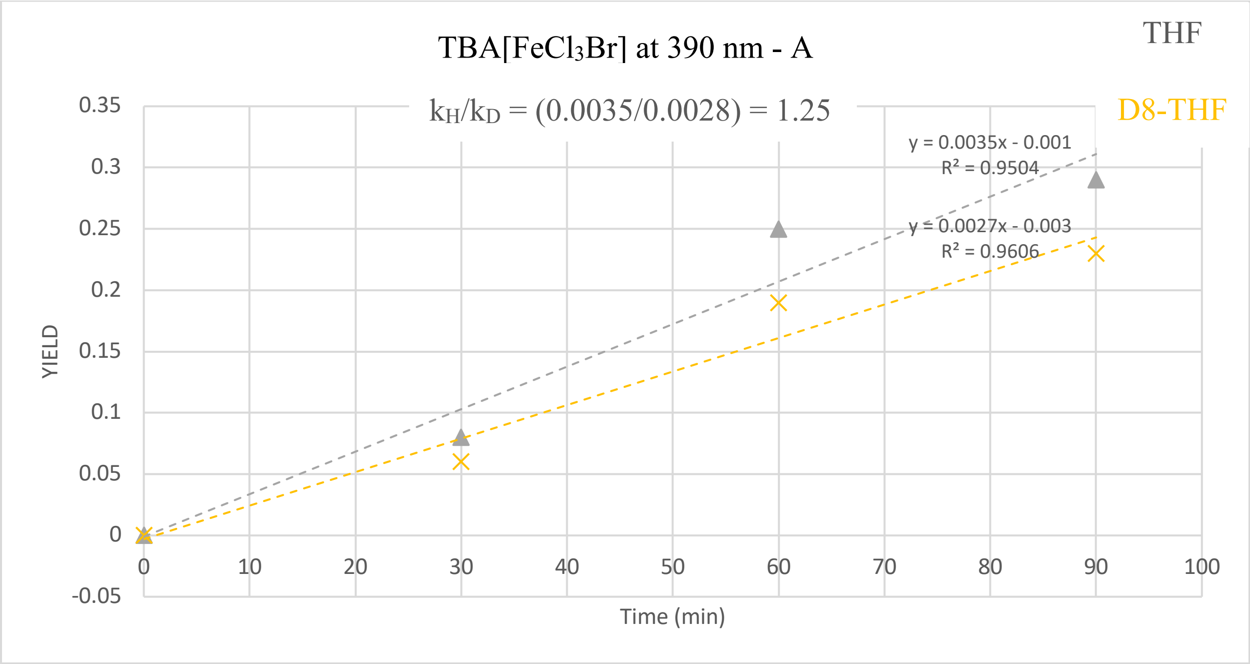
TBA[FeCl3Br] at 390 nm - A
THF
kH/kD = (0.0035/0.0028) = 1.25
D8-THF
y = 0.0035x - 0.001
R² = 0.9504
y = 0.0027x - 0.003
R² = 0.9606
YIELD
0.35
0.3
0.25
0.2
0.15
0.1
0.05
0
-0.05
0
10
20
30
40
50
60
70
80
90
100
Time (min)

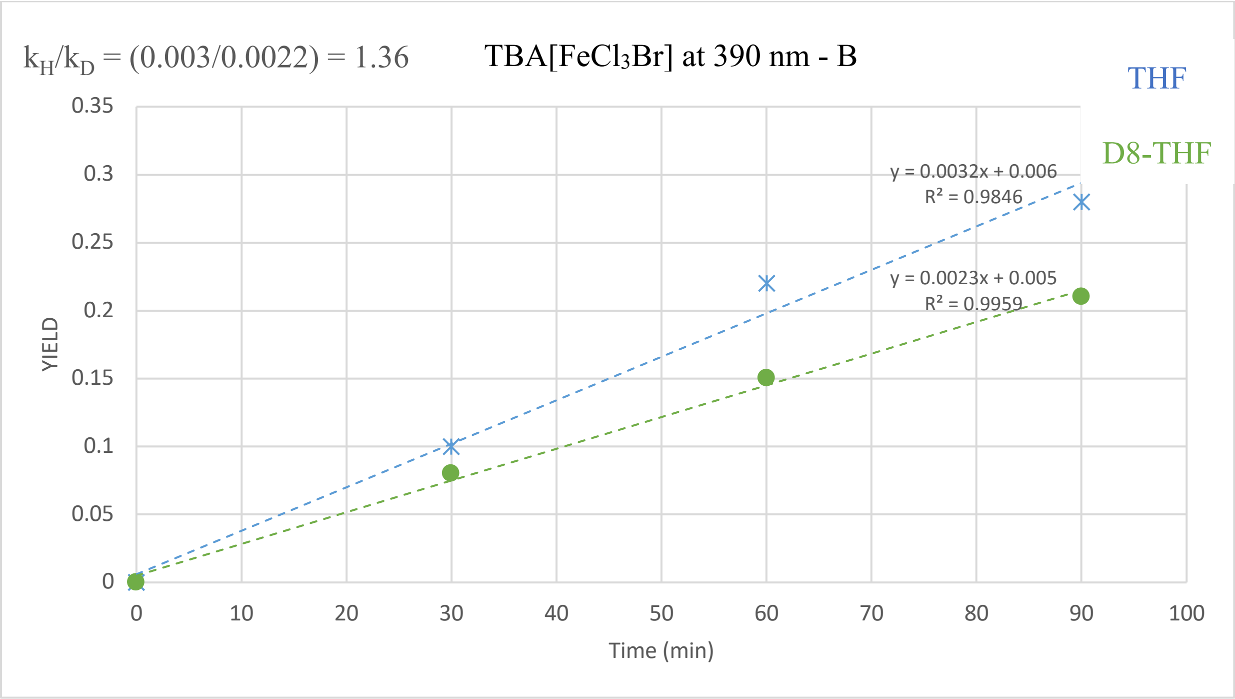
kH/kD = (0.003/0.0022) = 1.36
TBA[FeCl3Br] at 390 nm - B
THF
D8-THF
y = 0.0032x + 0.006
R² = 0.9846
y = 0.0023x + 0.005
R² = 0.9959
YIELD
0.35
0.3
0.25
0.2
0.15
0.1
0.05
0
0
10
20
30
40
50
60
70
80
90
100
Time (min)

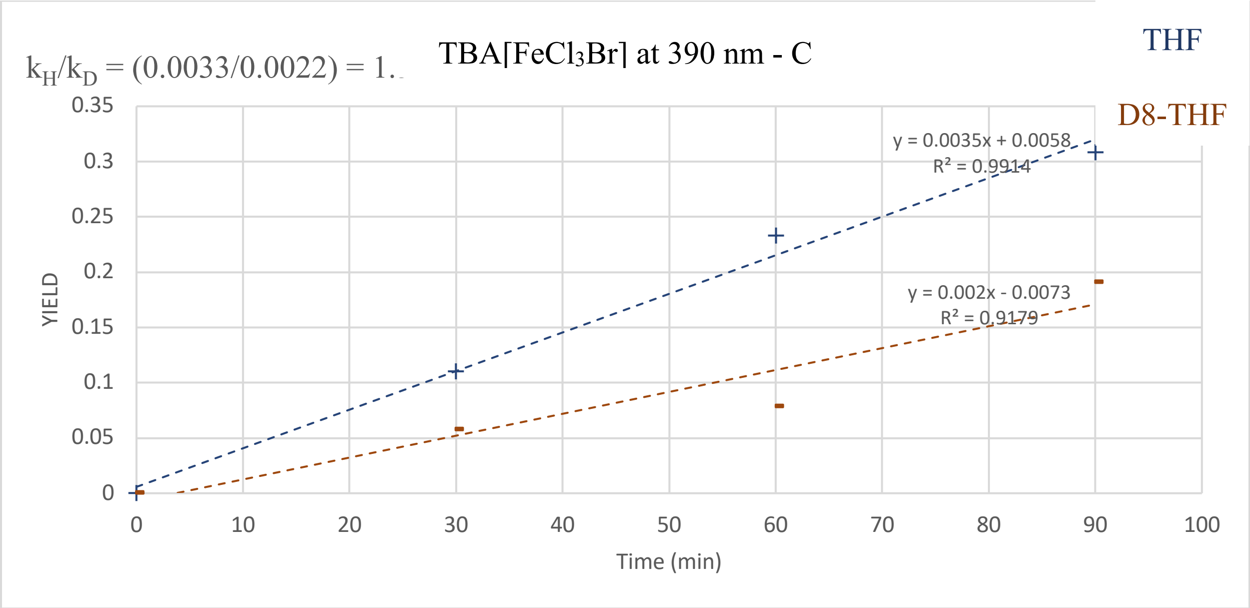

KIEs FOUND: 1.25, 1.36, 1.5. Average = 1.37 ± 0.152

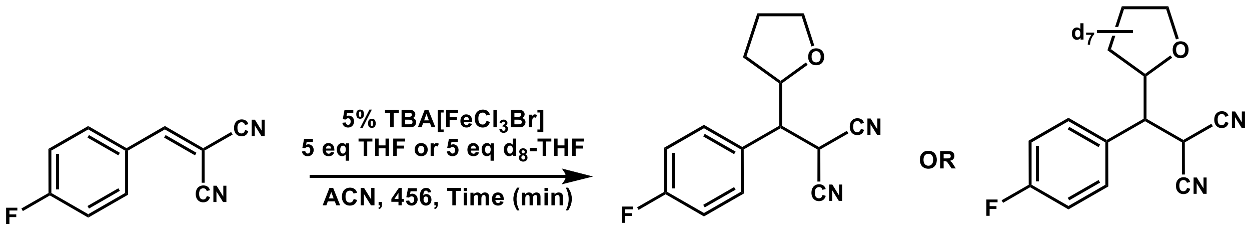

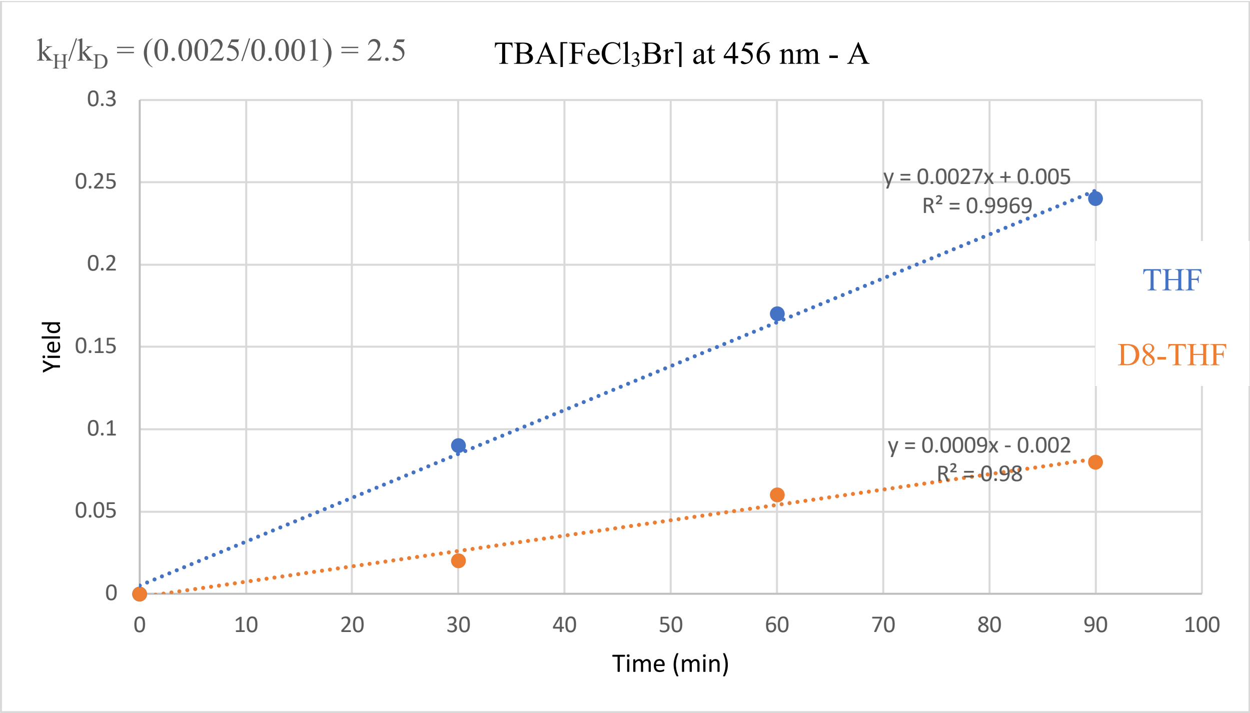

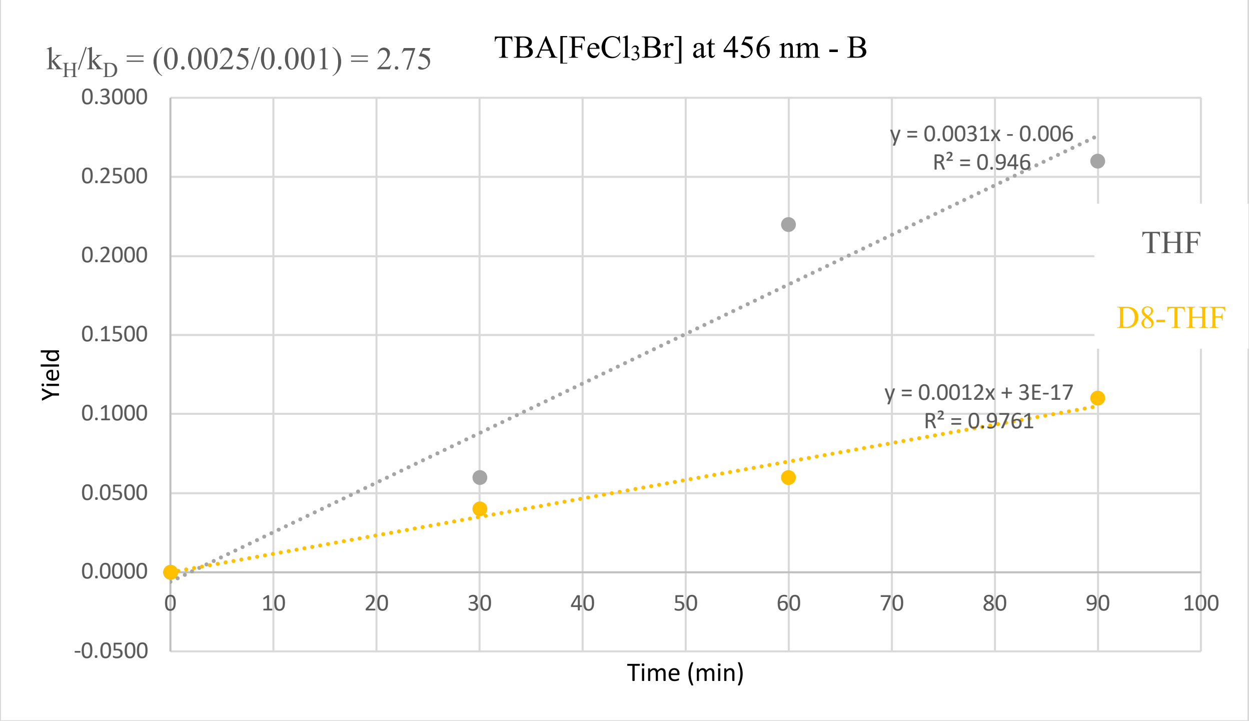

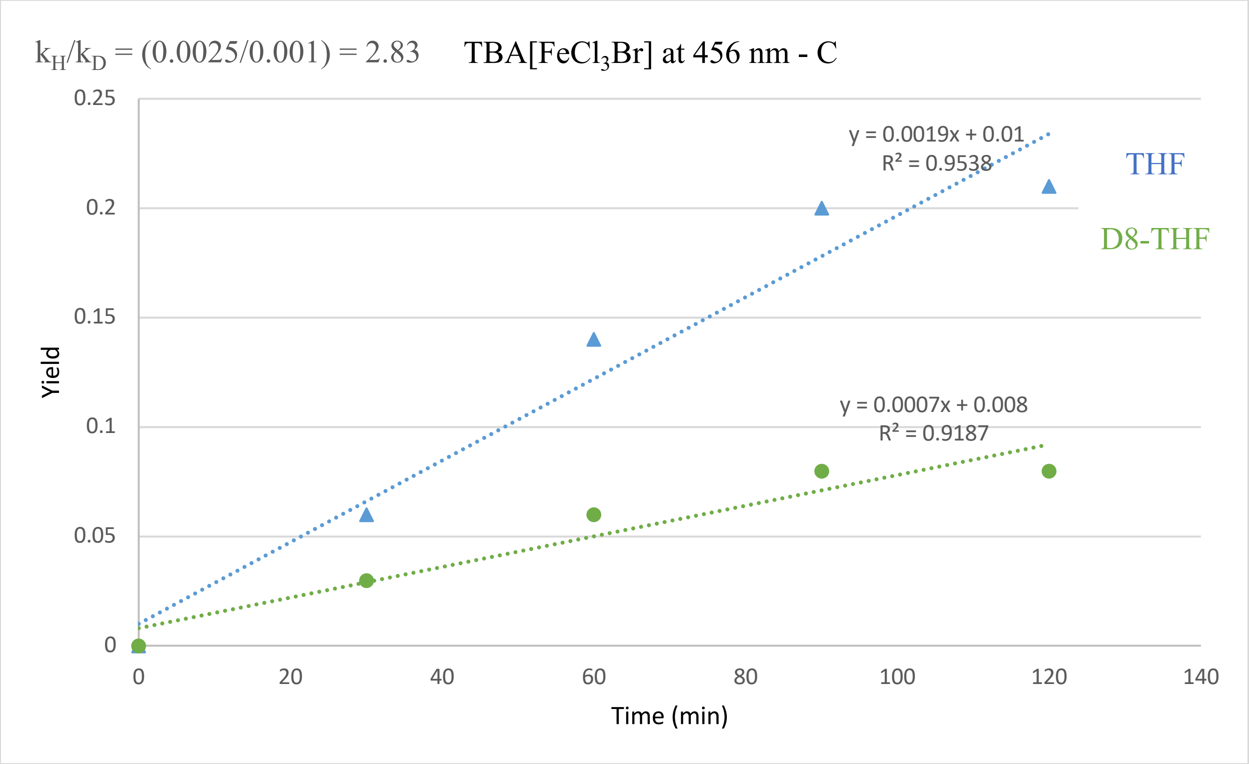

KIEs FOUND: 2.5, 2.75, 2.83 Average = 2.69 ± 0.267

Competition KIE for $FeCl_3$

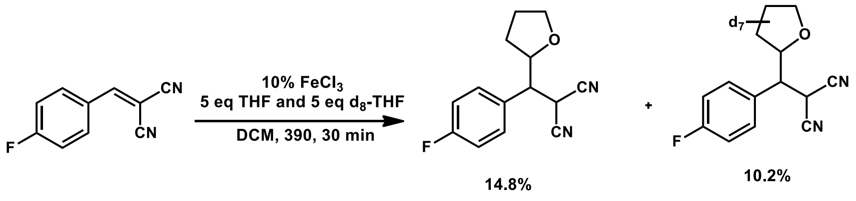

$[P_h] / [P_d] = 1.45$

Competition KIE for TBA[$FeCl_3$Br]

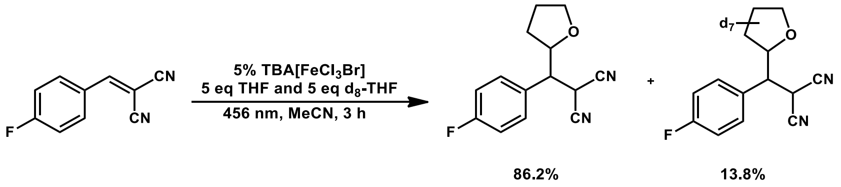

$[P_h] / [P_d] = 6.25$

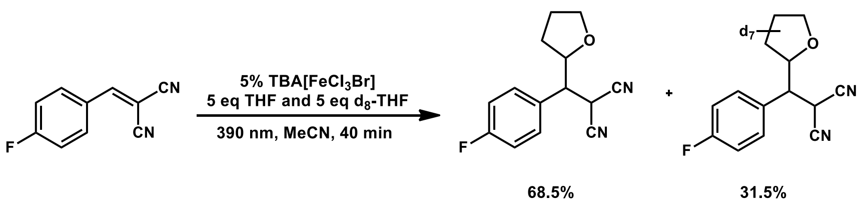

$[P_h] / [P_d] = 2.17$

II. Catalyst Selectivity Results

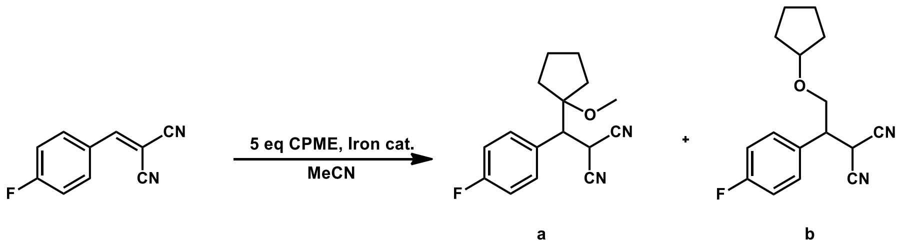

Table S3: CPME selectivity changes based on solvent.

Entry	Iron	Solvent	hv	a : b
**1**	10% $FeCl_3$	MeCN	390 nm	1 : 1.29
**2**	5% TBA[$FeCl_3$Br]	MeCN	390 nm	1 : 1.01
**3**	5% TBA[$FeCl_3$Br]	MeCN	456 nm	2.34 : 1
**4**	5% TBA[$FeCl_3$Br]	Cl-Ph	456 nm	2.6 : 1
**5**	5% TBA[$FeCl_3$Br]	Benzene	456 nm	2.9 : 1

[a]Yields determined by ^{1}H NMR

D. Characterization Data of Products

2-((4-fluorophenyl)(tetrahydrofuran-2-yl)methyl)malononitrile

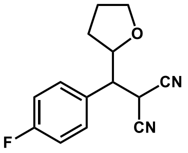

4

Made according to General procedure A at both 456 and 390 nm. Purified by column chromatography (10% EtOAc/Hexanes). Isolated as a pale yellow oil (124.1 mg, 85%) at 456 nm and (132.9 mg, 91%) at 390 nm.

A reaction run on 1.5 mmol scale was conducted as follows: THF (609 µL, 7.5 mmol, 5.0 equiv.), TBA[$FeCl_3Br$] (36.3 mg, 0.075 mmol, 5 mol%), radical acceptor (258.3 mg, 1.50 mmol, 1.0 equiv.), and dry acetonitrile (15.0 mL, 0.1 M) were added to a 20-mL scintillation vial equipped with a micro stir bar. The vial was capped with the manufacturer provided cap and oxygen was not excluded. The vial was then placed in a Hepatochem PhotoRedOx Box and stirred at room temperature for 144 h under irradiation of 456 nm LEDs (one 40 W Kessil lamp). The reaction mixture was then concentrated *in vacuo* and the resulting residue was purified using column chromatography(10% EtOAc/Hexanes) to give the desired product in 93% isolated yield (339.4 mg).

^{1}H NMR (400 MHz, $CDCl_3$) δ 7.41 – 7.27 (m, 4H), 7.09 (d, *J* = 15.5 Hz, 4H), 4.53 (d, *J* = 4.1 Hz, 1H), 4.43 (d, *J* = 3.2 Hz, 1H), 4.32 (d, *J* = 10.5 Hz, 1H), 3.99 – 3.81 (m, 1H), 3.74 (td, *J* = 6.7, 1.5 Hz, 3H), 3.28 (dd, *J* = 10.6, 3.1 Hz, 1H), 3.03 (dd, *J* = 10.3, 4.1 Hz, 2H), 1.91 (s, 1H), 1.53 – 1.29 (m, 2H).

^{13}C{^{1}H} NMR (101 MHz, $CDCl_3$) δ 163.1 (d, *J* = 248.7 Hz), 163.0 (d, *J* = 249.7 Hz), 131.1, 130.3 (d, *J* = 8.2 Hz), 129.8 (d, *J* = 3.4 Hz), 116.4 (d, *J* = 21.7 Hz), 116.1 (d, *J* = 21.6 Hz), 112.2 (d, *J* = 8.8 Hz), 111.6, 78.0, 68.8, 50.6 (d, *J* = 145.0 Hz), 30.4, 28.9, 28.5 – 26.3 (m), 25.8.

LC-HRMS (ESI-Orbitrap) m/z: Calcd for $C_{14}H_{12}FN_2O$ [M-H]$^-$ 243.0939, found 243.0938

IR: 2917, 1605, 1510, 1228, 1065, 837

2-(1-(4-fluorophenyl)2-phenylethyl)malononitrile

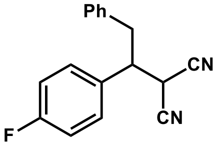

10

Made according to General procedure A at both 456 and 390 nm. Purified by column chromatography (20% EtOAc/Hex). Isolated as a pale yellow oil, 86% at 456 nm and 71% at 390 nm.

^{1}H NMR (400 MHz, $CDCl_3$) δ 7.47 – 7.23 (m, 5H), 7.21 – 7.02 (m, 4H), 3.82 (d, *J* = 5.0 Hz, 1H), 3.45 (d, *J* = 5.1 Hz, 1H), 3.32 – 3.13 (m, 2H).

$^{13}C\{^1H\}$ NMR (101 MHz, $CDCl_3$) δ 163.01 (d, *J* = 248.7 Hz), 136.37, 132.16, 130.71 – 125.02 (m), 116.27 (d, *J* = 21.7 Hz), 111.59 (d, *J* = 68.5 Hz), 47.68, 38.61, 28.58. Known compound reported in literature, spectrum matches known spectra.[2]

2-(1-(4-fluorophenyl)-2-oxo-2-phenylethyl)malononitrile

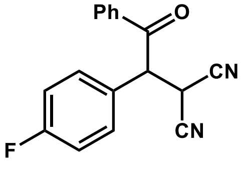

11

Made according to General procedure A at both 456 and 390 nm. Purified by column chromatography (10% EtOAc/Hexanes). Isolated as a pale yellow oil (101.5 mg, 61%) at 456 nm over 36 h and (151.4 mg, 91%) at 390 nm.

^{1}H NMR (400 MHz, $CDCl_3$) δ 7.87 (d, *J* = 7.7 Hz, 2H), 7.61 – 7.51 (m, 1H), 7.42 (t, *J* = 7.7 Hz, 2H), 7.34 (dd, *J* = 8.5, 5.0 Hz, 2H), 7.11 (t, *J* = 8.5 Hz, 2H), 5.10 (d, *J* = 8.2 Hz, 1H), 4.51 (d, *J* = 8.2 Hz, 1H).

$^{13}C\{^1H\}$ NMR (126 MHz, $CDCl_3$) δ 192.9, 163.4 (d, *J* = 251.0 Hz), 134.6, 133.7, 130.6 (d, *J* = 8.7 Hz), 129.2 (d, *J* = 18.9 Hz), 127.9 (d, *J* = 3.5 Hz), 117.3 (d, *J* = 22.1 Hz), 111.7 (d, *J* = 53.7 Hz), 54.0, 29.7, 26.8.

IR, film (neat, cm-1): 3064, 3030, 2903, 2863, 2255, 2230, 1955, 1894, 1682, 1605, 1512, 1497, 1455, 1231, 1162, 841

LC-HRMS (ESI-Orbitrap) m/z: Calcd for $C_{17}H_{10}FN_2O$ $[M-H]^-$ 277.0783, found 277.0782

2-amino-4-(4-fluorophenyl)-5,5-dimethyl-4,5-dihydrofuran-3-carbonitrile

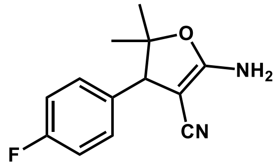

12

Made according to General procedure A at both 456 and 390 nm. Purified by column chromatography (10% EtOAc/Hexanes). Isolated as a white solid (112.9 mg, 81%) at 456 nm and (128.2 mg, 92%) at 390 nm.

^{1}H NMR (400 MHz, $CDCl_3$) δ 7.15 (dd, *J* = 8.6, 5.4 Hz, 2H), 7.03 (t, *J* = 8.6 Hz, 2H), 4.83 (s, 2H), 4.01 (s, 1H), 1.54 (s, 3H), 0.86 (s, 3H).

GC-HRMS (EI-TOF) m/z: Calcd for $C_{13}H_{13}FN_2O$ $[M]^+$ 232.1006, found 232.0989

Known compound reported in literature, spectrum matches known spectra.[3]

2-((1,4-dioxan-2-yl)(4-fluorophenyl)methyl)malononitrile

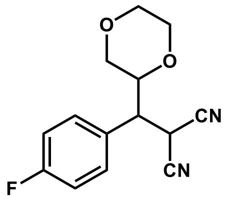

13

Made according to General procedure. Purified by column chromatography (15% EtOAc/Hexanes). Isolated as a white solid (126.0 mg, 81%) at 390 nm

^{1}H NMR (400 MHz, $CDCl_3$) δ 7.44 – 7.30 (m, 2H), 7.21 – 7.03 (m, 2H), 4.65 – 4.26 (m, 1H), 4.21 – 4.00 (m, 1H), 3.99 – 3.67 (m, 3H), 3.67 – 3.39 (m, 2H), 3.35 – 3.04 (m, 1H), 2.96 (dd, *J* = 11.6, 10.0 Hz, 1H).

^{13}C{^{1}H} NMR (126 MHz, $CDCl_3$) δ 162.7 (d, *J* = 249.5 Hz), 162.4 (d, *J* = 249.0 Hz), 130.7 (d, *J* = 7.6 Hz), 129.9 (d, *J* = 8.0 Hz), 128.6, 127.8, 116.2 (d, *J* = 21.7 Hz), 115.8 (d, *J* = 21.4 Hz), 111.31, 111.29, 111.1, 110.8, 73.12, 73.05, 68.8, 68.6, 67.5, 66.9, 65.92, 65.90, 47.23, 47.16, 26.6, 26.3.

IR, film (neat, cm-1): 2969, 2915, 2862, 1605, 1511, 1229, 1119, 910, 728

LC-HRMS (ESI-Orbitrap) m/z: Calcd for $C_{14}H_{12}FN_2O_2^-$ [M-H]$^-$ 259.0888, found 259.0888

2-(benzo[d][1,3]dioxol-2-yl(4-fluorophenyl)methyl)malononitrile

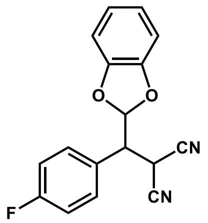

14

Yield = Made according to General procedure A. Purified by column chromatography (5% EtOAc/Hexanes to 10% EtOAc/Hexanes). Isolated as a white solid (102.3 mg, 58%) at 390 nm

^{1}H NMR (500 MHz, $CDCl_3$) δ 7.55 – 7.47 (m, 2H), 7.17 (t, *J* = 8.6 Hz, 2H), 6.89 (d, *J* = 3.0 Hz, 3H), 6.87 – 6.82 (m, 1H), 6.48 (d, *J* = 4.3 Hz, 1H), 4.40 (d, *J* = 5.8 Hz, 1H), 3.75 (dd, *J* = 5.8, 4.3 Hz, 1H).

^{13}C{^{1}H} NMR (126 MHz, $CDCl_3$) δ 163.5 (d, *J* = 250.1 Hz), 146.3 (d, *J* = 19.9 Hz), 131.0 (d, *J* = 8.6 Hz), 127.2 (d, *J* = 3.4 Hz), 122.7 (d, *J* = 6.8 Hz), 116.7 (d, *J* = 21.8 Hz), 111.0 (d, *J* = 31.2 Hz), 109.3 (d, *J* = 11.4 Hz), 108.6, 49.2, 24.6.

IR, film (neat, cm-1): 2901, 1609, 1515, 1479, 1232, 851, 744

GC-HRMS (EI-TOF) m/z: Calcd for $C_{17}H_{11}FN_2O_2$ [M]$^+$ 294.0804, found 294.0807

2-(1-(4-fluorophenyl)-3,4-dimethylpent-3-en-1-yl)malononitrile

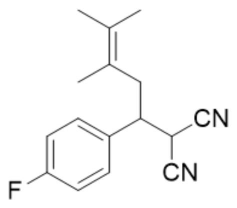

15

Yield = 74% at 456 nm, 87% at 390 nm.

According to General Procedure A, 4-fluoromethylbenzylidene malononitrile (0.60 mmol, 103.3 mg 1.0 equiv), catalyst TBAFeCl_3Br (15 mol%), and 2,3 dimethyl -2-butene (1.8 mmol, 3.0 equiv), (223 µl) in CH_3CN (3.0 mL) were reacted for 72 hours under N_2. The crude residue was purified by column chromatography on silica gel (3-10% EtOAc/Hexanes) to afford **15 (**113.7 mg, 74 % yield) as a clear oil.

^{1}H NMR (500 MHz, $CDCl_3$) δ 7.39 (d, J = 8.2 Hz, 2H), 7.12 (d, J = 8.2 Hz, 2H), 3.97 (d, J = 5.0 Hz, 1H), 3.42 (ddd, J= 8.9, 6.6, 5.1 Hz, 1H), 2.94 (dd, J = 14.0, 8.9 Hz, 1H), 2.53 (dd, J = 14.0, 6.6 Hz, 1H), 1.72 (s,3H), 1.66 (s, 3H), 1.62 (s, 3H);

^{13}C{^{1}H} NMR (126 MHz, $CDCl_3$) δ 162.9, (d, J = 249.1 Hz), 132.8, (d, J = 2.5 Hz), 130.4, 129.7, 122.4, 116.1 (d, J = 21.4 Hz), 112.3, 111.7, 44.8, 37.3, 28.6, 21.0, 20.7, 18.3

GC-HRMS (EI-TOF) m/z: Calcd for $C_{16}H_{17}FN_2$ [M+] = 256.1376, found 256.1379

2-(cyclohex-2-en-1-yl(4-fluorophenyl)methyl)malononitrile

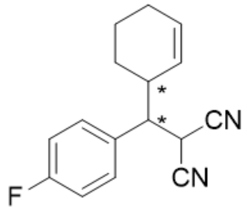

16

Yield = 65% at 456 nm, 83% at 390 nm.

According to General Procedure A, 4-fluoromethylbenzylidene malononitrile (0.60 mmol, 103.2 mg 1.0 equiv), catalyst TBAFeCl_3Br (15 mol%), and cyclohexene (1.8 mmol, 3.0 equiv), (185 µl)

in CH_3CN (3.0 mL) were reacted for 72 hours under N_2. The crude residue was purified by column chromatography on silica gel (3-10% EtOAc/Hexanes) to afford **16** as separable mixture of diastereomers (50/50 ratio, 99.1 mg total, 65 % yield) as clear oils.

Diastereomer 1:

^{1}H NMR (500 MHz, $CDCl_3$) δ 7.40-7.32 (dd, *J* = 8.6, Hz, 2H), 7.14-7.10 (t, *J* = 8.6 Hz, 2H), 6.03-5.93 (m, 1H), 5.78-5.73 (m, 1H), 4.25 (d, J = 5.4 Hz, 1H), 3.08-3.03 (dd, *J* = 9.5 Hz, 1H), 2.79-2.73 (m, 1H), 2.06-2.01 (m, 2H), 1.6-1.5 (m 3H), 1.2.8-1.19 (m, 1H).

^{13}C{^{1}H} 13C NMR (126 MHz, $CDCl_3$) δ 163.0 (d, *J* = 249.1Hz), 132.0 (d, *J* = 3.4 Hz), 130.5, 130.3 (d, *J* = 8.8 Hz), 126.0, 116.3 (d, *J* = 21.4 Hz), 111.8, 111.4, 50.7, 37.1, 27.7, 27.5 (d, *J* = 1.3 Hz), 24.8, 21.1.

GC-HRMS (EI-TOF) m/z: Calcd for $C_{16}H_{15}FN_2$ (M+) = 254.1215, found 254.1219

Diastereomer 2:

^{1}H NMR (500 MHz, $CDCl_3$) δ 7.38-7.34 (dd, *J* = 8.6, Hz, 2H), 7.14-7.10 (t, *J* = 8.6 Hz, 2H), 5.77-5.72 (m, 1H), 5.21-5.16 (m, 1H), 4.18-4.16 (d, *J* = 5.4 Hz, 1H), 2.96-2.92 (dd, *J* = 9.5 Hz, 1H), 2.90-2.83 (m, 1H), 2.07-1.95 (m, 2H), 1.86-1.80 (m 1H), 1.68 –1.59 (m, 2H), 1.38-1.30(m 1 H).

^{13}C{^{1}H} 13C NMR (126 MHz, $CDCl_3$) δ 162.9, (d, *J* = 249.1Hz), 132.7, 132.1(d, *J* = 3.8 Hz),130.0, (d, *J* = 8.8 Hz) 125.2, 116.2, (d, *J* = 21.4 Hz), 112.0, 111.6, 50.6, 36.0, 27.6 (d, *J* = 1.3 Hz), 25.7, 25.1, 19.2.

GC-HRMS (EI-TOF) m/z: Calcd for $C_{16}H_{15}FN_2$ (M+) = 255.1215, found 254.1219

2-(cyclohexyl(4-fluorophenyl)methyl)malononitrile

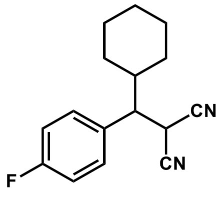

17

Made according to General procedure. Purified by column chromatography (10% EtOAc/Hexanes). Isolated as a pale yellow oil (110 mg, 72%) at 390 nm.

^{1}H NMR (400 MHz, $CDCl_3$) δ 7.31 (dd, *J* = 8.6, 5.3 Hz, 2H), 7.10 (t, *J* = 8.6 Hz, 2H), 4.18 (d, *J* = 5.3 Hz, 1H), 2.87 (dd, *J* = 9.9, 5.3 Hz, 1H), 2.08 – 1.80 (m, 3H), 1.67 (tt, *J* = 9.6, 2.6 Hz, 2H), 1.52 – 1.32 (m, 2H), 1.29 – 1.05 (m, 2H), 1.03 – 0.70 (m, 2H).

Known compound reported in literature, spectrum matches known spectra.[4]

2-(((3r,5r,7r)-Adamantan-1-yl)(phenyl)methyl) malononitrile

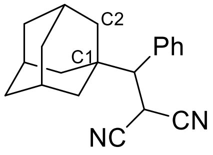

18

Mixture of two regioisomers

Yield = Made according to General procedure A, but time was extended to 60 hours. Purified by column chromatography (2% EtOAc/Hexanes to 10% EtOAc/Hexanes). Any post column fractions contaminated with starting alkylidene were treated with 0.10 mmol triethyl phosphite and 20 mol% phosphomolybdic acid, stirred for 90 min, concentrated, and filtered through a silica plug. Isolated as a white solid (136.6 mg, 47%) at 390 nm.

^{1}H NMR (400 MHz, $CDCl_3$) δ 7.44-7.34 (m, 8H), 4.24 (d, *J* = 5.4 Hz, 0.6H), 4.10 (d, *J* = 4.3 Hz, 1H), 3.49 (dd, *J* = 4.3 Hz, *J* = 11.9, 1H), 2.80 (d, *J* = 5.4 Hz, 1H), 2.38 (d, *J* = 11.9 Hz, 1H), 2.00 (m, 4H), 1.90 (m, 4H), 1.71 (M, 11.6H), 1.59 (m, 2H), 1.44 (m, 2H).

Known compound reported in literature, spectrum matches known spectra.[4]

2-(phenyl(tetrahydrofuran-2-yl)methyl)malononitrile

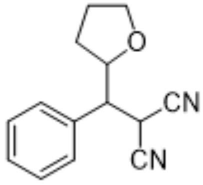

19

Made according to General procedure. Purified by column chromatography (5% EtOAc/Hexanes to 30% EtOAc/Hexanes). Isolated as white solid (123.3 mg, 91%) at 456 nm and (114.1 mg, 84%) at 390 nm, mixture of diasteromers (1:1.1)

^{1}H NMR (400 MHz, $CDCl_3$) δ7.41-7.37 (m, 8H), 7.33-7.29 (m, 2H), 4.55 (d, *J* = 4.1 Hz, 1H), 4.47-4.44 (m, 1H), 4.39 (m, 2H), 3.95 (m, 1H), 3.88 (m, 1H), 3.75 (m, 1H), 3.28 (dd, *J* = 3.2 Hz, *J* = 7.5 Hz, 1H), 3.04 (dd, *J* = 4.2 Hz, *J* = 6.1 Hz, 1H), 1.99-1.85 (m, 4H), 1.71-1.59 (m, 1H), 1.51-1.28 (m, 3H).

Known compound reported in literature, spectrum matches known spectra.[21]

2-((tetrahydro-2-furanyl)(4-(trifluoromethyl)phenyl)methyl)propanedinitrile

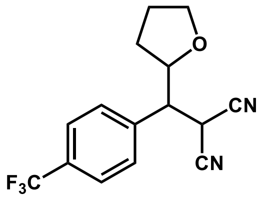

20

Made according to General procedure. Purified by column chromatography (10% EtOAc/Hexanes). Isolated as a yellow solid (151.3 mg, 86%) at 456 nm and (160.3 mg, 91%) at 390 nm.

^{1}H NMR (400 MHz, $CDCl_3$) δ 7.68 (dd, *J* = 16.3, 8.1 Hz, 2H), 7.50 (dd, *J* = 21.9, 8.1 Hz, 2H), 4.53 (dd, *J* = 50.1, 3.7 Hz, 1H), 4.39 (d, *J* = 10.5 Hz, 1H), 4.05 – 3.70 (m, 2H), 3.24 (ddd, *J* = 104.5, 10.4, 3.7 Hz, 1H), 2.07 – 1.74 (m, 2H), 1.62 – 1.27 (m, 2H).

Known compound reported in literature, spectrum matches known spectra.[5]

2-((tetrahydrofuran-2-yl)(thiophen-3-yl)methyl)malononitrile

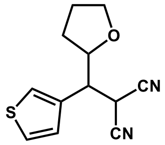

21

Made according to General procedure A. Purified by column chromatography (10% EtOAc/Hexanes). Isolated as a white solid (117.1 mg, 84%) at 456 nm and (132.4 mg, 95%) at 390 nm.

^{1}H NMR (400 MHz, $CDCl_3$) δ 7.43 – 7.33 (m, 3H), 7.30 (dd, *J* = 3.0, 1.4 Hz, 1H), 7.16 (dd, *J* = 5.0, 1.5 Hz, 1H), 7.07 (dd, *J* = 5.0, 1.4 Hz, 1H), 4.50 (d, *J* = 3.8 Hz, 1H), 4.40 (td, *J* = 7.2, 2.9 Hz, 1H), 4.35 – 4.25 (m, 2H), 3.98 – 3.70 (m, 4H), 3.48 (dd, *J* = 10.2, 2.9 Hz, 1H), 3.22 (dd, *J* = 10.4, 3.8 Hz, 1H), 2.03 – 1.88 (m, 4H), 1.87 – 1.73 (m, 1H), 1.56 – 1.33 (m, 3H).

^{13}C{^{1}H} NMR (101 MHz, $CDCl_3$) δ 134.7, 134.1, 127.6, 127.2, 126.9, 126.7, 125.4, 124.6, 112.5, 112.30, 112.26, 111.8, 78.1, 69.0, 68.8, 47.8, 46.3, 30.4, 29.0, 27.4, 27.3, 25.9, 25.8. (One peak obscured by $CDCl_3$ peak).

IR, film (neat, cm-1): 3105, 2874, 2361, 2254, 2026, 1725, 1076, 794

LC-HRMS (ESI-Orbitrap) m/z: Calcd for $C_{12}H_{13}N_2OS^+$ $[M+H]^+$ 233.0743, found 233.0742

1-phenyl-3-(tetrahydrofuran-2-yl)pyrrolidine-2,5-dione

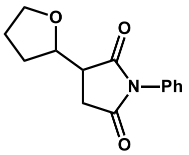

22

Made according to General procedure A. Purified by column chromatography (10% EtOAc/Hexanes). Isolated as a white solid (104.5 mg, mg, 71%) at 456 nm and (125.1 mg, 85%) at 390 nm.

^{1}H NMR ^{1}H NMR (400 MHz, $CDCl_3$) δ 7.45 (s, 1H), 7.38 (s, 1H), 7.31 (s, 1H), 4.40 (s, 1H), 4.28 (s, 0H), 3.96 – 3.72 (m, 2H), 3.29 (d, J = 9.3 Hz, 2H), 3.13 (s, 0H), 2.96 (s, 0H), 2.85 (s, 2H), 2.21 – 1.87 (m, 2H), 1.68 (d, J = 12.2 Hz, 1H).

Known compound reported in literature, spectrum matches known spectra.[6]

4-oxo-4-phenylbutanenitrile

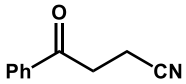

23

Made according to General procedure A but stirred for 36 hours. Purified by column chromatography (10% EtOAc/Hexanes). Isolated as a pale yellow oil (71.6 mg, mg, 75%) at 456 nm and (86.9 mg, 91%) at 390 nm.

^{1}H NMR (400 MHz, $CDCl_3$) δ 7.96 (dd, J = 8.4, 1.4 Hz, 2H), 7.67 – 7.58 (m, 1H), 7.50 (dd, J = 8.3, 7.0 Hz, 2H), 3.44 – 3.34 (m, 2H), 2.79 (dd, J = 7.7, 6.8 Hz, 2H).

^{13}C{^{1}H} NMR (126 MHz, $CDCl_3$) δ 195.3, 135.6, 133.9, 128.9, 128.0, 119.2, 34.3, 11.8.

GC-HRMS (EI-TOF) m/z: Calcd for $C_{10}H_9NO$ $[M]^+$ 159.0679, found 159.0679

Known compound reported in literature, spectrum matches known spectra.[7]

Ethyl 2-(1,3-dioxoisoindolin-2-yl)-3-(tetrahydrofuran-2-yl)propanoate

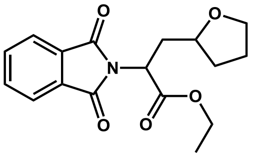

24

Mixture of diastereomers (1:0.92)

Yield = Made according to General procedure A but ran in a 20 mL vessel with an air balloon; time extended to 20 hours and LED positioned directly next to vial (no use of Hepatochem). Purified by column chromatography (5% EtOAc/Hexanes to 10% EtOAc/Hexanes). Isolated as a white solid (101.9 mg, 54%) at 390 nm.

^{1}H NMR (400 MHz, $CDCl_3$) δ 7.83 (m, 4H, Major + Minor), 7.71 (m, 4H, Major + Minor), 5.13 (dd, *J* = 11.5 Hz, *J* = 3.5 Hz, 1H, Major), 4.97 (q, *J* = 4.9 Hz, 0.92H, Minor), 4.18 (m, 4H, Major + Minor), 3.90 (p, 6.6 Hz, 1H, Minor), 3.81 (q, 7.2 Hz, Major), 3.69 (m, 3H, Major + Minor), 3.52 (q, 7.3 Hz, 0.92H), 2.52 (m, 1H, Major), 2.35 (m, 2.9H, Major + Minor), 2.02 (m, 1H, Major), 1.83 (m, 5.2H, Major + Minor), 1.50 (m, 2H, Major + Minor), 1.19 (m, 6.7H, Major + Minor)

^{13}C{^{1}H} NMR (126 MHz, $CDCl_3$) δ 169.6, 169.3, 167.8, 167.7, 134.2, 134.0, 132.2, 132.0, 123.6, 123.5, 75.2, 67.8, 67.8, 61.9, 50.6, 50.1, 34.5, 34.3, 31.8, 31.2, 25.7, 25.3, 14.2.

IR, film (neat, cm-1): 2977, 1714, 1383, 1287, 1197, 1092, 882, 716.

LC-HRMS (ESI-Orbitrap) m/z: Calcd for $C_{17}H_{20}NO_5$ $[M+H]^+$ 318.1336, found 318.1334

Diisopropyl 1-(tetrahydrofuran-2-yl)hydrazine-1,2-dicarboxylate

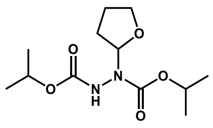

25

Yield = Made according to general procedure A. Purified for a clean NMR with column chromatography using basic alumina with 5% EtOAc/Hexanes to 15% EtOAc/Hexanes. Isolated as a white solid, but material seemed to degrade and mass of product was loss. Final yields of 44% at 456 nm and 36% at 390 nm were determined with benzodioxle I.S. via NMR assay.

^{1}H NMR (500 MHz, $CDCl_3$) δ 6.23 (d, *J* = 76.2 Hz, 1H), 5.99 (s, 1H), 4.95 (d, *J* = 6.1 Hz, 2H), 3.97 (d, *J* = 7.0 Hz, 1H), 3.75 (d, *J* = 7.2 Hz, 1H), 2.22 – 1.78 (m, 4H), 1.25 (s, 12H).

^{13}C{^{1}H} NMR (126 MHz, $CDCl_3$) δ 156.4, 155.1, 70.6, 69.8, 68.6, 28.3, 25.3, 22.0, 21.93, 21.88.

Known compound reported in literature, spectrum matches known spectra.[8]

E. Computational Methods

Gas-phase unrestricted Kohn-Sham density functional theory (DFT)[9] calculations were performed as implemented in the QChem 5.3 package.[10] Following the approach of Dai et al.[11], who studied the photochemistry of $FeCl_4^-$ and related compounds, a basis set and functional combination of def2-SVP[12]/ωB97X-D[13] was used for the geometry optimization while def2-TZVPP[12]/ωB97X-D[13] was used for time-dependent DFT (TDDFT) and hole-electron analyses.[14–16] The 0.001 absorbance strength threshold matches Dai et al.[11] Results shown do not use the Tamm-Dancoff Approximation.[17] There were five unpaired electrons. Structures were hand drawn in IQmol[18] and optimized in QChem; their geometries are provided below. $FeCl_4^-$ and $FeCl_3Br^-$ were geometry optimized maintaining their T_d and C_{3v} symmetries, respectively.

The TDDFT calculations on the two compounds were redone in an acetonitrile solvent using the conductor-like polarizable continuum model (CPCM), as implemented in QChem. For the solvent model, the dielectric constant was set to 36.55, and optical dielectric to 1.796136.[19,20] In the range of interest (300–550nm) wavelengths redshifted by < 5nm with similarly minor shifts for peak height.

To construct the potential energy surfaces in Figure 3, we performed TDDFT along the Fe-Br reaction coordinate of $FeCl_3Br^-$. This involved constraining the length of the Fe-Br bond and fixing the symmetry. The energies for the red line indicating Fe-Br σ* character were joined up qualitatively based on matching the character of the orbitals involved in the excitation. The other purple lines are joined up by energy rank.

In Figure S3, to provide a comparison to the experimentally determined spectra, shown in red, the purple curve is a gaussian linewidth broadening of the theoretically calculated TDDFT impulses for $FeCl_3Br^-$. The broadened TDDFT impulse spectrum $\left(S(x)\right)$ was calculated as a sum of scaled probability density functions centered on the impulses, and is given by

$$\mathrm{S}(x) \propto \sum_{i}^{N} f_i \frac{\exp\left(-\frac{x - E_i}{2\sigma^2}\right)}{\sqrt{(2\pi\sigma^2)}}. \qquad 1$$

Where f_i and E_i are the oscillator strength and excitation energy of excitation i respectively, N is the number of excitations, and the scale (σ) was set to 0.186 to be visually consistent with the graphical software IQMol.[18] The curve is then converted to wavelength and normalized such that the tallest visible part of the function is 1.

Computed UV-Vis spectra for $FeCl_4^-$ and $FeCl_3Br^-$ complexes.

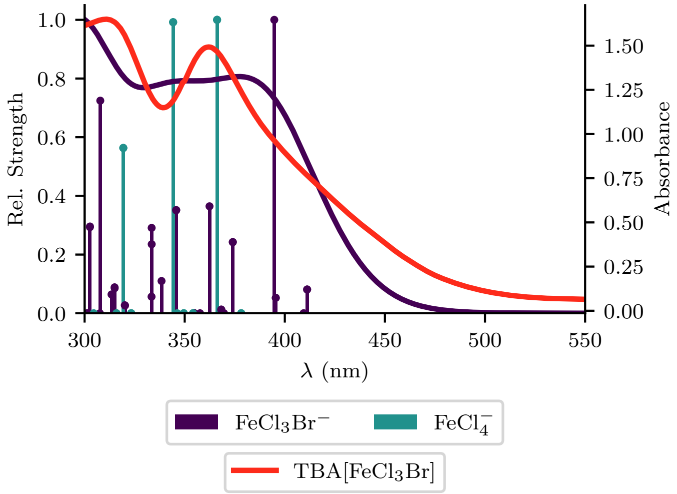

Figure S3. TDDFT impulses from $FeCl_4^-$ and $FeCl_3Br^-$ normalized to their strongest impulse in the given wavelength range. Experimental UV-Vis data corresponds to the right axis.

def2-SVP/ωB97X-D optimized XYZ coordinates (Å) for $FeCl_3Br^-$ and $FeCl_4^-$ complexes.

Atom	X	Y	Z
Fe	0.0000000000	0.0000000000	-0.4141977652
Cl	2.0982961880	0.0000000000	-1.1407335652
Cl	-1.0491480940	1.8171778035	-1.1407335652
Cl	-1.0491480940	-1.8171778035	-1.1407335652
Br	0.0000000000	0.0000000000	1.9699015348

Table S4: Geometry Optimized Coordinates of $FeCl_3Br^-$

Atom	X	Y	Z
Fe	0.0000000000	0.0000000000	0.0000000000
Cl	-1.2854803206	1.2854803206	-1.2854803206
Cl	1.2854803206	-1.2854803206	-1.2854803206
Cl	1.2854803206	1.2854803206	1.2854803206
Cl	-1.2854803206	-1.2854803206	1.2854803206

Table S5: Geometry Optimized Coordinates of $FeCl_4^-$

Table S6: Tabulated data for the hole-electron analysis of $FeCl_3Br^-$

λ (nm)	Strengths	Strength > 0.001	Fe β e⁻	Br β e⁻	Cl β e⁻	Fe β h⁺	Br β h⁺	Cl β h⁺
411.2	1.97E-03	Yes	83%	7%	10%	3%	69%	27%
411.1	1.96E-03	Yes	83%	7%	10%	3%	69%	27%
395.5	1.28E-03	Yes	73%	14%	12%	4%	73%	24%
395.5	1.27E-03	Yes	73%	14%	12%	4%	73%	24%
394.7	2.43E-02	Yes	83%	6%	11%	4%	60%	36%
374.1	5.89E-03	Yes	84%	5%	11%	1%	3%	95%
374.0	5.89E-03	Yes	84%	5%	11%	1%	3%	95%
362.4	8.85E-03	Yes	75%	3%	22%	2%	53%	45%
345.8	8.54E-03	Yes	77%	4%	19%	8%	15%	77%
345.7	8.52E-03	Yes	77%	4%	19%	8%	15%	77%
338.4	2.67E-03	Yes	81%	7%	12%	21%	16%	63%
333.5	5.71E-03	Yes	82%	4%	14%	21%	16%	63%
333.5	7.06E-03	Yes	82%	4%	14%	21%	16%	63%
333.4	1.37E-03	Yes	83%	4%	13%	20%	17%	63%
315.0	2.07E-03	Yes	75%	8%	17%	21%	18%	61%
315.0	2.15E-03	Yes	75%	8%	17%	21%	17%	61%
313.4	1.56E-03	Yes	79%	2%	18%	22%	24%	55%
307.8	1.76E-02	Yes	82%	6%	12%	23%	10%	67%
302.7	7.18E-03	Yes	78%	4%	18%	21%	21%	58%
302.7	7.13E-03	Yes	78%	4%	18%	21%	21%	58%
409.4	1.36E-06	No	84%	6%	10%	3%	67%	30%
369.6	3.70E-09	No	76%	3%	21%	2%	47%	52%
368.3	3.01E-04	No	77%	1%	21%	2%	47%	50%
368.3	3.03E-04	No	77%	1%	21%	2%	47%	50%
357.6	8.12E-08	No	75%	10%	15%	3%	6%	92%
354.7	1.68E-05	No	81%	5%	14%	20%	28%	52%
354.5	2.17E-05	No	81%	5%	14%	20%	28%	52%
320.2	6.43E-04	No	81%	3%	16%	22%	7%	71%
320.1	6.60E-04	No	81%	3%	16%	22%	7%	71%
307.6	0.00E+00	No	82%	4%	14%	21%	15%	64%
301.3	8.40E-09	No	78%	4%	18%	20%	10%	70%

Table S7: Natural Transition Orbitals (NTOs) of $FeCl_3Br^-$ for the first four distinct transitions with strengths greater than 1×10^{-3}. The particle and hole NTOs are the pair of transformed virtual and occupied orbitals, respectively, obtained using the transition density matrix for a given excitation. They represent the excited electron, and the hole left behind, respectively.

λ	Particle NTO	Hole NTO
411 nm	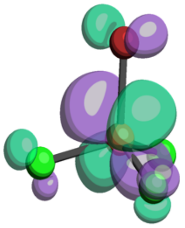	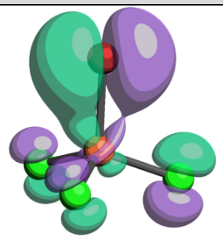
395.5 nm	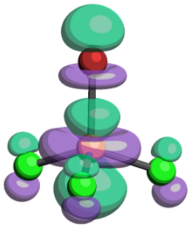	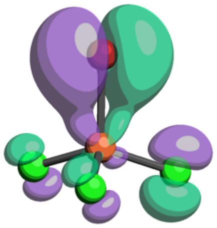
394.7 nm	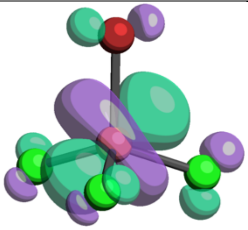	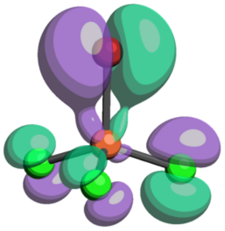
374 nm	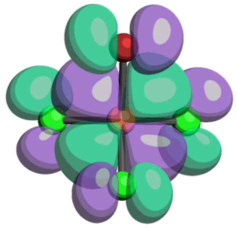	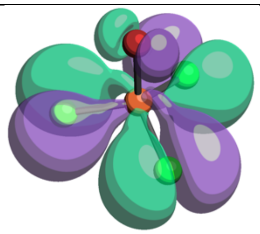

F. References

(1) Gust, R. A New Approach in Cancer Treatment: Discovery of Chlorido[N,N′-Disalicylidene-1,2-Phenylenediamine]Iron(III) Complexes as Ferroptosis Inducers. *J. Med. Chem* **2019**, *62* (17), 8053–8061.

(2) Akiyama, T.; Uchikura, T.; Moriyama, K.; Toda, M.; Mouri, T.; Ibanez, I. Benzothiazolines as Radical Transfer Reagents: Hydroalkylation and Hydroacylation of Alkenes by Radical Generation under Photoirradiation Conditions. *Chem. Commun.* **2019**, No. 55, 11171.

(3) Jin, Y.; Chen, J.; Gan, Z.; Zhang, Y.; Chen, Z.; Liu, S.; Cui, R.; Xue, Z.; Sun, H.; Shi, L.; jiang, W. Iron-Catalyzed Photoredox Alcohol α-C−H Alkylation and TandemIntramolecular Cyclization: Facile Access to Multisubstituted 2,3-Dihydrofurans and γ-Butyrolactones. *Org. Lett.* **2024**, No. 26, 5329–5334.

(4) Gong, L.; Dai, Z.; Zhang, S.; Hong, X.; Wang, P. A Practical FeCl3/HCl Photocatalyst for Versa Tile Aliphatic C–H Functionalization. *Chem. Catal.* **2022**, 1211–1222.

(5) Huang, D.; Zhang, Y.; Yanx, X.; Wu, J. Aerobic C–H Functionalization Using Pyrenedione as the Photocatalyst. *Synthesis* **2020**, No. 52, 2512–2520.

(6) Walton, J.; Manley, D.; McBurney, R.; Miller, P. Titania-Promoted Carboxylic Acid Alkylations of Alkenes andCascade Addition−Cyclizations. *J. Org. Chem.* **2014**, *79*, 1386–1398.

(7) Reiser, O.; Chinchole, A.; Henriquez, M.; Cortes-Arriagada, D.; Cabrera, A. Iron(III)-Light-Induced Homolysis: A Dual Photocatalytic Approach for the Hydroacylation of Alkenes Using Acyl Radicals via Direct HAT from Aldehydes. *ACS Catal.* **2022**, *12*, 13549–13554.

(8) Kokotos, C.; Papadopoulos, G.; Kokotou, M.; Spiliopoulou, N.; Nikitas, N.; Voutyritsa, E.; Tzaras, D.; Kaplaneris, N. Phenylglyoxylic Acid: An Efficient Initiator for thePhotochemical Hydrogen Atom Transfer C. *ChemSusChem* **2020**, No. 13, 5934–5944.

(9) Kohn, W.; Sham, L. J. Self-Consistent Equations Including Exchange and Correlation Effects. *Phys. Rev.* **1965**, *140* (4A), A1133–A1138. https://doi.org/10.1103/PhysRev.140.A1133.

(10) Epifanovsky, E.; Gilbert, A. T. B.; Feng, X.; Lee, J.; Mao, Y.; Mardirossian, N.; Pokhilko, P.; White, A. F.; Coons, M. P.; Dempwolff, A. L.; Gan, Z.; Hait, D.; Horn, P. R.; Jacobson, L. D.; Kaliman, I.; Kussmann, J.; Lange, A. W.; Lao, K. U.; Levine, D. S.; Liu, J.; McKenzie, S. C.; Morrison, A. F.; Nanda, K. D.; Plasser, F.; Rehn, D. R.; Vidal, M. L.; You, Z.-Q.; Zhu, Y.; Alam, B.; Albrecht, B. J.; Aldossary, A.; Alguire, E.; Andersen, J. H.; Athavale, V.; Barton, D.; Begam, K.; Behn, A.; Bellonzi, N.; Bernard, Y. A.; Berquist, E. J.; Burton, H. G. A.; Carreras, A.; Carter-Fenk, K.; Chakraborty, R.; Chien, A. D.; Closser, K. D.; Cofer-Shabica, V.; Dasgupta, S.; de Wergifosse, M.; Deng, J.; Diedenhofen, M.; Do, H.; Ehlert, S.; Fang, P.-T.; Fatehi, S.; Feng, Q.; Friedhoff, T.; Gayvert, J.; Ge, Q.; Gidofalvi, G.; Goldey, M.; Gomes, J.; González-Espinoza, C. E.; Gulania, S.; Gunina, A. O.; Hanson-Heine, M. W. D.; Harbach, P. H. P.; Hauser, A.; Herbst, M. F.; Hernández Vera, M.; Hodecker, M.; Holden, Z. C.; Houck, S.; Huang, X.; Hui, K.; Huynh, B. C.; Ivanov, M.; Jász, Á.; Ji, H.; Jiang, H.; Kaduk, B.; Kähler, S.; Khistyaev, K.; Kim, J.; Kis, G.; Klunzinger, P.; Koczor-Benda, Z.; Koh, J. H.; Kosenkov, D.; Koulias, L.; Kowalczyk, T.; Krauter, C. M.; Kue, K.; Kunitsa, A.; Kus, T.; Ladjánszki, I.; Landau, A.; Lawler, K. V.; Lefrancois, D.; Lehtola, S.; Li, R. R.; Li, Y.-P.; Liang, J.; Liebenthal, M.; Lin, H.-H.; Lin, Y.-S.; Liu, F.; Liu, K.-Y.; Loipersberger, M.; Luenser, A.; Manjanath, A.; Manohar, P.; Mansoor, E.; Manzer, S. F.; Mao, S.-P.; Marenich, A. V.; Markovich, T.; Mason, S.; Maurer, S. A.; McLaughlin, P. F.; Menger, M. F. S. J.; Mewes, J.-M.; Mewes, S. A.; Morgante, P.; Mullinax, J. W.; Oosterbaan, K. J.; Paran, G.; Paul, A. C.; Paul, S. K.; Pavošević, F.; Pei, Z.; Prager, S.; Proynov, E. I.; Rák, Á.; Ramos-Cordoba, E.; Rana, B.; Rask, A. E.; Rettig, A.; Richard, R. M.; Rob, F.; Rossomme, E.; Scheele, T.; Scheurer, M.; Schneider, M.; Sergueev, N.; Sharada, S. M.; Skomorowski, W.; Small, D. W.; Stein, C. J.; Su, Y.-C.; Sundstrom, E. J.; Tao, Z.; Thirman, J.; Tornai, G. J.; Tsuchimochi, T.; Tubman, N. M.; Veccham, S. P.; Vydrov, O.; Wenzel, J.; Witte, J.; Yamada, A.; Yao, K.; Yeganeh, S.; Yost, S. R.; Zech, A.; Zhang, I. Y.; Zhang, X.; Zhang, Y.; Zuev, D.; Aspuru-Guzik, A.; Bell, A. T.; Besley, N. A.; Bravaya, K. B.; Brooks, B. R.; Casanova, D.; Chai, J.-D.; Coriani, S.; Cramer, C. J.; Cserey, G.; DePrince, A. E., III; DiStasio, R. A., Jr.; Dreuw, A.; Dunietz, B. D.; Furlani, T. R.; Goddard, W. A., III; Hammes-Schiffer, S.; Head-Gordon,

T.; Hehre, W. J.; Hsu, C.-P.; Jagau, T.-C.; Jung, Y.; Klamt, A.; Kong, J.; Lambrecht, D. S.; Liang, W.; Mayhall, N. J.; McCurdy, C. W.; Neaton, J. B.; Ochsenfeld, C.; Parkhill, J. A.; Peverati, R.; Rassolov, V. A.; Shao, Y.; Slipchenko, L. V.; Stauch, T.; Steele, R. P.; Subotnik, J. E.; Thom, A. J. W.; Tkatchenko, A.; Truhlar, D. G.; Van Voorhis, T.; Wesolowski, T. A.; Whaley, K. B.; Woodcock, H. L., III; Zimmerman, P. M.; Faraji, S.; Gill, P. M. W.; Head-Gordon, M.; Herbert, J. M.; Krylov, A. I. Software for the Frontiers of Quantum Chemistry: An Overview of Developments in the Q-Chem 5 Package. *J. Chem. Phys.* **2021**, *155* (8), 084801. https://doi.org/10.1063/5.0055522.

(11) Dai, Z.-Y.; Zhang, S.-Q.; Hong, X.; Wang, P.-S.; Gong, L.-Z. A Practical FeCl3/HCl Photocatalyst for Versatile Aliphatic C–H Functionalization. *Chem Catal.* **2022**, *2* (5), 1211–1222. https://doi.org/10.1016/j.checat.2022.03.020.

(12) Weigend, F.; Ahlrichs, R. Balanced Basis Sets of Split Valence, Triple Zeta Valence and Quadruple Zeta Valence Quality for H to Rn: Design and Assessment of Accuracy. *Phys. Chem. Chem. Phys.* **2005**, *7* (18), 3297–3305. https://doi.org/10.1039/B508541A.

(13) Chai, J.-D.; Head-Gordon, M. Long-Range Corrected Hybrid Density Functionals with Damped Atom–Atom Dispersion Corrections. *Phys. Chem. Chem. Phys.* **2008**, *10* (44), 6615–6620. https://doi.org/10.1039/B810189B.

(14) Dreuw, A.; Head-Gordon, M. Single-Reference Ab Initio Methods for the Calculation of Excited States of Large Molecules. *Chem. Rev.* **2005**, *105* (11), 4009–4037. https://doi.org/10.1021/cr0505627.

(15) Liu, F.; Gan, Z.; Shao, Y.; Hsu, C.-P.; Dreuw, A.; Head-Gordon, M.; Miller, B. T.; Brooks, B. R.; Yu, J.-G.; Furlani, T. R.; Kong, J. A Parallel Implementation of the Analytic Nuclear Gradient for Time-Dependent Density Functional Theory within the Tamm–Dancoff Approximation. *Mol. Phys.* **2010**, *108* (19–20), 2791–2800. https://doi.org/10.1080/00268976.2010.526642.

(16) Richard, R. M.; Herbert, J. M. Time-Dependent Density-Functional Description of the 1La State in Polycyclic Aromatic Hydrocarbons: Charge-Transfer Character in Disguise? *J. Chem. Theory Comput.* **2011**, *7* (5), 1296–1306. https://doi.org/10.1021/ct100607w.

(17) Hirata, S.; Head-Gordon, M. Time-Dependent Density Functional Theory within the Tamm–Dancoff Approximation. *Chem. Phys. Lett.* **1999**, *314* (3), 291–299. https://doi.org/10.1016/S0009-2614(99)01149-5.

(18) Gilbert, A.; Berquist, E.; Lehtola, S. IQmol. http://iqmol.org/.

(19) Wohlfarth, C. Refractive Index of Acetonitrile. In *Optical Constants*; Lechner, M. D., Ed.; Springer Berlin Heidelberg: Berlin, Heidelberg, 2017; pp 82–82. https://doi.org/10.1007/978-3-662-49236-9_77.

(20) Wohlfarth, C. Static Dielectric Constant of Acetonitrile. In *Static Dielectric Constants of Pure Liquids and Binary Liquid Mixtures*; Lechner, M. D., Ed.; Springer Berlin Heidelberg: Berlin, Heidelberg, 2015; pp 19–19. https://doi.org/10.1007/978-3-662-48168-4_18.

(21) Schlegel, M., Qian, S., and Nicewicz, D. A. Aliphatic C-H Functionalization Using Pyridine *N*-Oxides as H-Atom Abstraction Agents. *ACS Catal.* **2022**, *12* (16), 10499-10505. https://doi.org/10.1021/acscatal.2c02997

G. NMR Spectra

^{1}H (400 MHz) and $^{13}C\{^{1}H\}$ (101 MHz) data for compound **4** in $CDCl_3$:

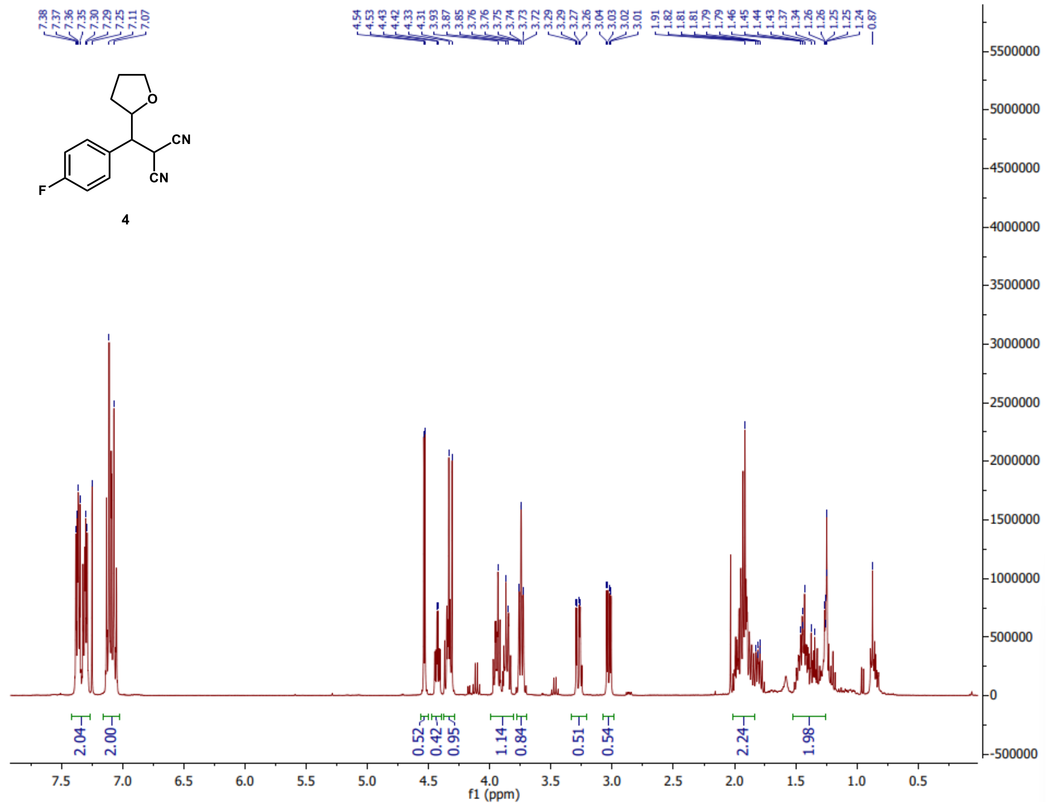

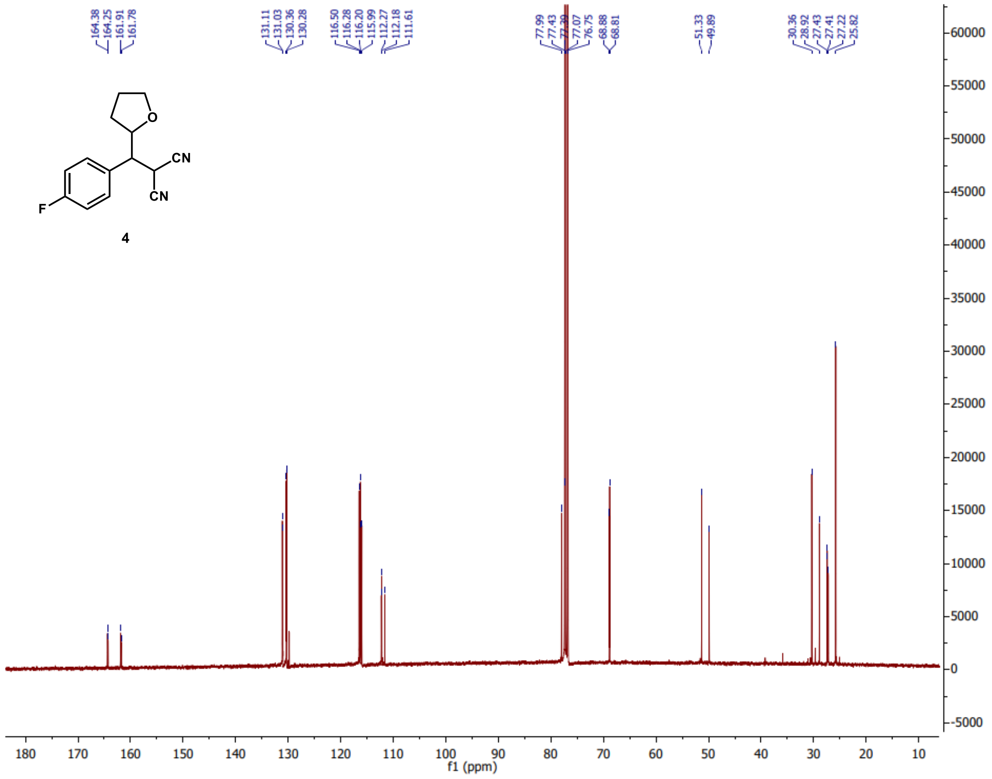

^{1}H (400 MHz) and ^{13}C{^{1}H} (101 MHz) data for compound **10** in $CDCl_3$:

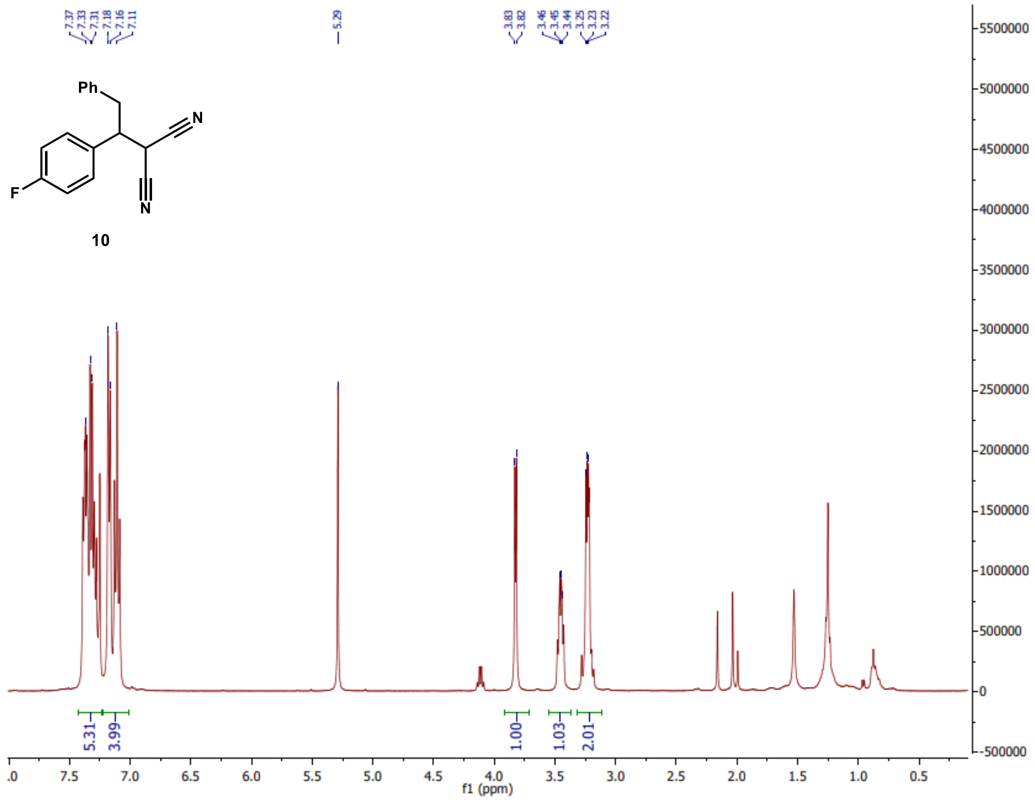

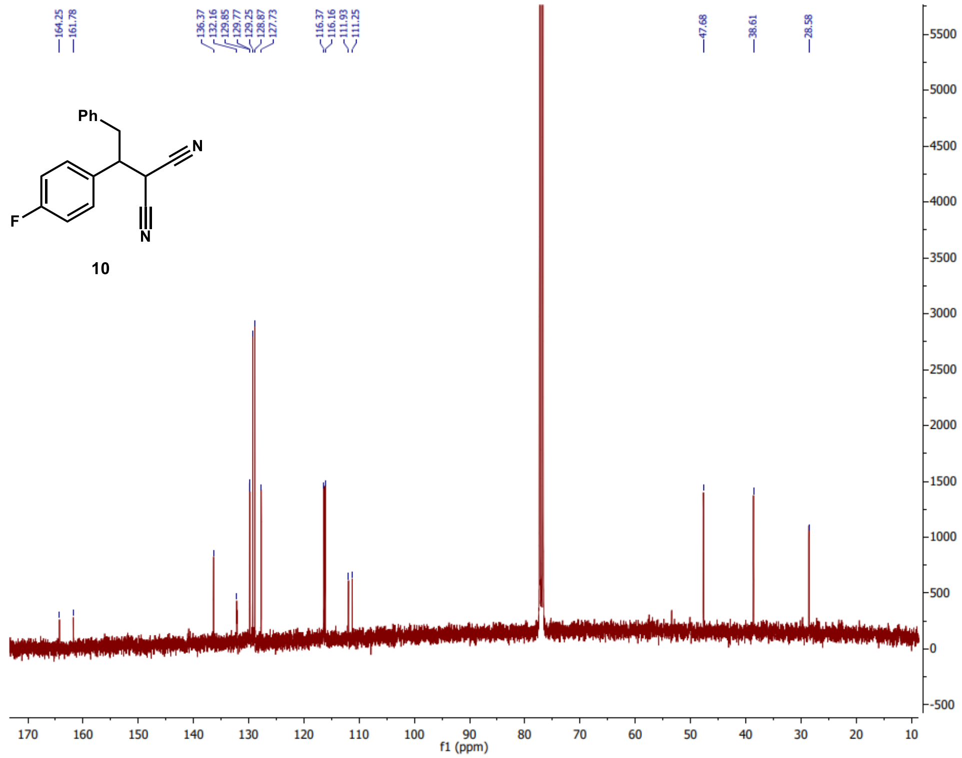

^{1}H (400 MHz) and $^{13}C\{^{1}H\}$ (126 MHz) data for compound **11** in $CDCl_3$:

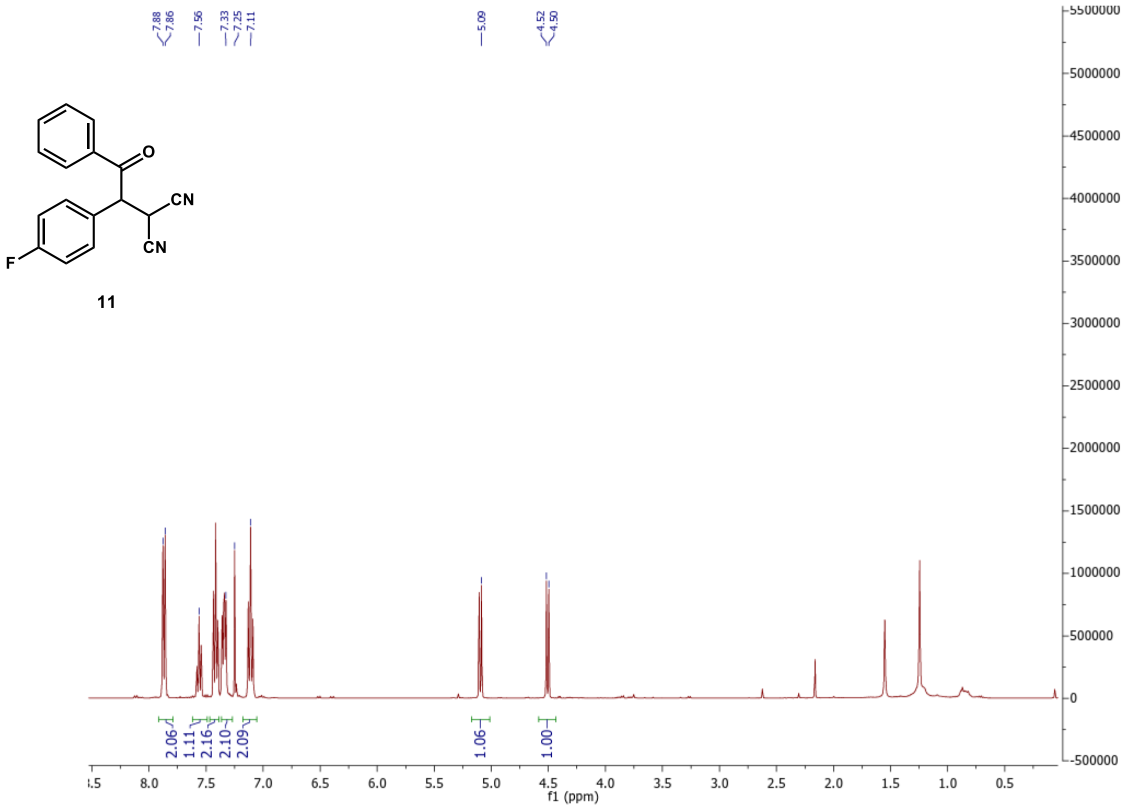

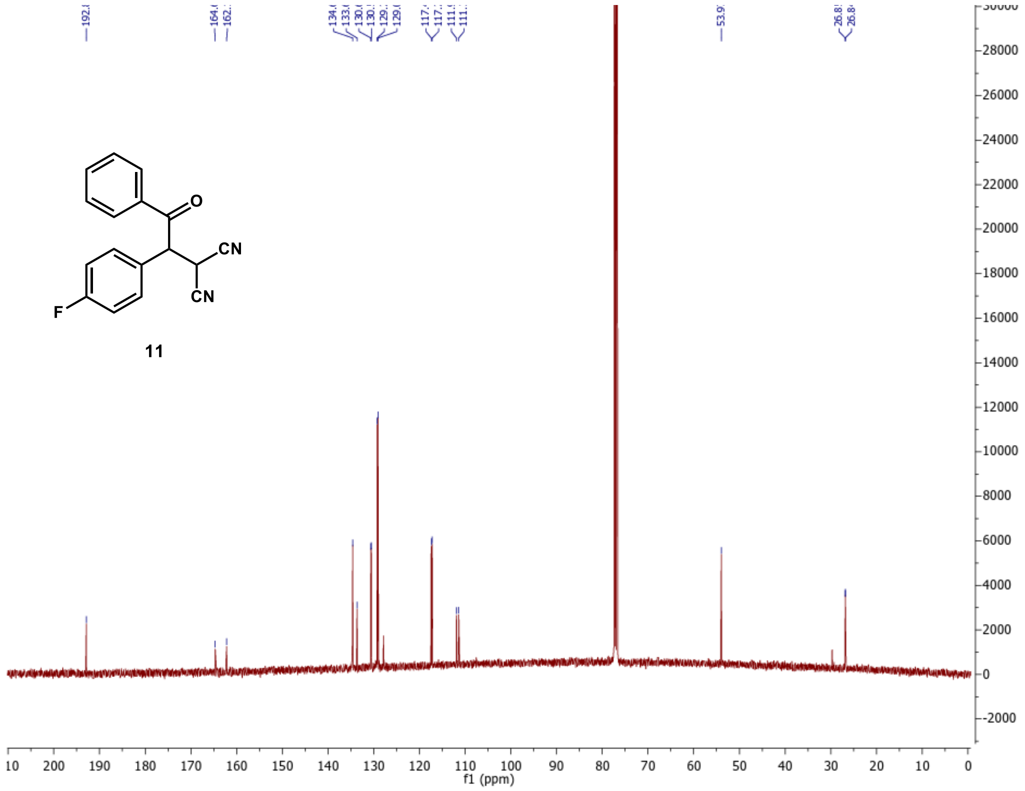

^{1}H (400 MHz) data for compound **12** in $CDCl_3$:

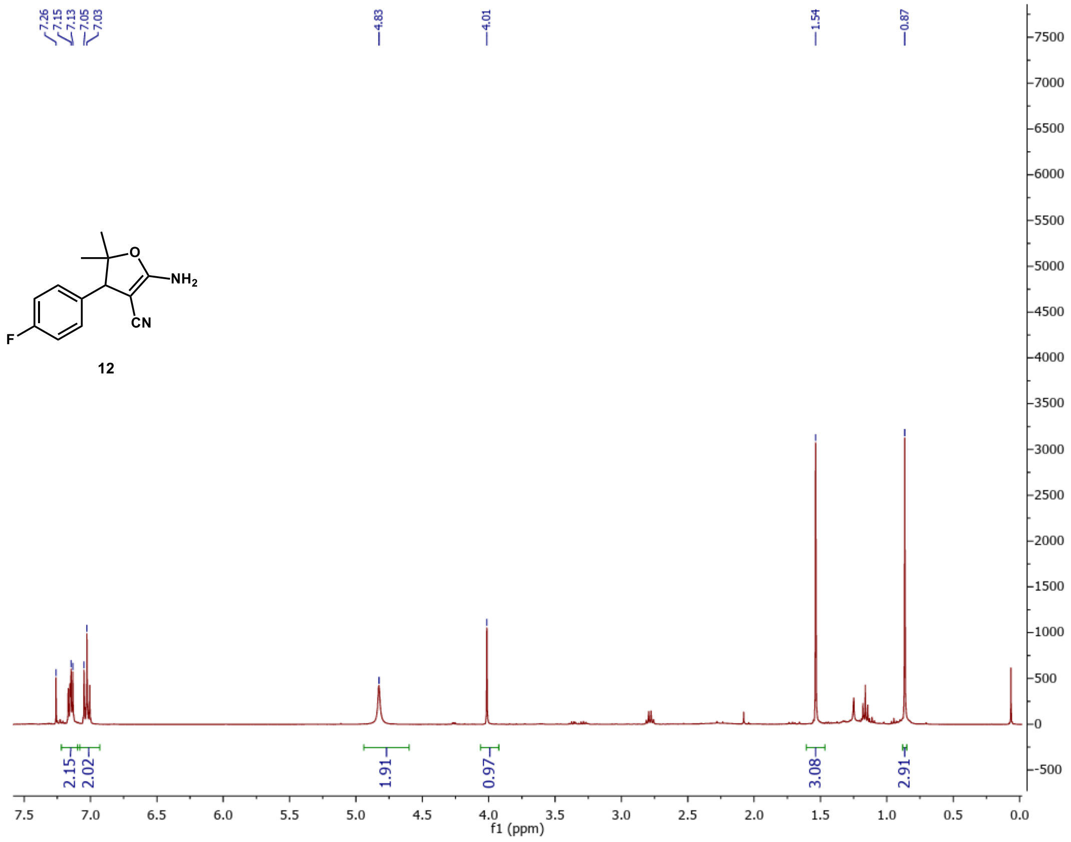

^{1}H (400 MHz) and ^{13}C{^{1}H} (126 MHz) data for compound **13** in $CDCl_3$:

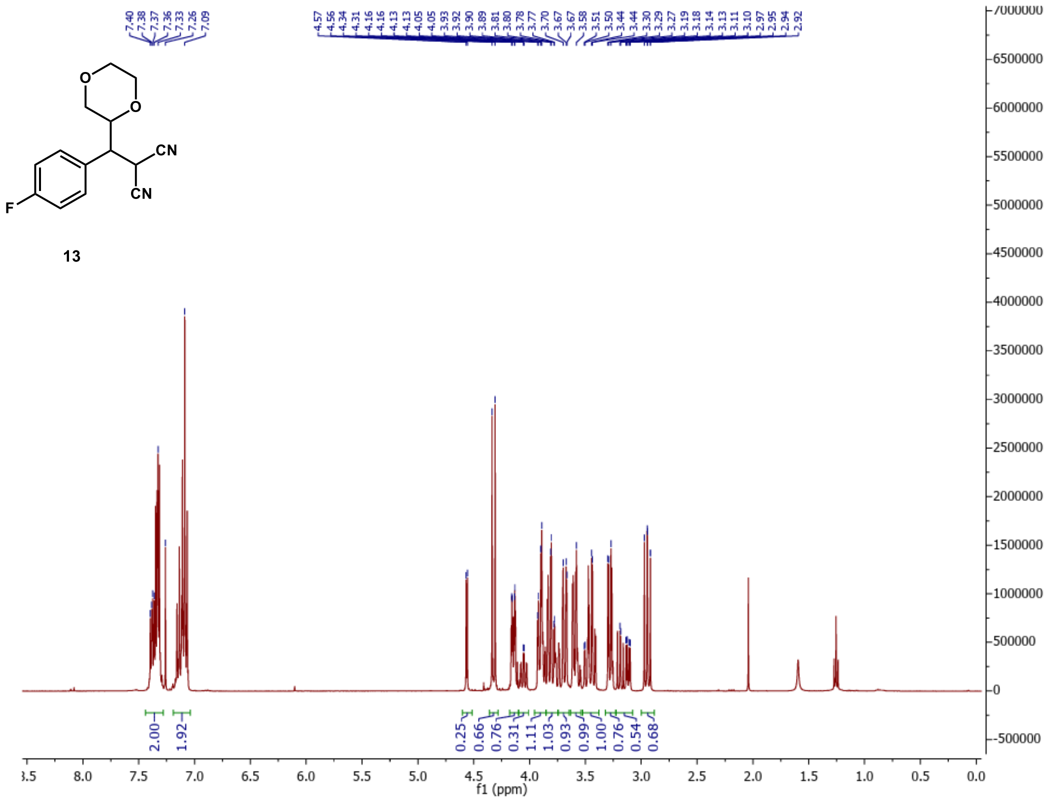

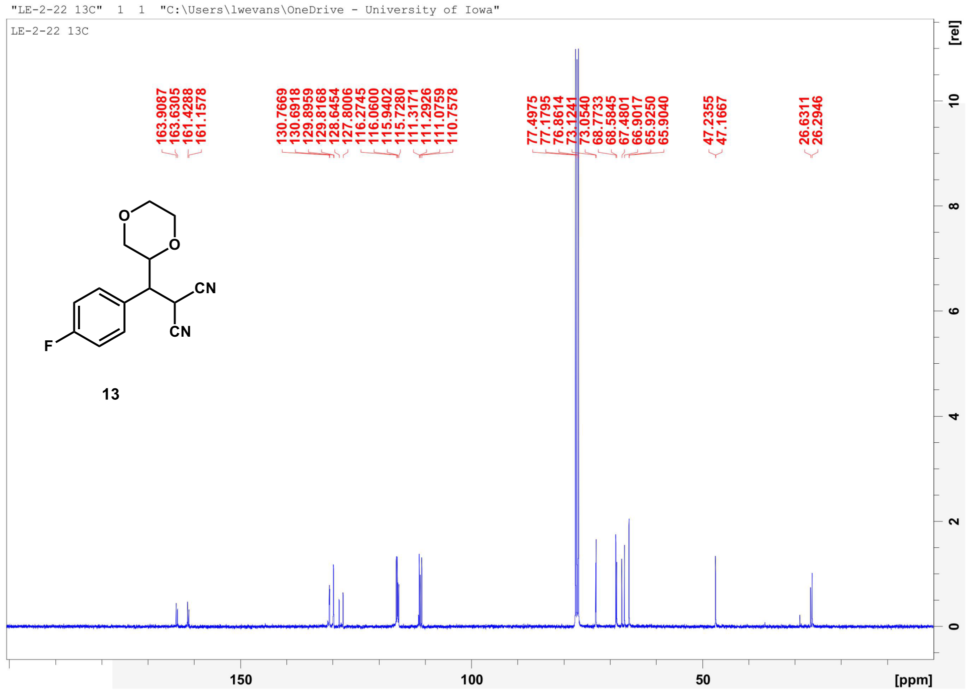

^{1}H (500 MHz) and $^{13}C\{^{1}H\}$ (126 MHz) data for compound **14** in $CDCl_3$:

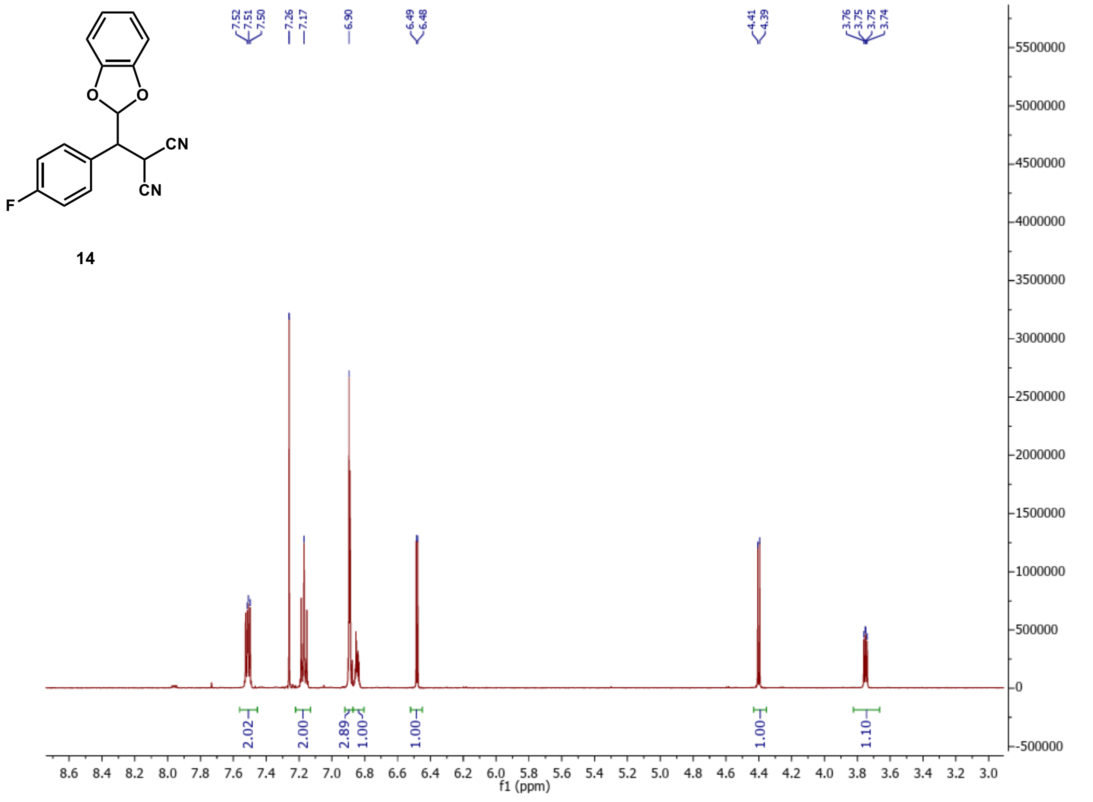

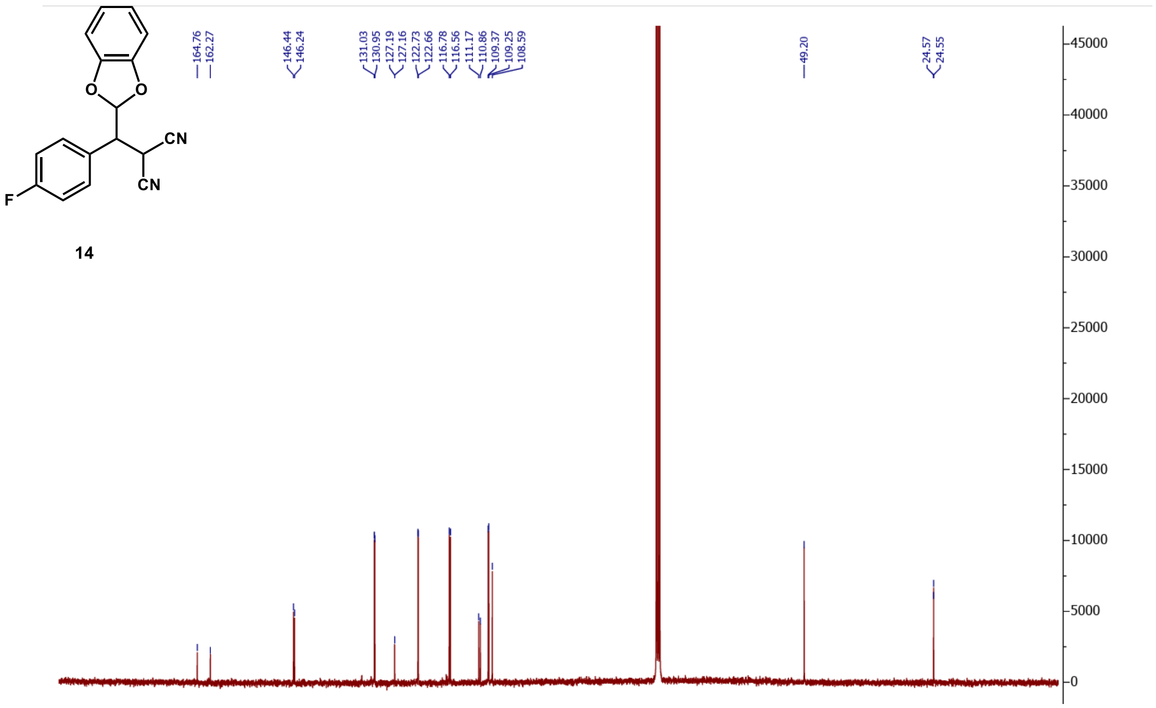

^{1}H (500 MHz) and ^{13}C{^{1}H} (126 MHz) data for compound **15** in $CDCl_3$:

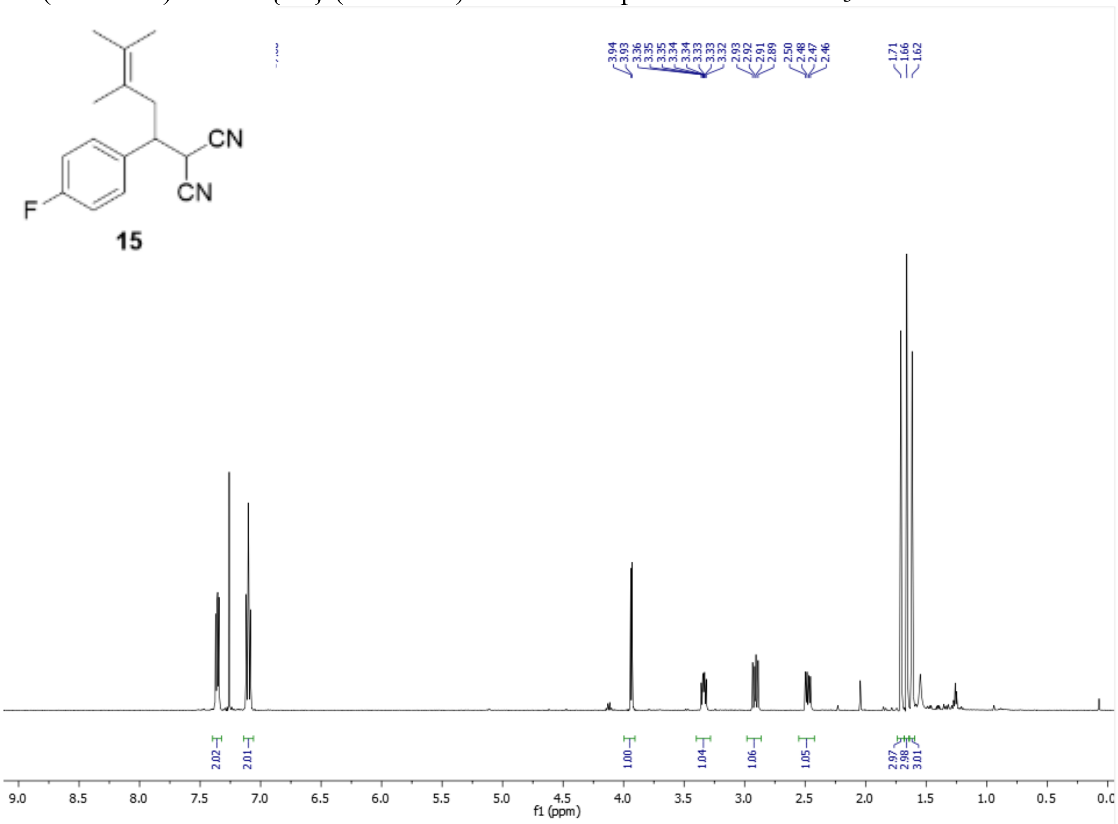

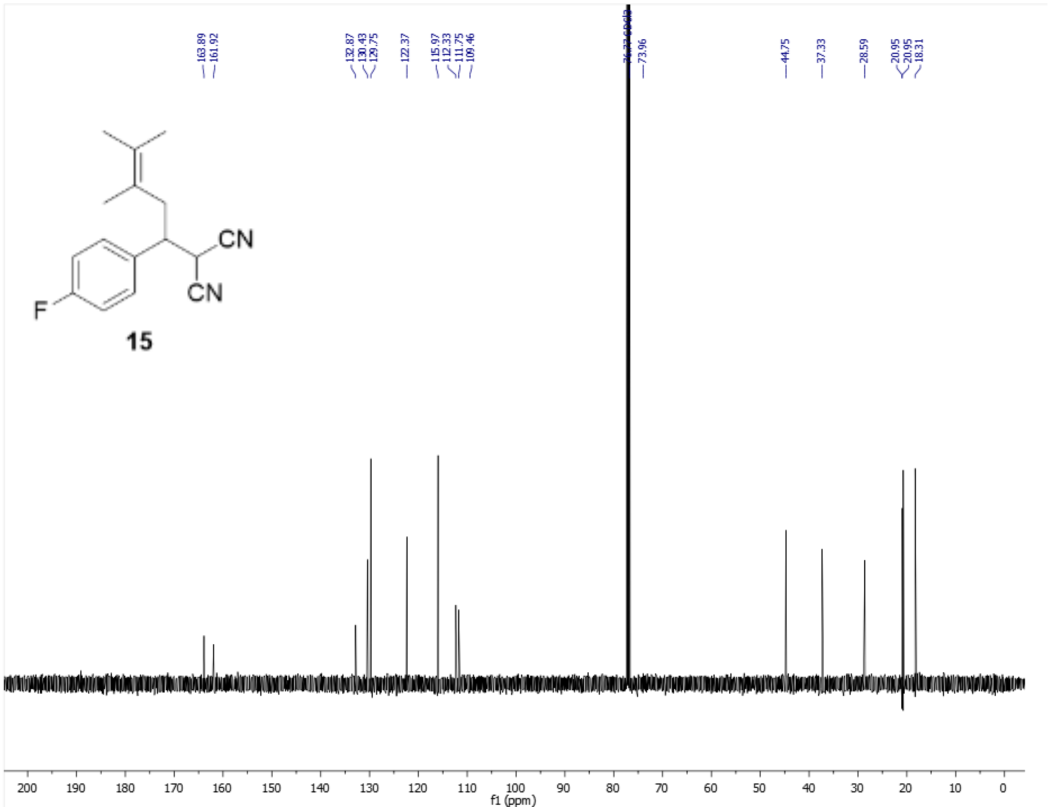

^{1}H (500 MHz) and $^{13}C\{^{1}H\}$ (126 MHz) data for compound **16** in $CDCl_3$:

Diastereomer 1 (less polar)

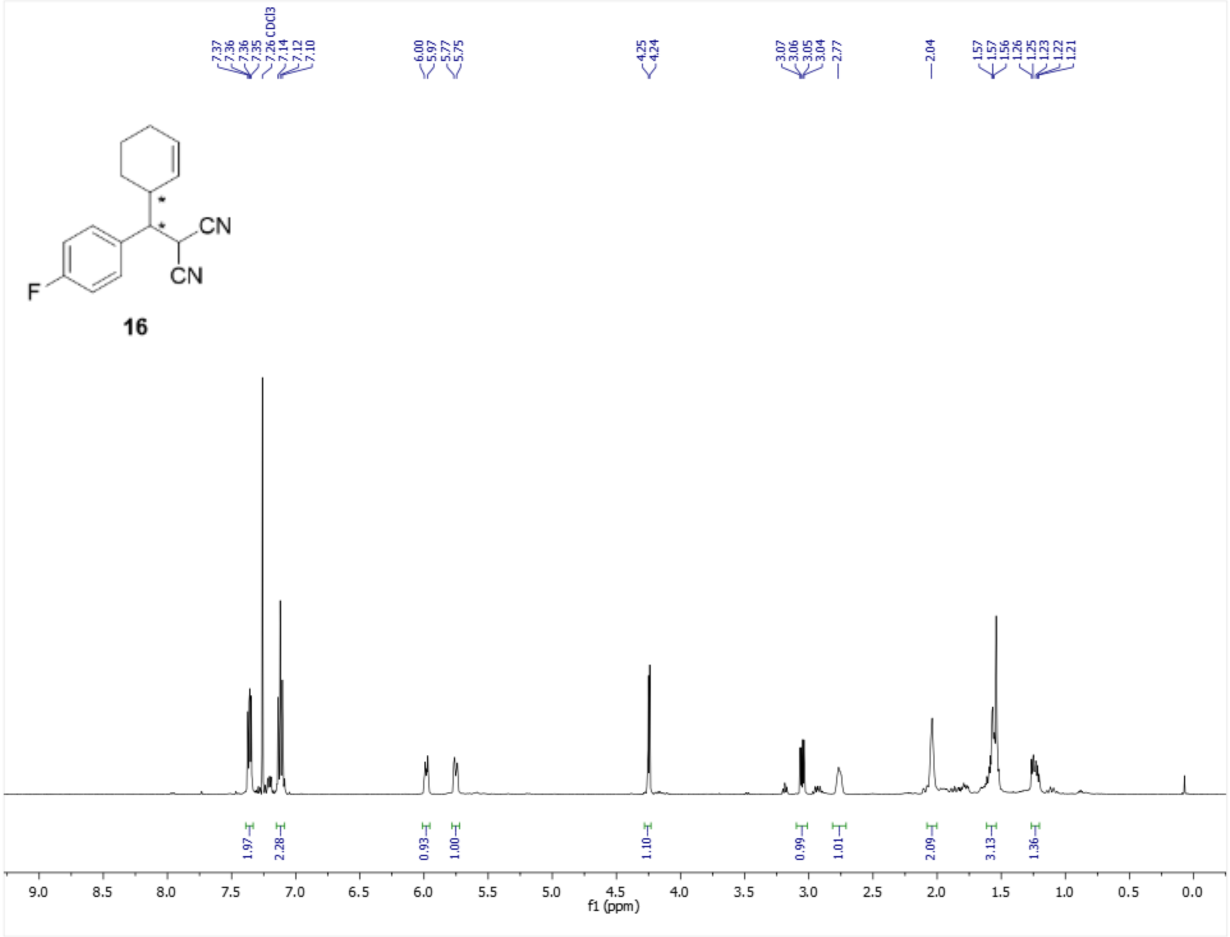

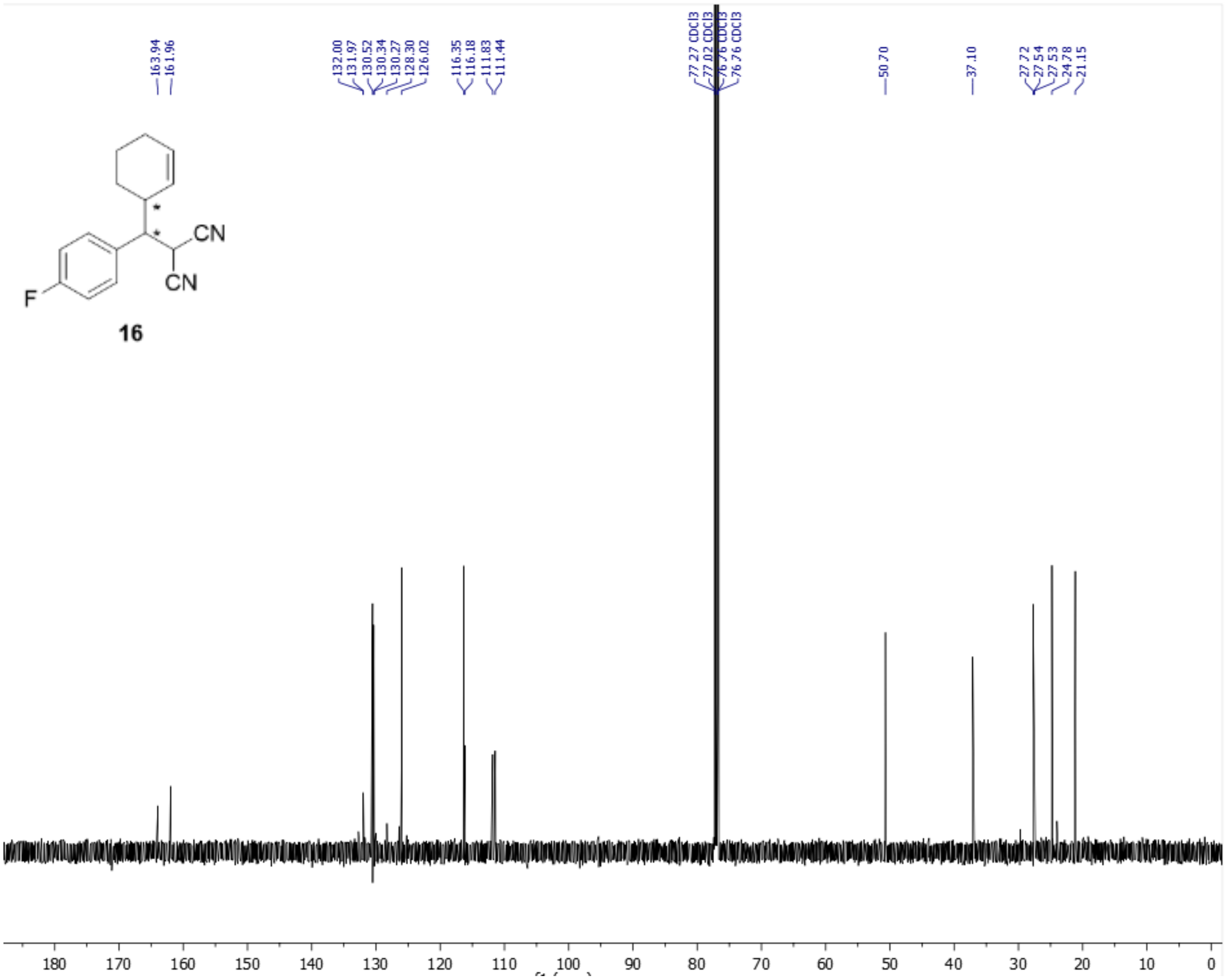
163.94
161.96
132.00
131.97
130.52
130.34
130.27
128.30
126.02
116.35
116.18
111.83
111.44
77.27 CDCl3
77.02 CDCl3
76.76 CDCl3
76.76 CDCl3
50.70
37.10
27.72
27.54
27.53
24.78
21.15
CN
CN
F
16
180
170
160
150
140
130
120
110
100
90
80
70
60
50
40
30
20
10
0

Diastereomer 2 (more polar)

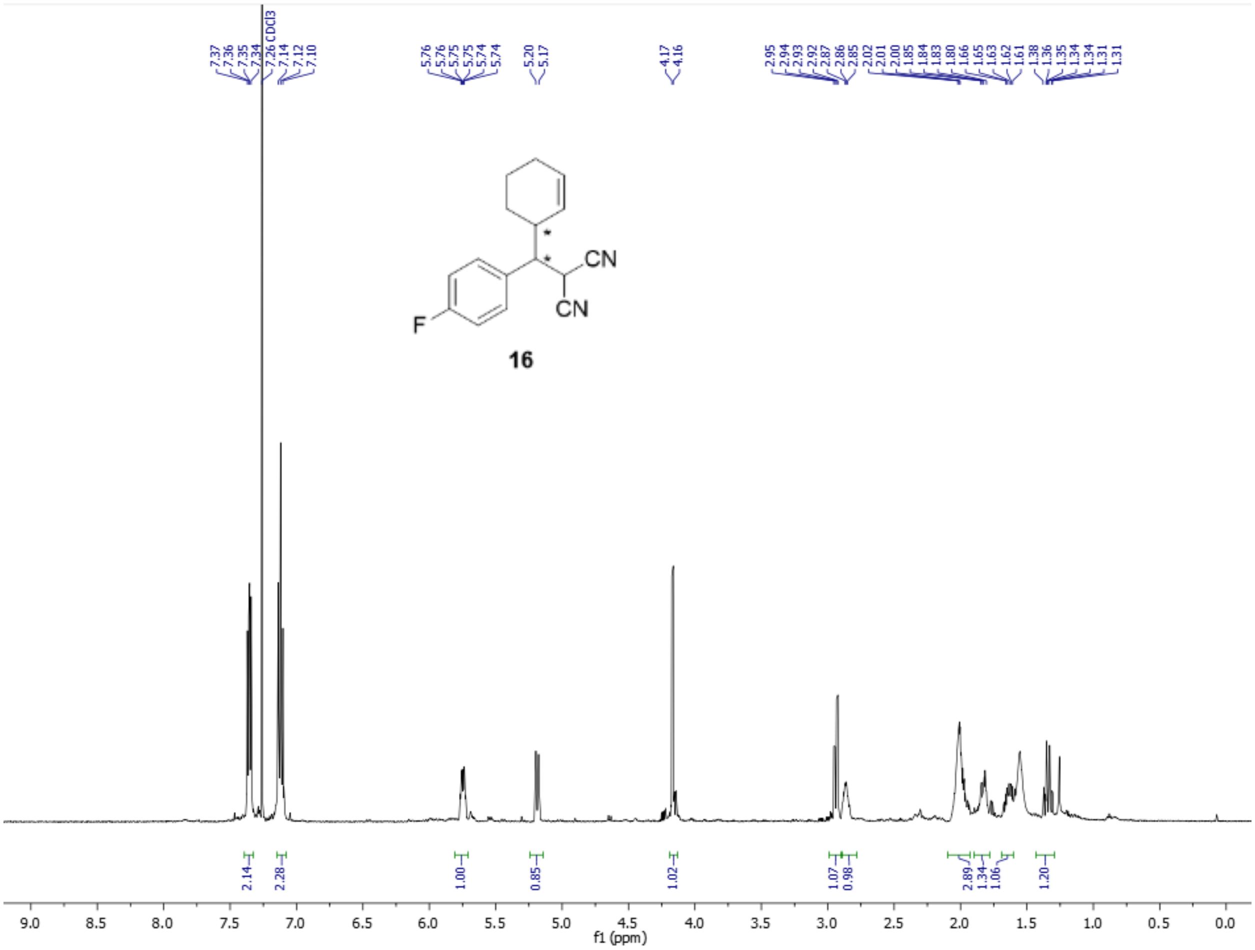

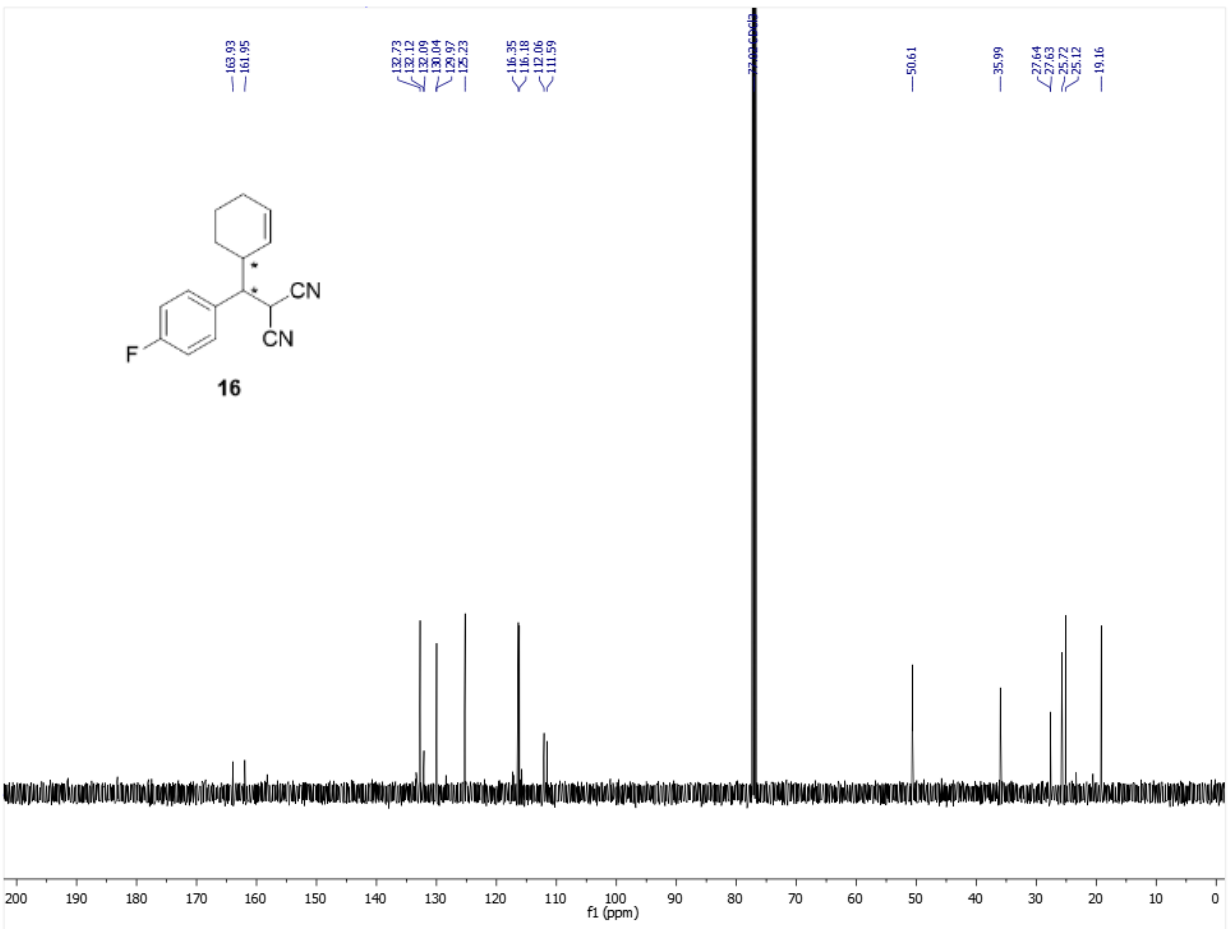

163.93
161.95
132.73
132.12
132.09
130.04
129.97
125.23
116.35
116.18
112.06
111.59
50.61
35.99
27.64
27.63
25.72
25.12
19.16
CN
CN
F
16
200 190 180 170 160 150 140 130 120 110 100 90 80 70 60 50 40 30 20 10 0
f1 (ppm)

^{1}H (400 MHz) data for compound **17** in $CDCl_3$:

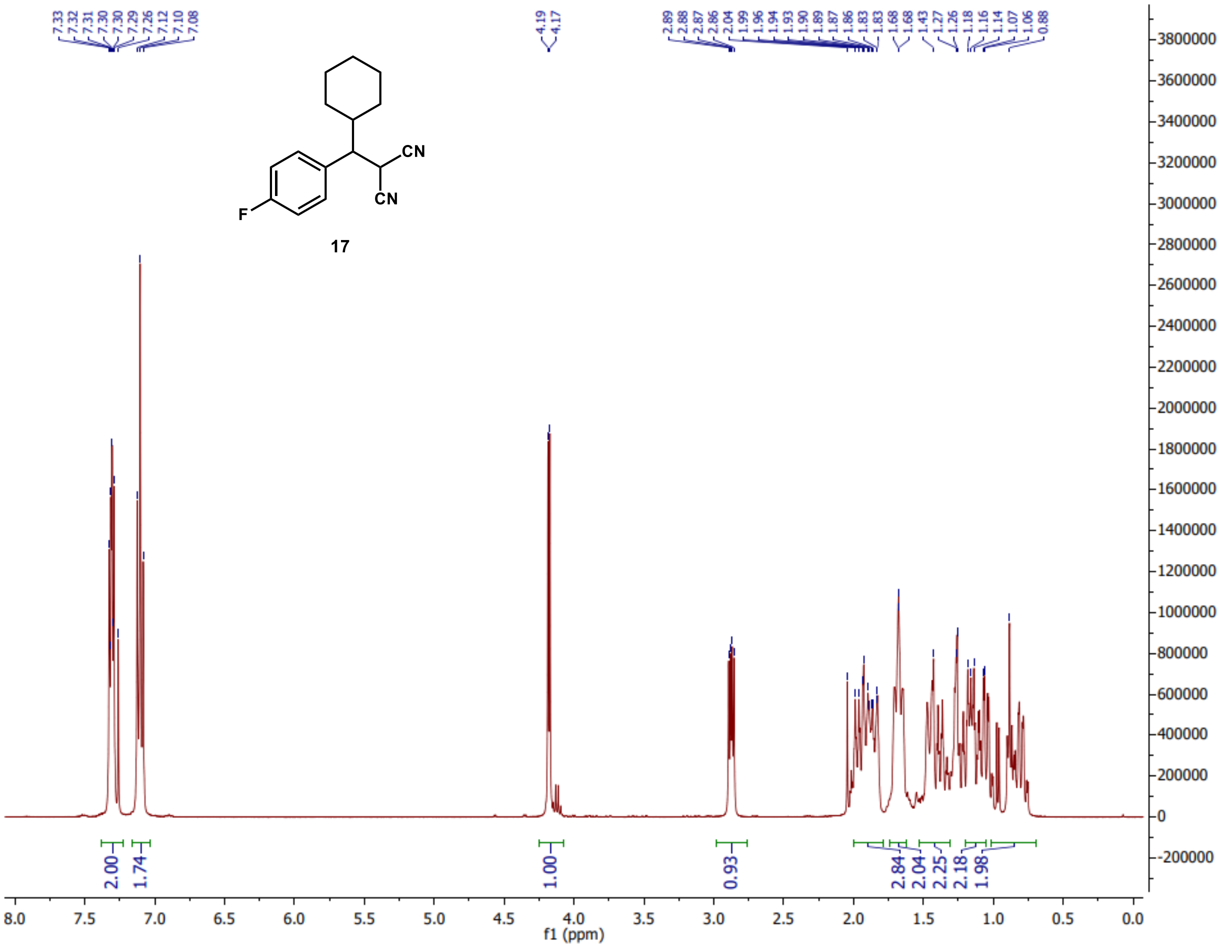

^{1}H (400 MHz) data for compound **18** in $CDCl_3$:

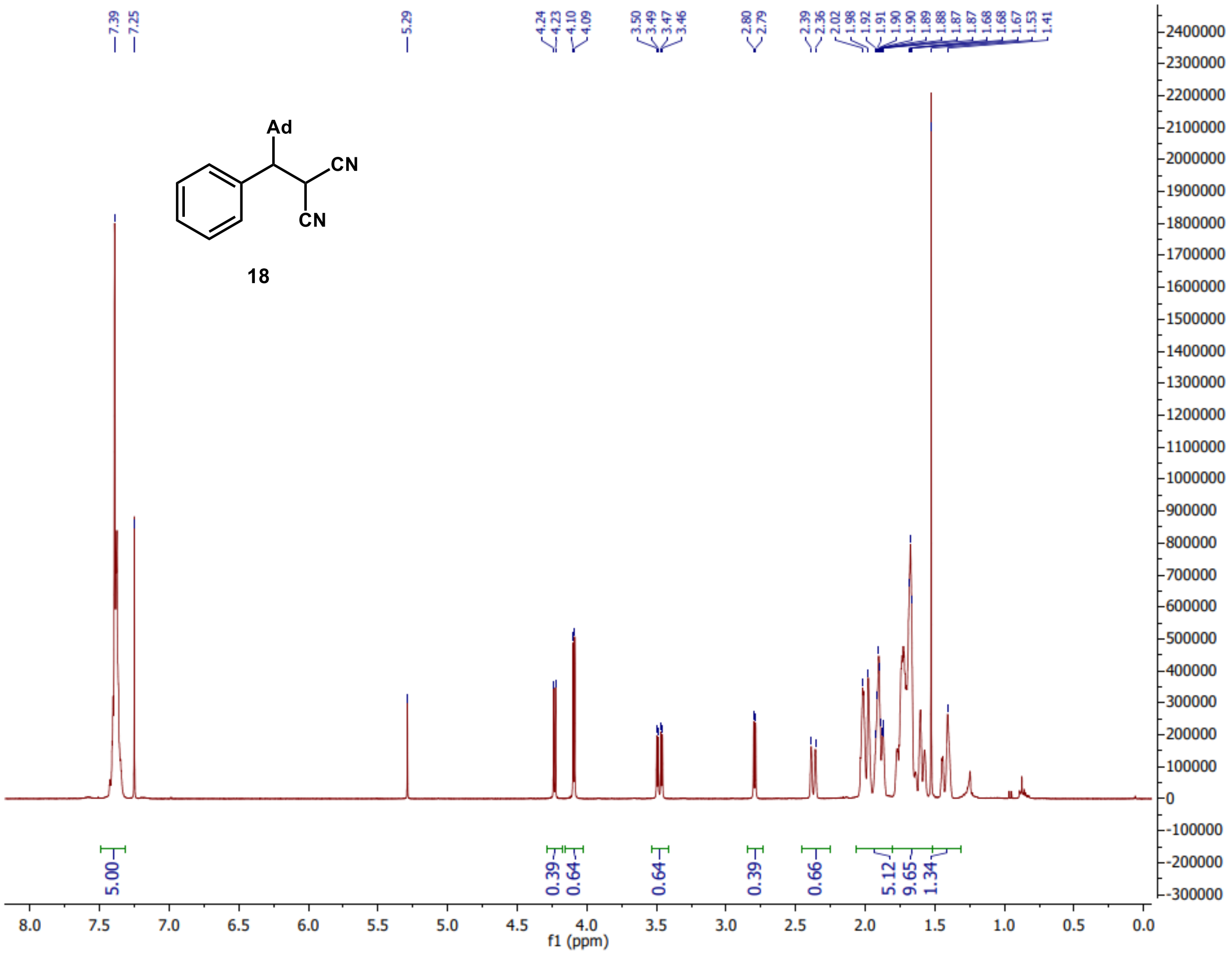

^{1}H (400 MHz) and $^{13}C\{^{1}H\}$ (101 MHz) data for compound **19** in $CDCl_3$:

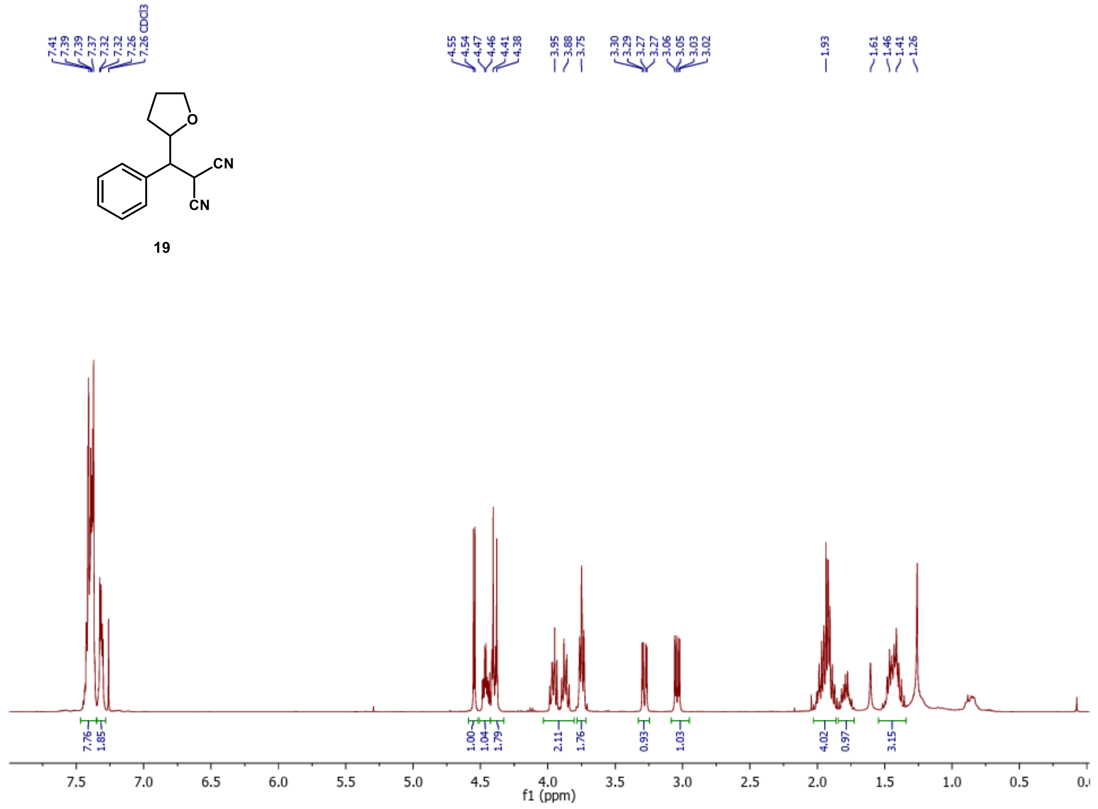

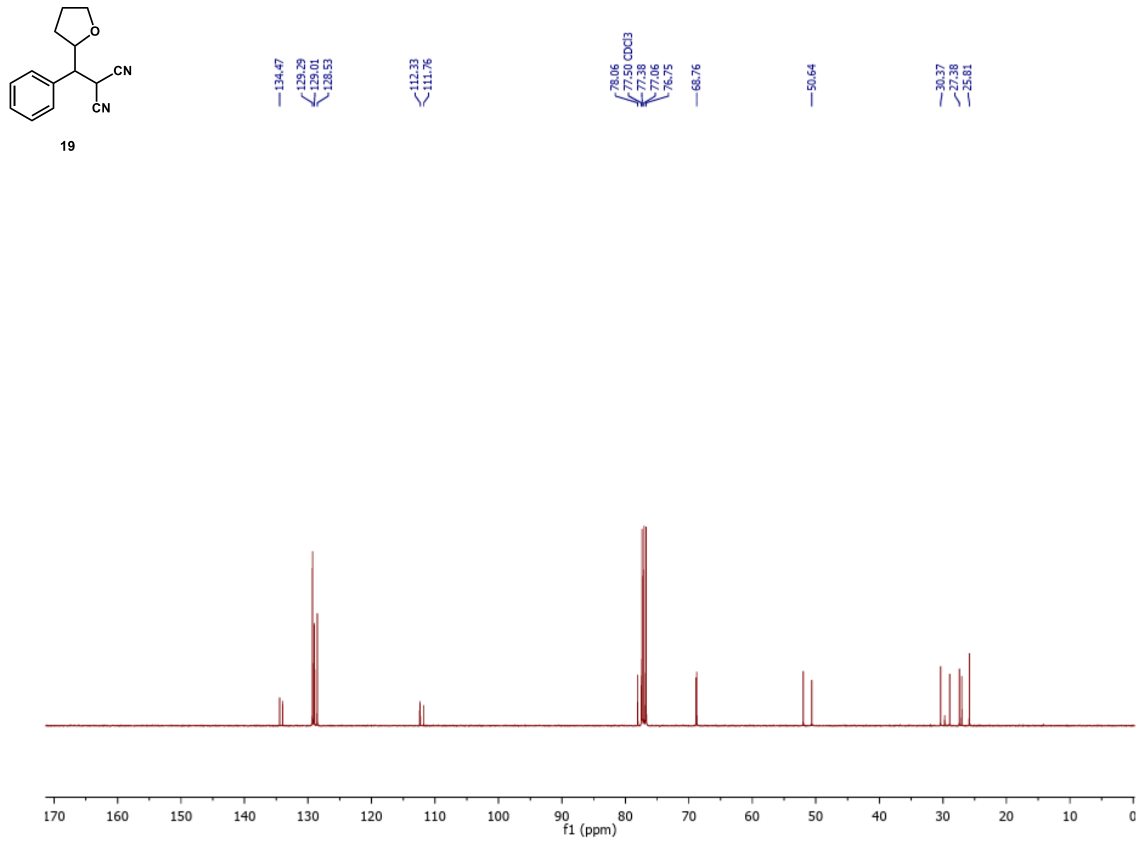

^{1}H (400 MHz) data for compound **20** in $CDCl_3$:

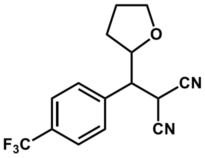

20

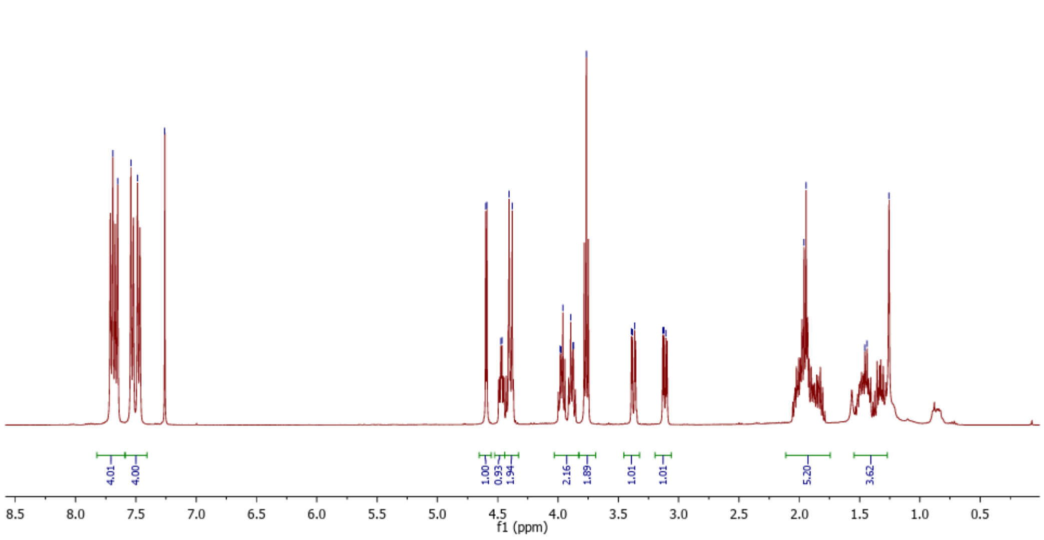

^{1}H (400 MHz) and $^{13}C\{^{1}H\}$ (101 MHz) data for compound **21** in $CDCl_3$:

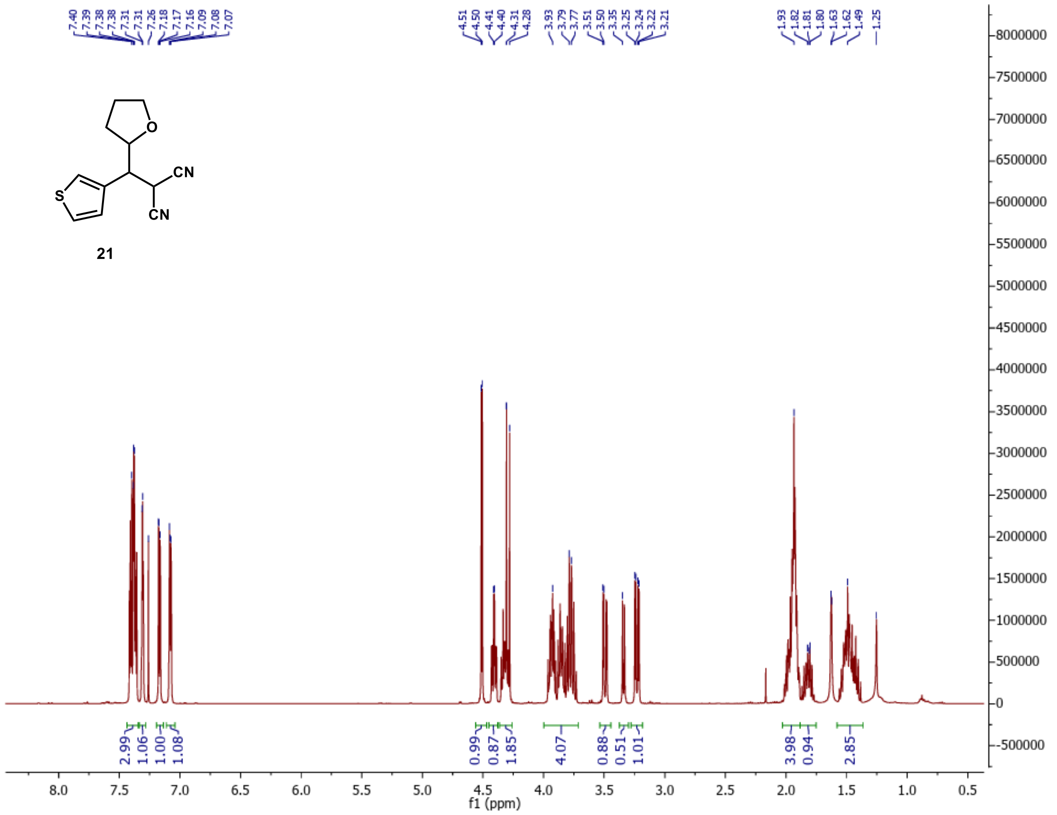

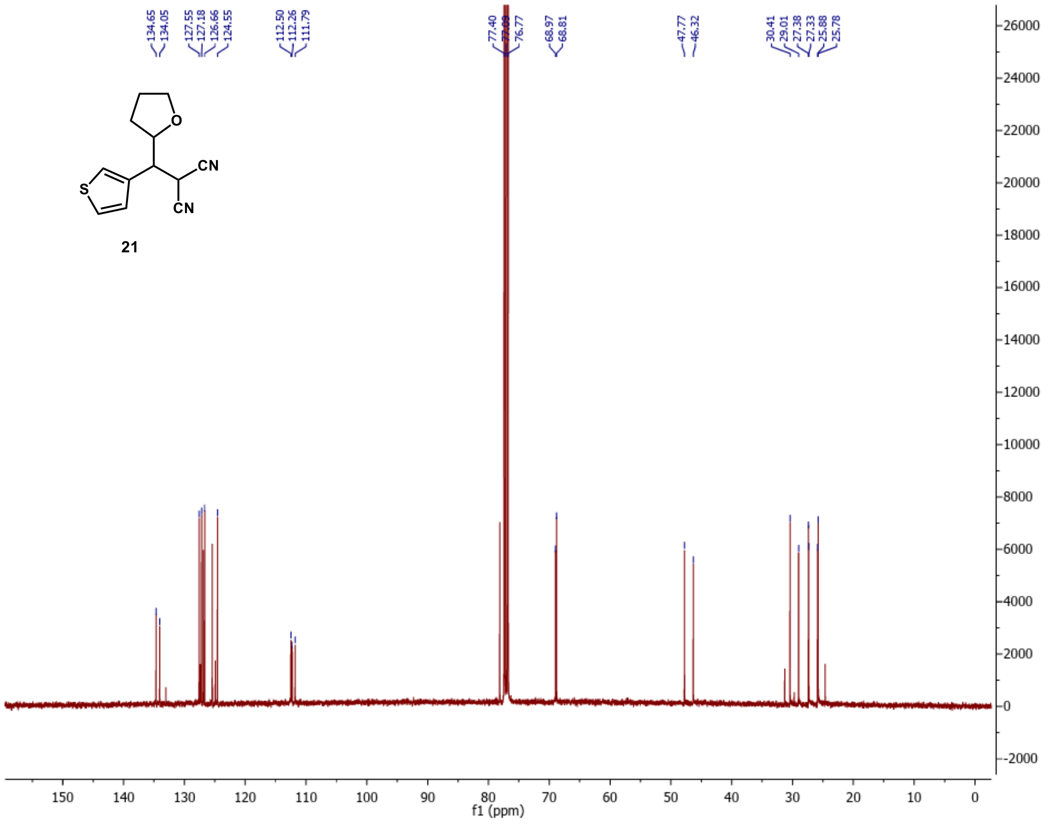

^{1}H (400 MHz) data for compound **22** in $CDCl_3$:

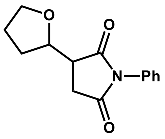

22

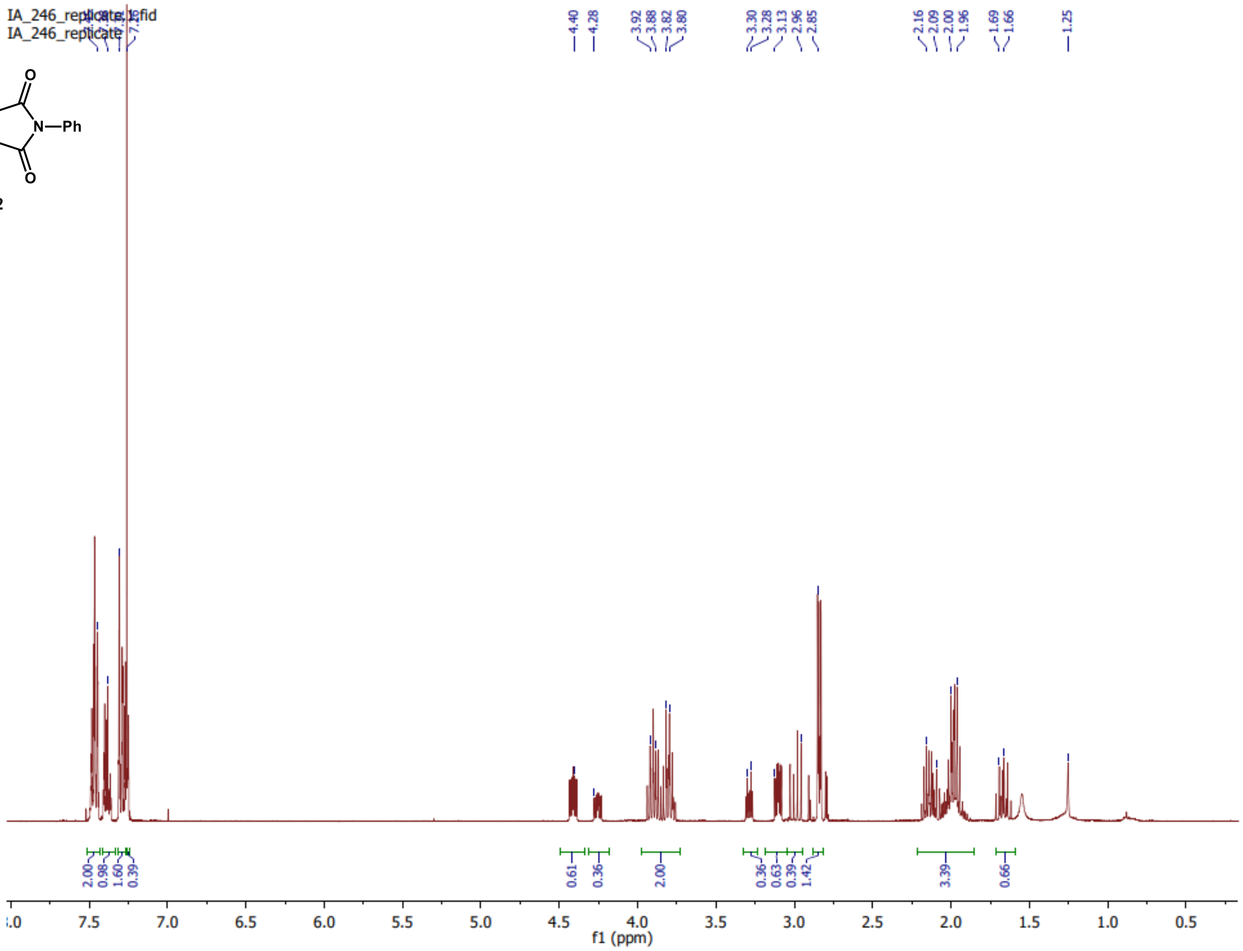

^{1}H (400 MHz) and ^{13}C{^{1}H} (126 MHz) data for compound **23** in $CDCl_3$:

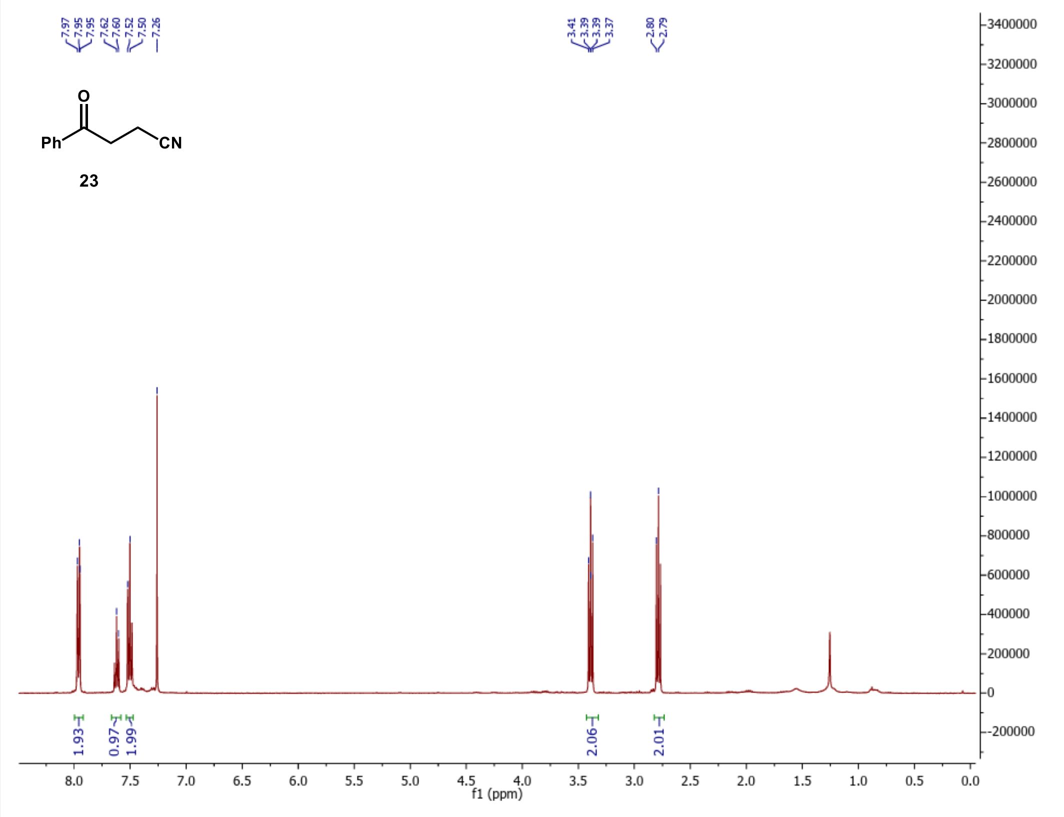

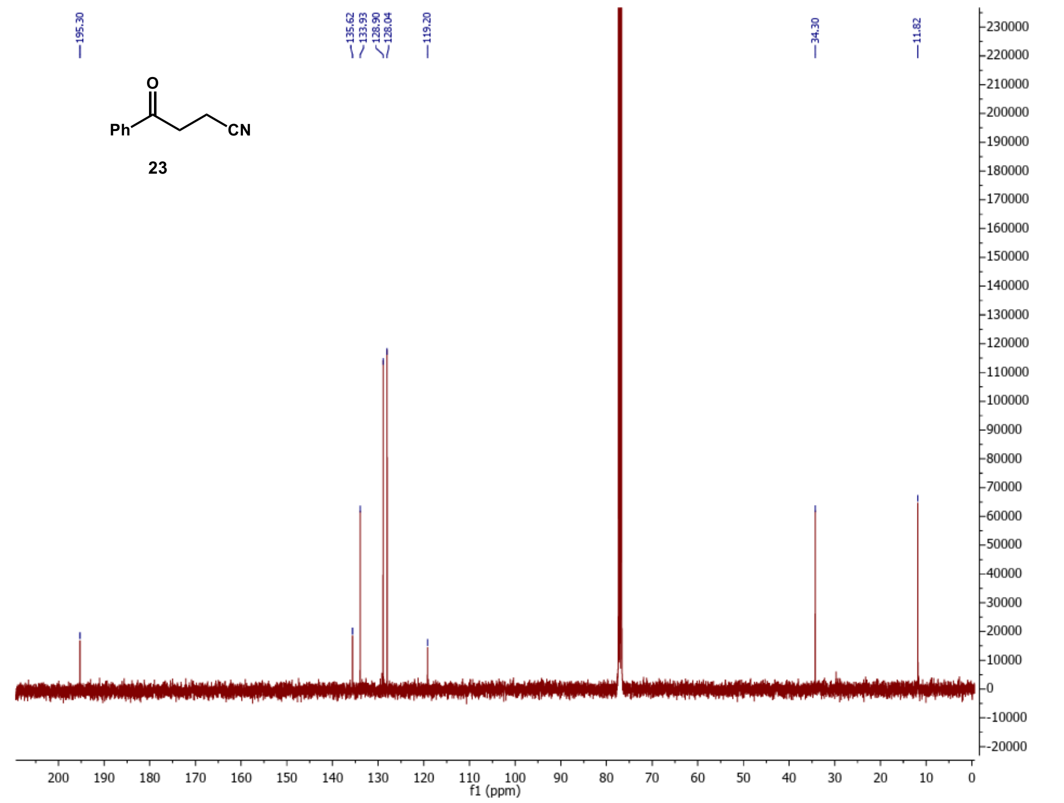

^{1}H (400 MHz) and $^{13}C\{^{1}H\}$ (126 MHz) data for compound **24** in $CDCl_3$:

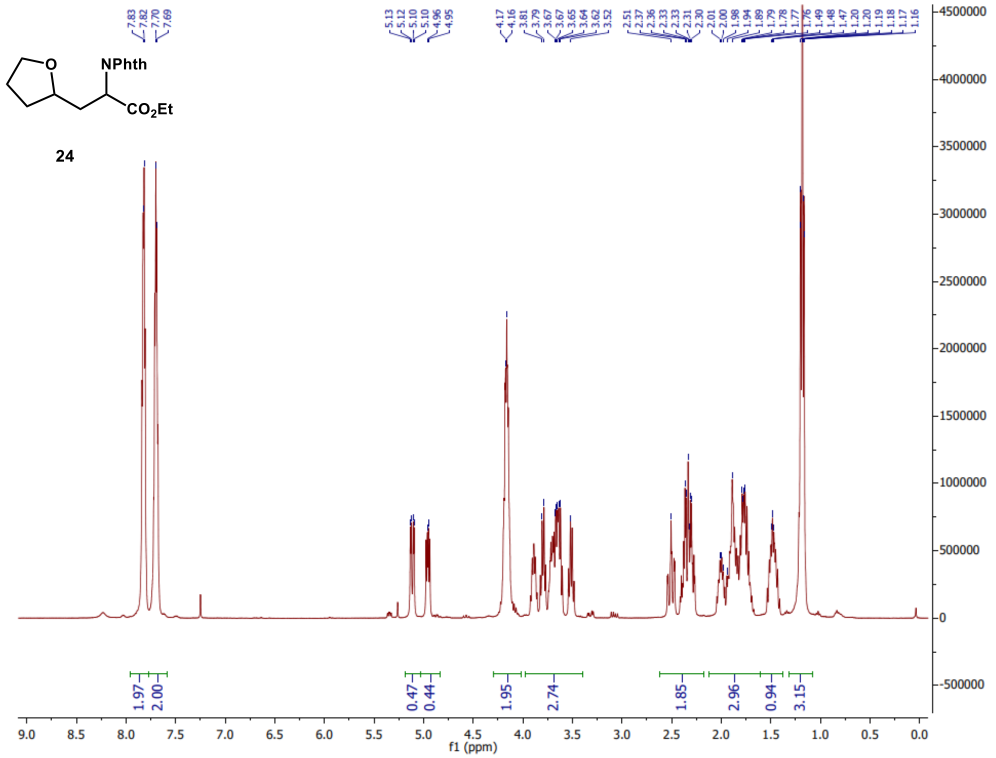

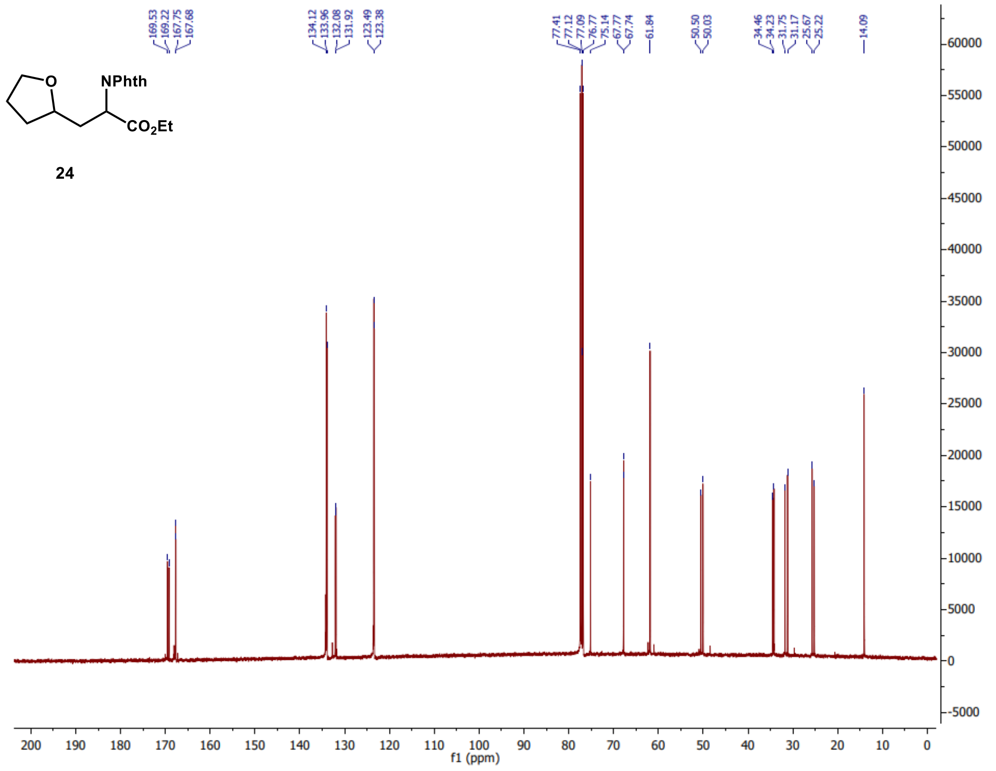

^{1}H (400 MHz) and $^{13}C\{^{1}H\}$ (126 MHz) data for compound **25** in $CDCl_3$:

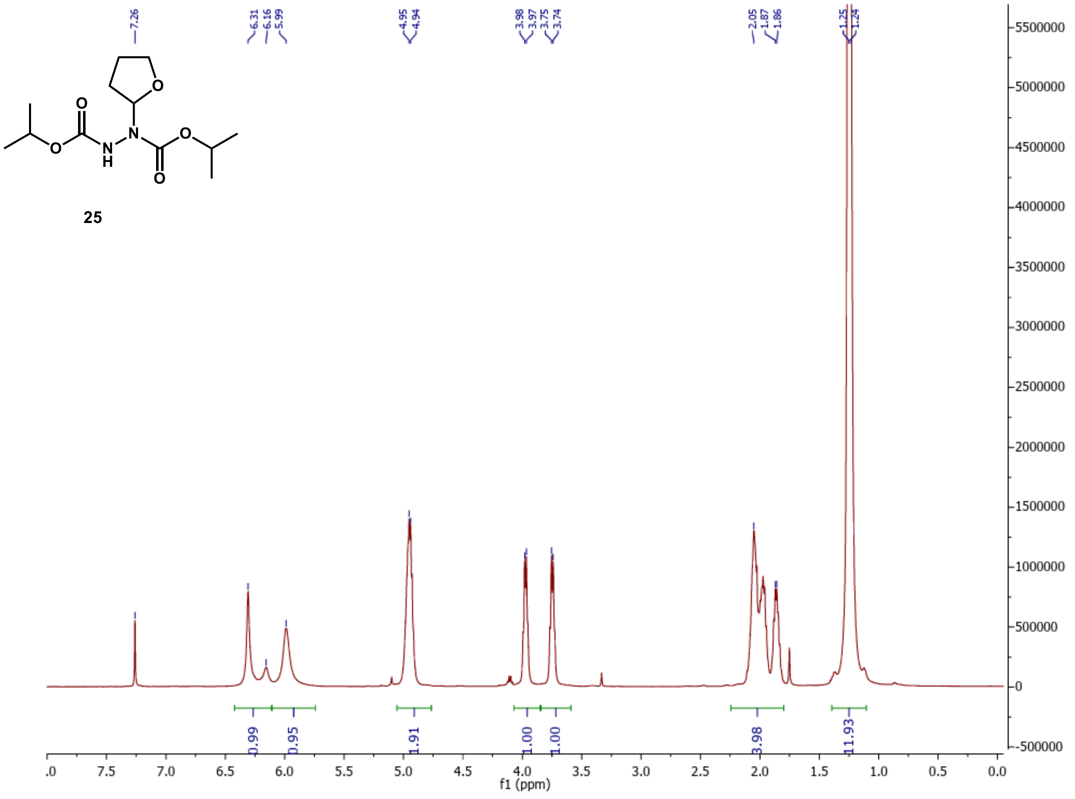

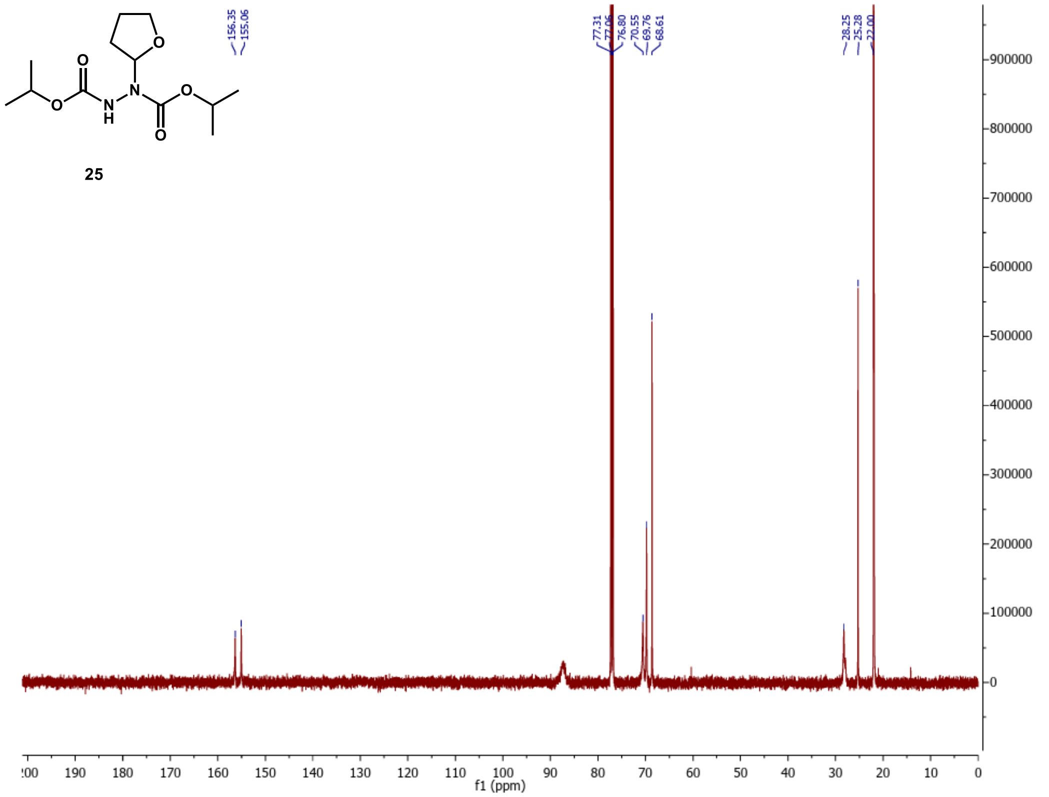

Supplement: Supplementary file 1 — jo4c03107_si_001.pdf [file jo4c03107_si_001.pdf]
